# Impact of Transition-State Aromaticity on Selective Radical–Radical Coupling of Triarylimidazolyl Radicals

**DOI:** 10.1021/jacs.4c14095

**Published:** 2024-11-28

**Authors:** Kazunori Okamoto, Sayaka Hatano, Manabu Abe

**Affiliations:** Department of Chemistry, Graduate School of Advanced Science and Engineering, Hiroshima University, 1-3-1 Kagamiyama, Higashi-Hiroshima 739-8526, Japan

## Abstract

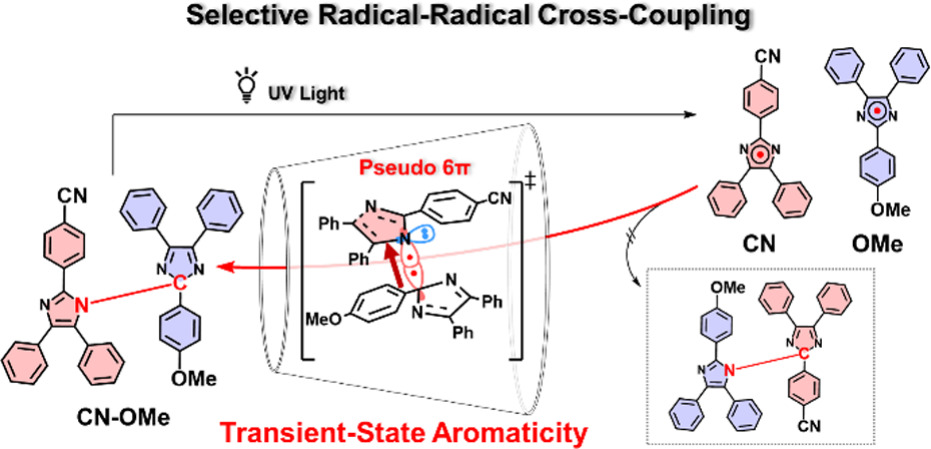

Radical coupling reactions are generally known to have a low selectivity due to the high reactivity of radicals. In this study, high regio and substrate selectivity was discovered in the dimerization of triarylimidazolyl radicals (**TAIR**), a versatile photochromic reaction. When two different radicals, 2-(4-cyanophenyl)-4,5-diphenyl-1*H*-imidazolyl radical (**CN-TAIR**) and 2-(4-methoxyphenyl)-4,5-diphenyl-1*H*-imidazolyl radical (**OMe-TAIR**), were simultaneously generated in situ, a hexaarylbiimidazole, formed by selective coupling at the nitrogen atom at position 1 of **CN-TAIR** and the carbon atom at position 2 of **OMe-TAIR**, was isolated with high selectivity as the main product among 24 possible radical dimer hexaarylbiimidazole derivatives. This high regio and substrate selectivity cannot be explained solely by the stability of the product and/or the electrophilicity and nucleophilicity of the radicals but originates from the aromaticity of the transition state in the radical–radical coupling reaction. To date, the selectivity of radical coupling reactions has been thought to be controlled by steric hindrance and radical spin density, but this study revealed a new factor for controlling radical coupling, that is, transition-state aromaticity. Aromaticity has been reported to have an important effect not only in the reactivity and structure of ground-state molecules but also on the electronically excited states and transition states in pericyclic reactions such as the Diels–Alder reaction and the Cope–Claisen rearrangement. This study demonstrated for the first time that radical coupling reactions can also be controlled by transition-state aromaticity.

## Introduction

**Scheme 1:**
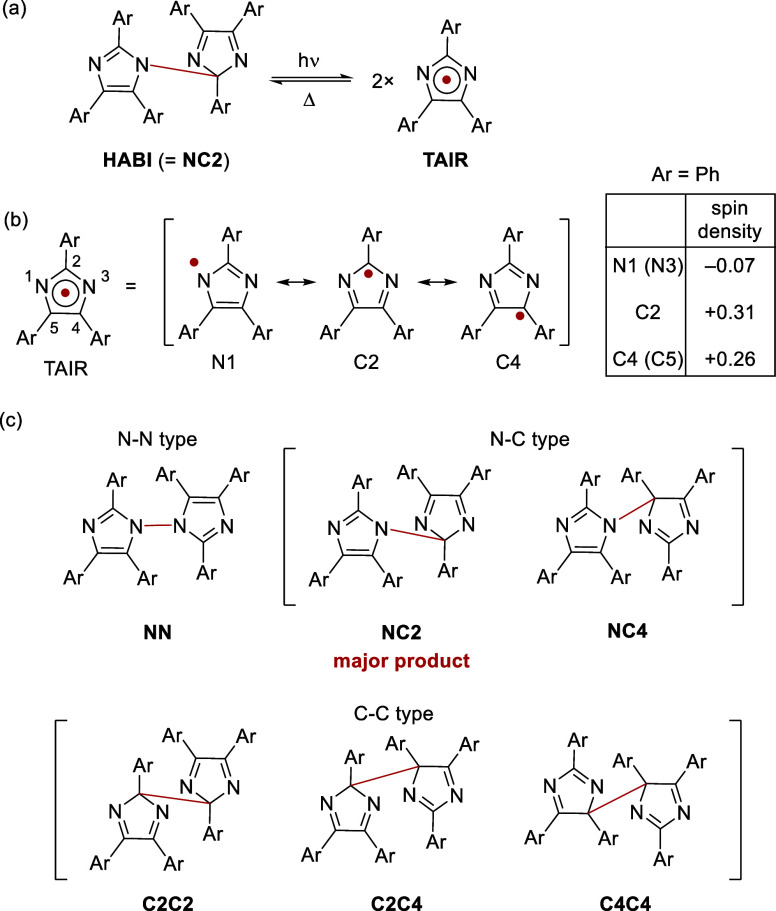
(a) Photochromic Reaction of **HABI,** and (b) Spin-Resonance Structures and Spin Density of **TAIR**, and (c) Possible HABI Isomers with Different Bond Positions Produced by Radical Coupling at N1, C2, and C4

**Scheme 2:**
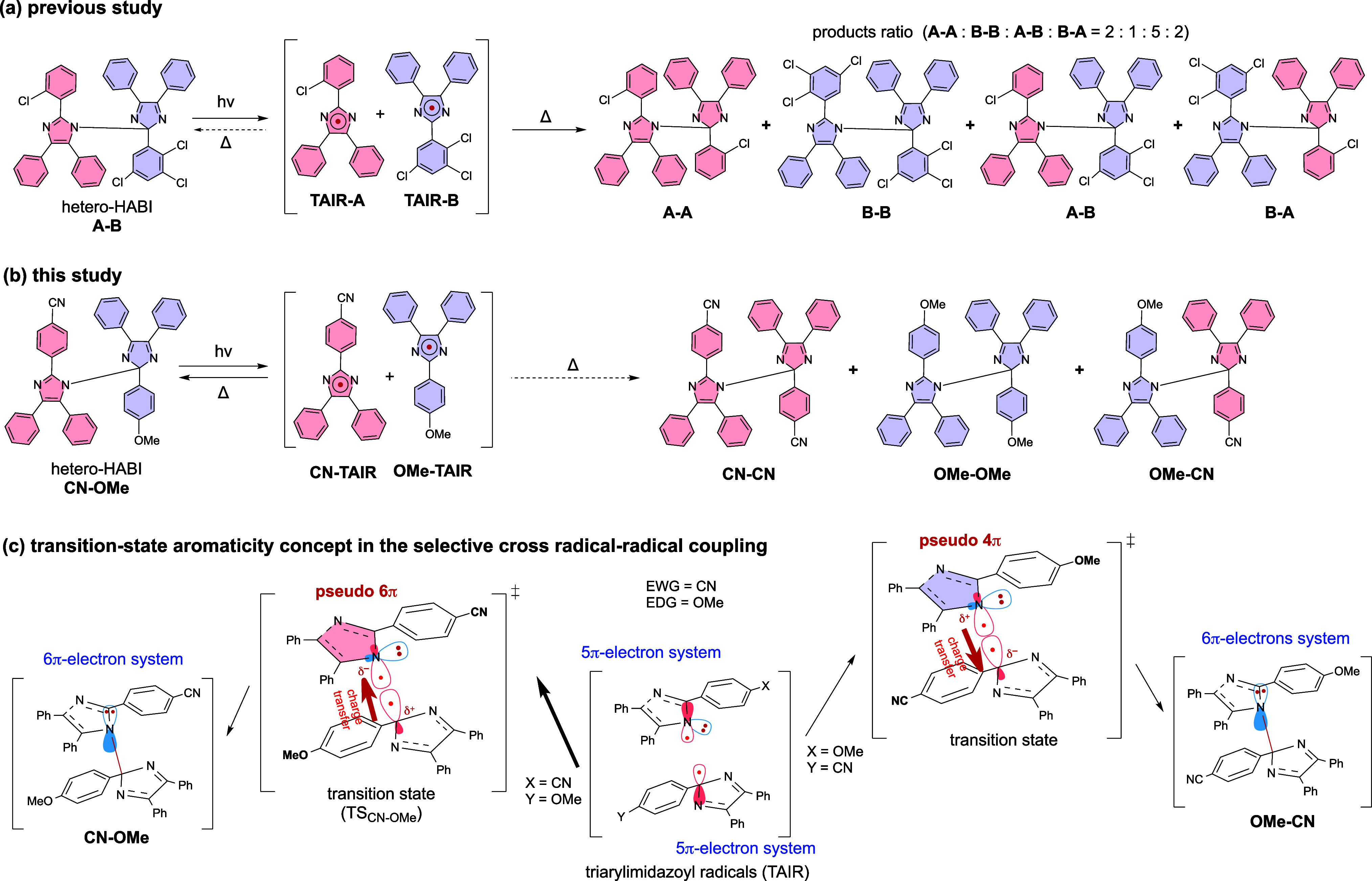
(a) Previous Study: Irreversible Reaction of Hetero-HABI; (b) This Study: Reversible Generation and Recombination of **CN-TAIR** and **OMe-TAIR**; (c) TS Aromaticity: Mechanism of Selective Formation of **CN-OMe**

The important roles of highly reactive radicals for polymerization^1−5^ and in vivo reactions^6−8^ as well as synthetic chemistry^9−15^ have recently been revealed; thus, elucidating the elusive structure and reactivity of radicals is an extremely important research topic. In addition, radical species absorb and emit light in the visible to near-infrared region, making them useful as bioimaging^16−18^ and photochromic materials.^19^ For example, hexaarylbiimidazole (**HABI**) is a photochromic molecule discovered by Maeda, Hayashi, in the 1960s.^20−25^ Under photoirradiation, the covalent bond between nitrogen and carbon (N–C) in **HABI** is homolytically cleaved to produce the colored 2,4,5-triarylimidazolyl radical (**TAIR**) with an absorption maximum around 550–600 nm (Scheme 1a).^26,27^ The spin density of **TAIR** is delocalized over the five-membered ring, and the radical has three reactive sites, N1 (=N3), C2, and C4 (=C5). Therefore, in the intermolecular dimerization of **TAIR**, six regio isomers can be generated: N–N-type N1–N1′-HABI (**NN**), in which N atoms are bonded together; N–C type N1–C2′-HABI (**NC2**) and N1–C4′-HABI (**NC4**), in which N and C atoms are bonded together; and C–C type C2C2′-HABI (**C2C2**), C2–C4′-HABI (**C2C4**), and C4–C4′-HABI (**C4C4**), in which C atoms are bonded together (Scheme 1b).^23,28−38^ However, in the radical recombination of **TAIR** at room temperature, **NC2** with an N1–C2’ bond is selectively formed, and a reversible photochromic reaction is achieved. The selectivity of the bonding site is explained by the product stability, spin density, and steric effect. Compound **NN** is well stabilized by the two aromatic imidazole rings, but the spin density on the N atom of **TAIR** is extremely low because the N atom cannot resonate with the three aryl groups. In addition, the repulsion between the lone-pair electrons on N atoms is severe to increase the activation energy of the radical coupling reaction. Thus, the formation of **NN** molecules has not yet been confirmed. On the other hand, the spin density on the carbon of **TAIR** is higher than that on the nitrogen atom due to the resonance effect with the aryl (Ar) group, making the C–C radical coupling reaction kinetically favorable. Thus, **C2C2** with the highest spin density has been reported at low temperatures. However, because the five-membered ring of **C2C2** lacks aromaticity, **C2C2** is isomerized to the more thermodynamically stable **NC2** unless there is low spin density on the N atom. Introducing a bulky substituent on the C2 carbon suppresses the formation of **NC2**, resulting in the production of **NC4** and **C4C4**.^38,39^ Thus, because the dimerization of **TAIR** involves both kinetically and thermodynamically controlled processes, investigating not only the substituent effect but also the temperature effect is crucial for understanding the reaction of **TAIR** as a photochromic molecule. Abe and co-workers reported ultrafast photochromic reactions in molecules in which two **TAIR** molecules are fixed intramolecularly with a linker such as naphthalene.^40−42^ In addition, it was shown that the hetero-**HABI A-B** under photoirradiation did not return to the original molecule **A-B**, but instead of a mixture of **A-A**, **B-B**, **A-B**, and **B-A** was obtained in 2:1:5:2 ratio through radical coupling reactions (Scheme 2a).^43^ Photoirradiation of synthesized **B-A** also produced the four products in the same ratio. As such, even if the radical is relatively stable, it is usually difficult to obtain selective products in radical recombination reactions due to the low activation energy. In this study, **CN-TAIR**, which has a CN group as the electron-withdrawing group (EWG), and **OMe-TAIR**, which has an OMe group as the electron-donating group (EDG), are generated by the photoreaction of hetero-HABI (**CN-OMe**) to understand the radical behavior in more detail in radical–radical coupling reactions. The NC2-type hetero-HABI (**CN-OMe**), in which N1 of **CN-TAIR** is bonded to C2 of **OMe-TAIR**, is selectively obtained as a product that could be isolated at room temperature (Scheme 2b). At first glance, this dimerization reaction seems to be explained by the electronically matched case between the nucleophilic **CN-TAIR** and the electrophilic **OMe-TAIR**, but the hetero-HABI (**OMe-CN**) bonded at C2 of **CN-TAIR** and N1 of **OMe-TAIR** was not produced. Thus, high selectivity for the reaction center was observed, where **CN-TAIR** consistently reacts at N1 and **OMe-TAIR** reacts at C2. To clarify the origin of the selectivity of this interesting phenomenon, detailed product analysis and quantum chemical calculations of the photodissociation reaction of **CN-OMe** at low temperatures are performed in this study, demonstrating that the selective formation of **CN-OMe** is due to charge transfer from C2 of **OMe-TAIR** to N1 of **CN-TAIR** in the transition state (**TS**_CN-OMe_) formed during the dimerization reaction in which the N1–C2 σ-bond is formed, and that the aromaticity of the transition state is largely contributed (Scheme 2c).

The transition-state (TS) aromaticity on the reactivity of pericyclic reactions such as the Diels–Alder reaction was reported by Evans and Warhurst in 1938.^44,45^ This selective formation of **CN-OMe** clarifies that the TS aromaticity also plays a crucial role in determining the selectivity of the radical recombination reactions, in which the highly reversible reaction of hetero-HABI was found for the first time. This new factor, TS aromaticity, will not only greatly contribute to the molecular design and reactions of new photochromic molecules, enabling control of the emission color under photoirradiation, but is also a prospectively effective method of controlling radical coupling reactions, which are usually difficult to control.

## Results and Discussion

### Synthesis and Structural Characterization of HABI Derivatives CN-CN, OMe-OMe, and CN-OMe

**Figure 1:**
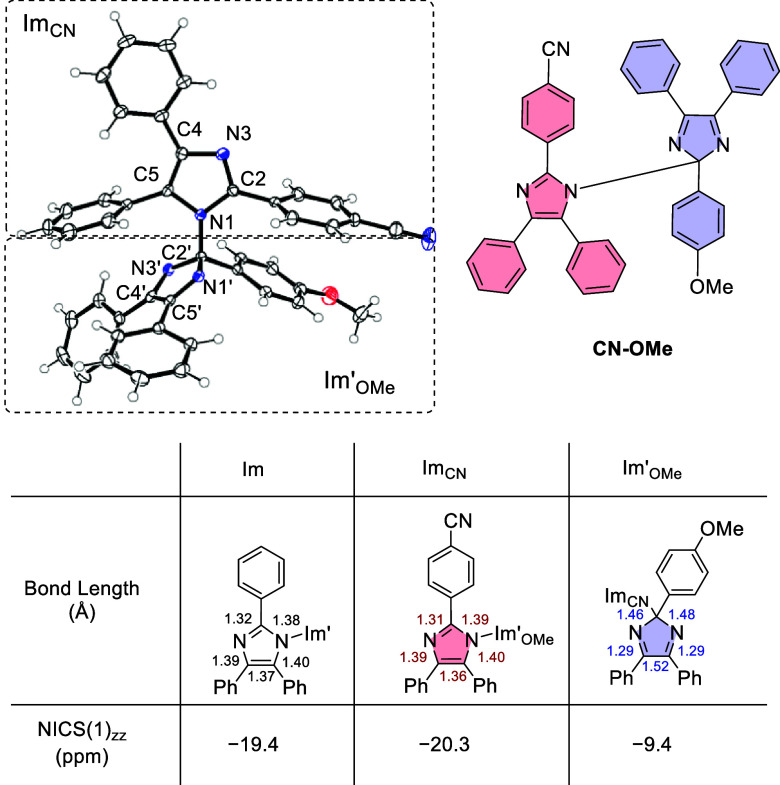
X-ray crystal structure of the **CN-OMe** form of hetero-HABI with thermal ellipsoids (50% probability), where nitrogen and oxygen atoms are highlighted in blue and red, respectively. Bond length for nonsubstituent imidazole units, Im, ImCN, and Im’OMe from X-ray crystal structure, where NICS(1)_*zz*_ value was calculated using DFT at the B3LYP-D3/6-31G(d) level for each structure.

As precursors for generating **CN-TAIR** and **OMe-TAIR**, HABI derivatives **CN-CN**, **OMe-OMe**, and **CN-OMe** were synthesized by the oxidation of the corresponding triaryl imidazoles **CN-TAI** and **OMe-TAI**, respectively, with potassium ferricyanide in 99, 99, and 82%, respectively (Figures S1–S18). The structures of **CN-CN**, **OMe-OMe**, and **CN-OMe** were determined by single-crystal X-ray structural analysis (Figure 1, Tables S1–S3). The length of the N1–C2’ bond in **CN-OMe** is 1.475 Å, which is typical for N–C single bonds. This bond length is not significantly different from the bond lengths of **CN-CN** and **OMe-OMe** (1.472 and 1.477 Å, respectively). The bond lengths of N1–C2, C2–N3, N3–C4, C4–C5, and C5–N1 in the imidazole ring (Im_CN_) bonded at the nitrogen side (N-unit) are 1.39, 1.31, 1.39, 1.36, and 1.40 Å, respectively, which are close to the bond lengths of unsubstituted triaryllimidazole (H-TAI).^46,47^ The N1–C5 (=N3–C4) bond in the five-membered ring (Im’_OMe_) of the unit bonded at the carbon side (C-unit) has strong double-bond character (1.289 Å), whereas the other bonds (N1–C2, C2–N3, and C4–C5) have close to single-bond character (1.463, 1,477, and 1.515 Å), where the aromaticity does not exist. Thus, NICS(1)_*zz*_^48,49^ of Im_CN_ showed a large shielding effect (NICS(1)_*zz*_ = −20.3 ppm) similar to that of the imidazole ring (Im) of H-TAI, but aromaticity was not confirmed in Im’_OMe_ (NICS(1)_*zz*_ = −9.4 ppm). The shielding effect of the imidazole ring of **CN-CN** and **OMe-OMe** was −19.7 and–19.9 ppm, respectively, based on NICS(1)_*zz*_, and there was no significant difference in the aromaticity of molecules (Figure S44).

### Thermodynamics and Kinetics of Photoreaction

**Figure 2:**
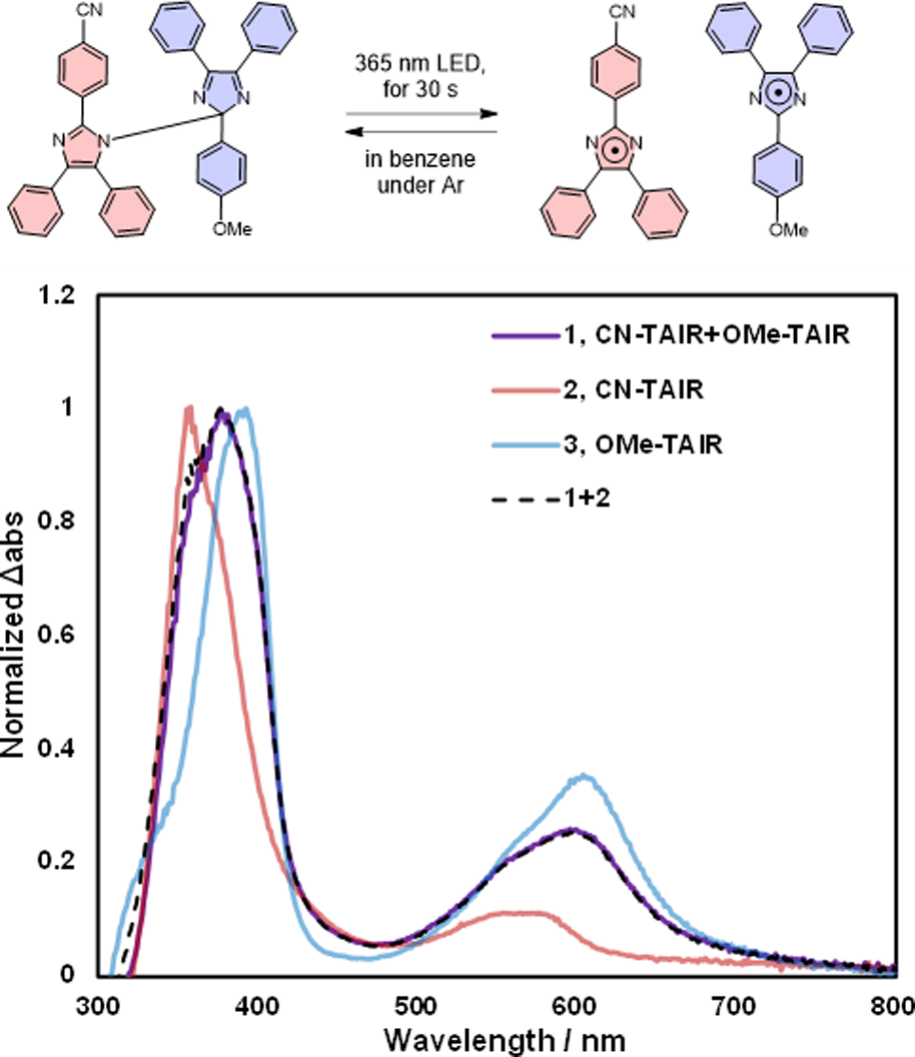
Spectrum 1 is the UV–vis absorption spectrum of **CN-OMe** after photoirradiation in degassed benzene. Spectra 2 and 3 are the spectra of pure **CN-TAIR** and **OMe-TAIR** produced from **CN-CN** and **OMe-OMe**. Black dotted line is the sum of spectra 1 and 2. All spectral data are normalized to abs = 1.

**Figure 3:**
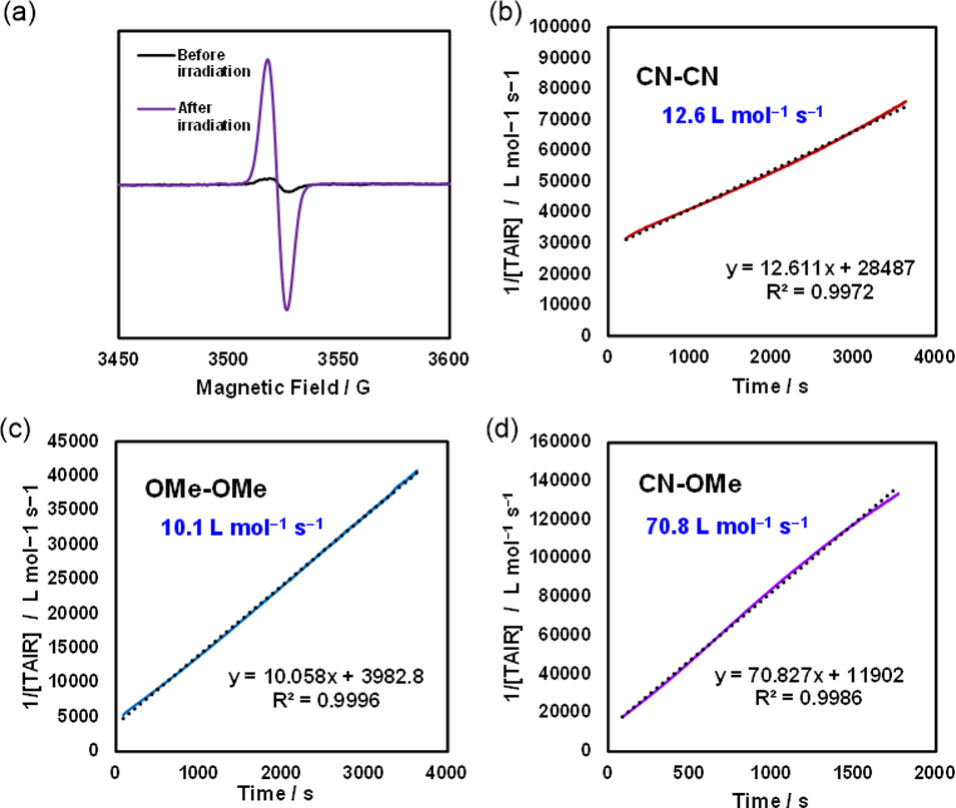
(a) EPR spectra of **CN-OMe** in degassed benzene before and after photoirradiation at 293 K. EPR decay profiles of (b) **CN-TAIR** + **OMe-TAIR**, (c) **CN-TAIR**, and (d) **OMe-TAIR**. Each radical was generated by irradiation (30 s with a 365 nm LED) of **CN-CN**, **OMe-OMe**, and **CN-OMe**, respectively.

**Figure 4:**
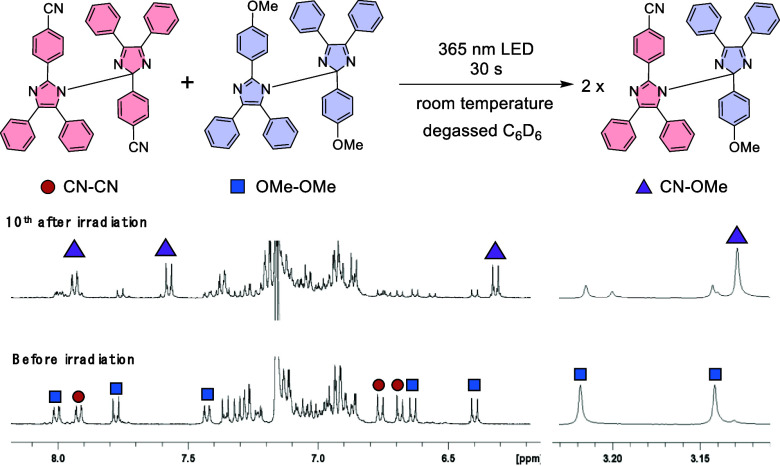
^1^H NMR spectra of mixed solution of **CN-CN** and **OMe-OMe** in a 1:1 radio before and 10th after irradiation cycle. Purple triangle = **CN-OMe**, red circle = **CN-CN**, and blue square = **OMe-OMe**.

The different radicals **CN-TAIR** and **OMe-TAIR** generated by photoirradiation of **CN-OMe** and their reactivity were determined from the UV–visible absorption spectra in benzene, which has a low reactivity with radicals (Figure 2). Each peak was normalized to the absorption maximum wavelength of 1. Before irradiation, **CN-OMe** was absorbed mainly in the ultraviolet region (Figure S19) and was pale yellow in solution. When a degassed benzene solution of **CN-OMe** was irradiated for 30 s using a 365 nm LED lamp, the solution color immediately changed to blue, and characteristic peaks of the imidazolyl radical (**TAIR**) were observed at 380 and 600 nm. Electroparamagnetic resonance (EPR) measurements confirmed a doublet peak due to free radicals (Figure 3a). The generated free radicals gradually decayed with second-order kinetics at room temperature, reaching completion within 30 min. A similar observation was found in UV–vis absorption analysis. When **CN-CN** and **OMe-OMe** were photoirradiated under the same conditions, the absorption signals of pure **CN-TAIR** and **OMe-TAIR** were observed at 355, 560, and 390, and 610 nm, respectively (Figure 2, red and blue lines). The absorption spectrum of **CN-OMe** generated by photoirradiation (Figure 2, purple line) showed good agreement with the absorption spectrum of **CN-TAIR** and **OMe-TAIR** combined in a one-to-one ratio (Figure 2, black dotted line). This indicates that **CN-TAIR** and **OMe-TAIR** are generated simultaneously upon the photoirradiation of **CN-OMe**. To analyze the kinetics of the generated radicals, EPR data were acquired after photoirradiation of **CN-CN**, **OMe-OMe**, and **CN-OMe** (Figures 3, S21–S27)). In the EPR kinetic analysis, the homodimerization reaction of the photogenerated **CN-TAIR** and **OMe-TAIR** followed second-order kinetics at 293 K.^50−53^ The deactivation reaction rate constants, *k*_CN_ and *k*_OMe_, of **CN-TAIR** and **OMe-TAIR** generated from **CN-CN** and **OMe-OMe** were determined to be 12.6 and 10.1 L mol^–1^ s^–1^, respectively (Figure 3b,c), which are smaller than the value of 46.0 L mol^–1^ s^–1^ for the parent H-**TAIR** (Figure S30). The decay of the EPR signal of the mixed solution of **CN-TAIR** and **OMe-TAIR** generated by photoirradiation of **CN-OMe** followed second-order reaction kinetics (Figure 3d). Assuming that the two **TAIR** molecules, **CN-TAIR** and **OMe-TAIR**, existed in the same ratio (1:1), second-order reaction rate constant *k*_**CN-OMe**_ = 70.8 L mol^–1^ s^–1^ was obtained. This reaction rate constant for radical recombination is higher than those of **CN-TAIR** and **OMe-TAIR**. To analyze the products of the photoreaction of the three types of **HABI** (**CN-OMe**, **CN-CN**, and **OMe-OMe**), the samples were photoirradiated 10 times for 30 s, and the changes in the NMR spectra of the reaction products were monitored. Three compounds, **CN-OMe**, **CN-CN**, and **OMe-OMe**, quantitatively returned to the original **HABI** compound after the photoreaction, and the NMR spectrum did not change (Figures S28–S30). In addition, a sample was prepared by mixing **CN-CN** and **OMe-OMe** in a 1:1 ratio, and the photoreaction products were analyzed (Figure 4). As with the photoreaction of the single molecule, after 10 rounds of photoirradiation for 30 s, the intensity of the peaks of **CN-CN** and **OMe-OMe** decreased, and instead, peaks of **CN-OMe** appeared, indicating that cross-coupling occurs preferentially to form **CN-OMe**, rather than two **HABI** molecules **CN-CN** and **OMe-OMe**. This is consistent with the kinetic analysis of the EPR data. The results of UV–vis, EPR, and NMR measurements experimentally demonstrated that the two different radicals react selectively to produce cross-coupling compound **CN-OMe** at room temperature.

### Selectivity in Radical–Radical Coupling at Low Temperature

**Figure 5:**
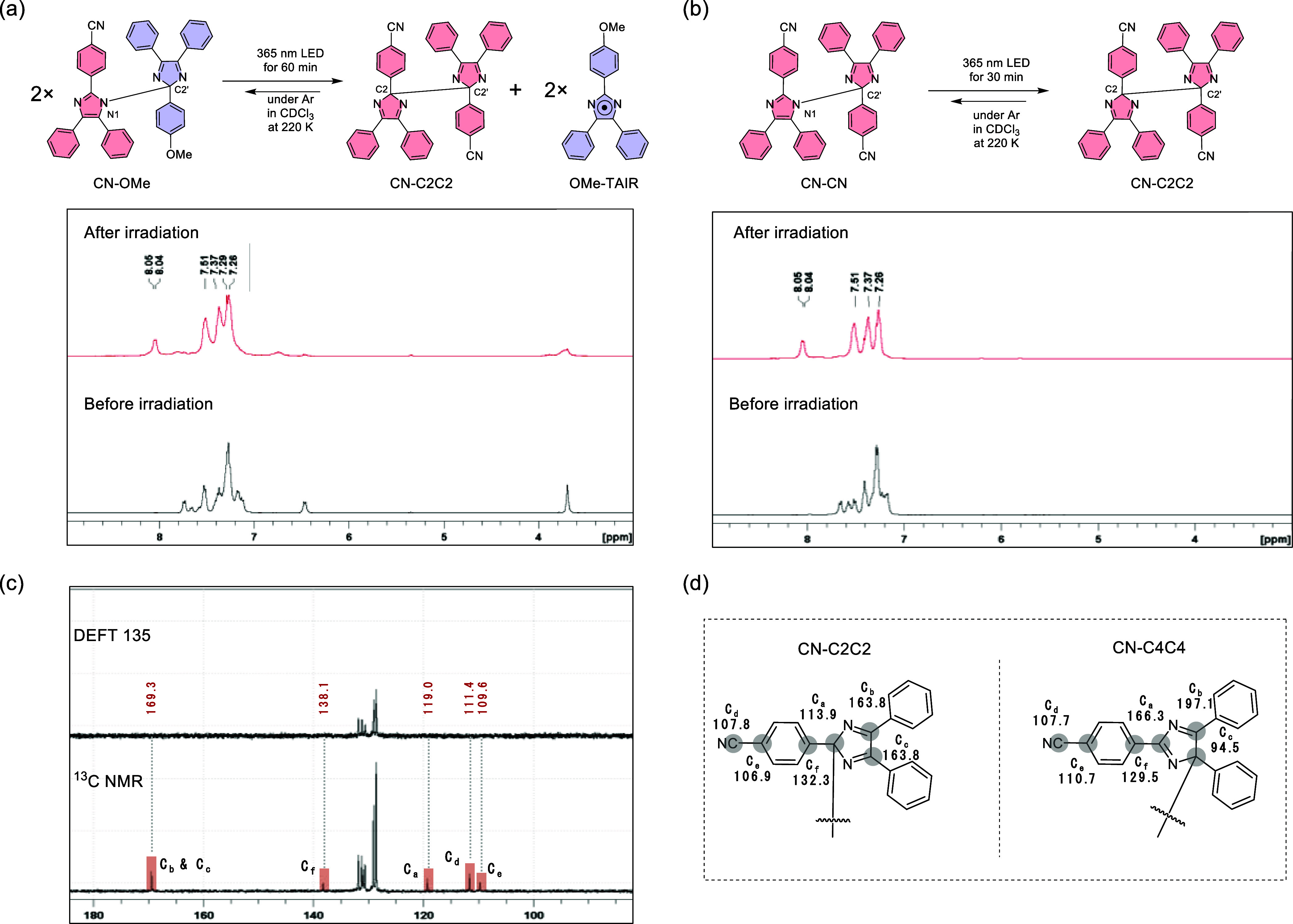
(a) ^1^H NMR spectrum of **CN-OMe** and (b) **CN-CN** before and after irradiation with a 365 nm LED light at 220 K in CDCl_3_ under Ar. (c) ^13^C NMR and DEFT 135 spectrum of the photoproduct of **CN-OMe** after photoirradiation at 220 K. (d) ^13^C NMR data of CN–C2C2 and CN–C4C4 were simulated at the B3LYP-D3/6-31G(d) level.

As mentioned in the Introduction, the products of the radical recombination of **TAIR** are temperature-dependent. At room temperature, thermodynamically stable N–C-type HABI is produced, whereas at low temperatures, C–C-type HABI **C2C2** is obtained as the main product.^38^ The photoreaction products of **CN-CN**, **OMe-OMe**, and **CN-OMe** at low temperatures were investigated by using ^1^H and ^13^C NMR (Figure 5). The sample in CDCl_3_ solution was photoirradiated with a probe at 220 K under an Ar atmosphere, and the low-temperature NMR measurements were conducted. After irradiating **CN-OMe** with a 365 nm LED lamp, the NMR signals of the starting material decreased, and new signals appeared in the aromatic region (8.05, 8.04, 7.51, 7.37, and 7.26 ppm) (Figure 5a). In addition, several broad peaks were observed at 7.8, 6.7, and 3.8 ppm. To identify the product, a low-temperature photoreaction of **CN-CN** was performed under the same conditions, generating a product with a similar NMR signal in the aromatic region (Figure 5b). The low-temperature photoreaction of **OMe-OMe** was also evaluated under the same conditions. The peaks of the starting material decreased with prolonged photoirradiation, but no new NMR signals were observed. Therefore, it is believed that most of the **OMe-TAIR** remained in the solution at this temperature and was not detected by NMR (Figure S37). Indeed, the low-temperature photoreaction of **OMe-OMe** was monitored by UV–vis spectroscopy, and radical **OMe-TAIR** was observed as a persistent species at least within 30 min (Figure S39). The low-temperature photoreaction products of **CN-OMe** and **CN-CN** were identified by ^13^C NMR and two-dimensional NMR. NMR analysis revealed that the product was **CN-C2C2**, in which **CN-TAIR** was bonded together at the carbon-2 position (Figure 5c and Figures S32–35). To confirm the possibility of other C–C type isomers, the calculated^54^ and experimental ^13^C NMR signals of **CN-C2C2** and **CN-C4C4** of **CN-TAIR** were compared (Figure 5d). Here, only the characteristic peaks derived from quaternary carbons (C_a_, C_b_, C_c_, C_d_, C_e_, and C_f_) were detected in the DFPT 135 analysis. The simulated NMR peaks showed the best agreement with the peaks of **CN-C2C2**. The calculated signal of the C_a_ carbon in the five-membered ring was 113.9 ppm, and the value for the equivalent C_b_ and C_c_ was 163.8 ppm. The calculated results correspond to the experimental values of 119.0 and 169.3 ppm of quaternary carbons. The NMR signals of C_e_ and C_f,_ which are quaternary carbons of the CN-Ph group, and C_d_ of the CN group were also observed. The signals of other quaternary carbons could not be clearly observed because they overlapped with the other peaks. The peaks of carbon (C_a_, C_b_, C_c_) in the five-membered ring were significantly different for **CN-C2C2** versus **CN-C4C4**, suggesting a change in the electronic structure due to the difference in the bonding point. A highly low-field shift of the peak of the C_b_ carbon in **CN-C4C4** to 197.1 ppm was predicted. In the ^13^C NMR of **C4C4** of other TAIR derivatives analyzed in prior studies, a low-field peak shift originating from this carbon (C_b_) was indeed observed.^38,39^ This highly low-field shift is thought to be related to the contribution of ionic resonance in the five-membered ring (Figures S40–42). The obtained **CN-C2C2** was stable at low temperatures (220 K). However, when the temperature was raised, **CN-C2C2** gradually isomerized to **CN-OMe** bonded at N1 and C2’ (Figure S38). Thus, the selectivity of the radical reaction is temperature dependent; at low temperatures, the homodimerization of **CN-TAIR** to give **CN-C2C2** is kinetically favored, whereas at room temperature, the thermodynamically more stable **CN-OMe** is produced. The selective formation of radical coupling product **CN-C2C2** at low temperatures and the selective formation of **CN-OMe** at room temperatures cannot be fully explained by electrostatic interactions between the two radicals, i.e., the electrophilic radical **CN-TAIR** and the nucleophilic radical **OMe-TAIR**.

### Thermal Stability of TAIR and HABI

To investigate the effect of substituents on the stability of TAIR, the enthalpy Δ*H*°(Δ*H*_product_^°^– Δ*H*_reactant_^°^) of the isodesmic reaction^55,56^ was calculated using DFT at the B3LYP-D3/6-31G(d) level^54,57,58^ (Table 1). The introduction of a CN group destabilized the reaction enthalpy by approximately +0.3 kcal mol^–1^ (Table 1, entry1), whereas the introduction of a OMe group stabilized it by approximately −1.6 kcal mol^–1^ (Table 1, entry8). From the results for the other substituents, it was found that EWGs destabilized TAIR, whereas EDGs tended to stabilize TAIR. This effect of the substituent on the radical is different from the effect of the substituent on the stability of the benzyl radical,^59^ which was observed to be stabilized by both EWG and EDG substituents, but can be attributed to spin-resonance in the ring of TAIR. Comparing the spin densities in the five-membered ring, TAIR with a stronger EDG tended to have a lower spin density of the atoms N1 (N3), C2, and C4 (C5) in the five-membered ring. Conversely, the spin density increased with a stronger EWD. The effective delocalization of spins outside the five-membered ring is inferred to be due to the captodative (push–pull) effect^60,61^ between the EDG and electrophilic **TAIR**. The spin density of **TAIR** is preferentially localized on C2 due to the resonance effect and planarity of the aryl group at the C2 position. The spin on C2 has increased electrophilicity due to the electronegativity of the two N atoms, allowing for effective spin delocalization by the aryl group with an electron-donating substituent attached to C2. Thus, **TAIR** with an electron-donating substituent, such as an OMe group, is stabilized by delocalizing the spin through the push–pull effect.

**Table 1:**
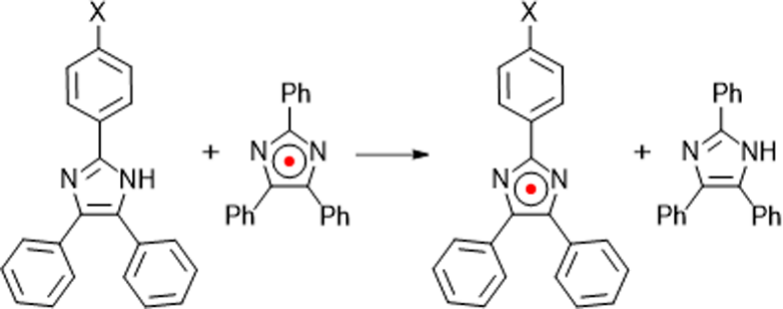
Energy Diagram of C–C Radical Coupling in **CN-C2C2**, **OMe-C2C2**, and **CN-OMe-C2C2**^a^

entry	X substituent	Δ*H*° (kcal mol^–1^)	spin density on N1	spin density on C2	spin density on C4
1	CN	+0.27	–0.077	+0.322	+0.260
2	CF_3_	+0.38	–0.075	+0.323	+0.258
3	COOMe	+0.68	–0.073	+0.320	+0.263
4	Cl	–0.34	–0.069	+0.313	+0.259
5	F	–0.72	–0.066	+0.308	+0.261
6	H	0.00	–0.068	+0.314	+0.259
7	Me	–0.41	–0.064	+0.306	+0.257
8	OMe	–1.58	–0.057	+0.288	+0.250
9	NH_2_	–2.65	–0.041	+0.254	+0.228
10	NMe_2_	–2.98	–0.043	+0.258	+0.232

a Data were calculated at the B3LYP-D3/6-31G(d) level of theory.

**Figure 6:**
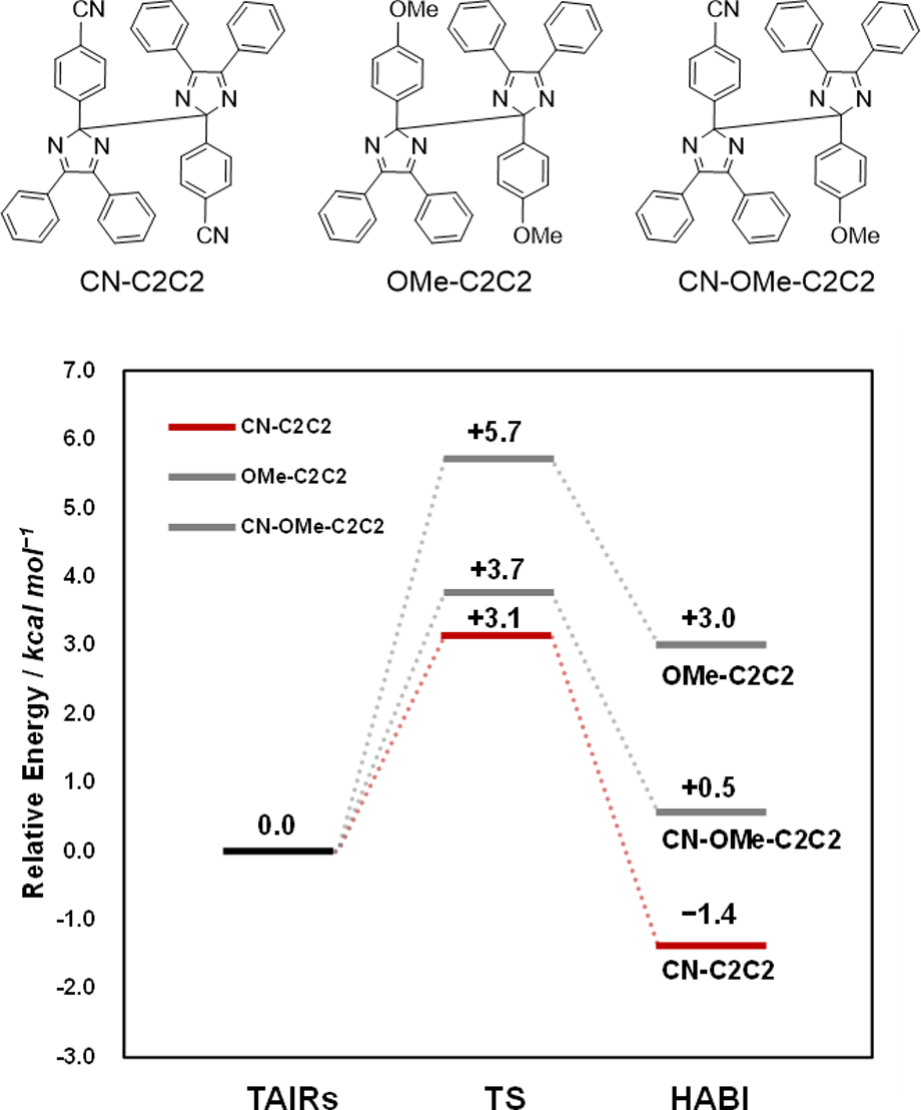
Energy diagram of C–C radical coupling in **CN-C2C2**, **OMe-C2C2**, and **CN-OMe-C2C2**. Data were simulated by DFT at the B3LYP-D3/6-31G(d) level.

These calculation results suggest that the homocoupling reaction between the more unstable and highly reactive radical **CN-TAIR** is expected to be kinetically fast. Therefore, the Gibbs energy of activation (Δ*G*^‡^) of each **C2C2** generation was simulated by DFT calculation (B3LYP-D3/6-31G(d)) (Figure 6). Here, the sum of the energies of the two **TAIR** molecules was set as the reference (0.0), and the relative energy was calculated. The Δ*G*^‡^ of **CN-C2C2** is smaller than that of **OMe-C2C2** and **CN-OMe-C2C2**, and the reaction is exothermic, which well represents the experimental results at low temperatures. These calculation results indicate that the thermodynamic stability of **TAIR** affects the selectivity in carbon–carbon radical coupling.

**Figure 7:**
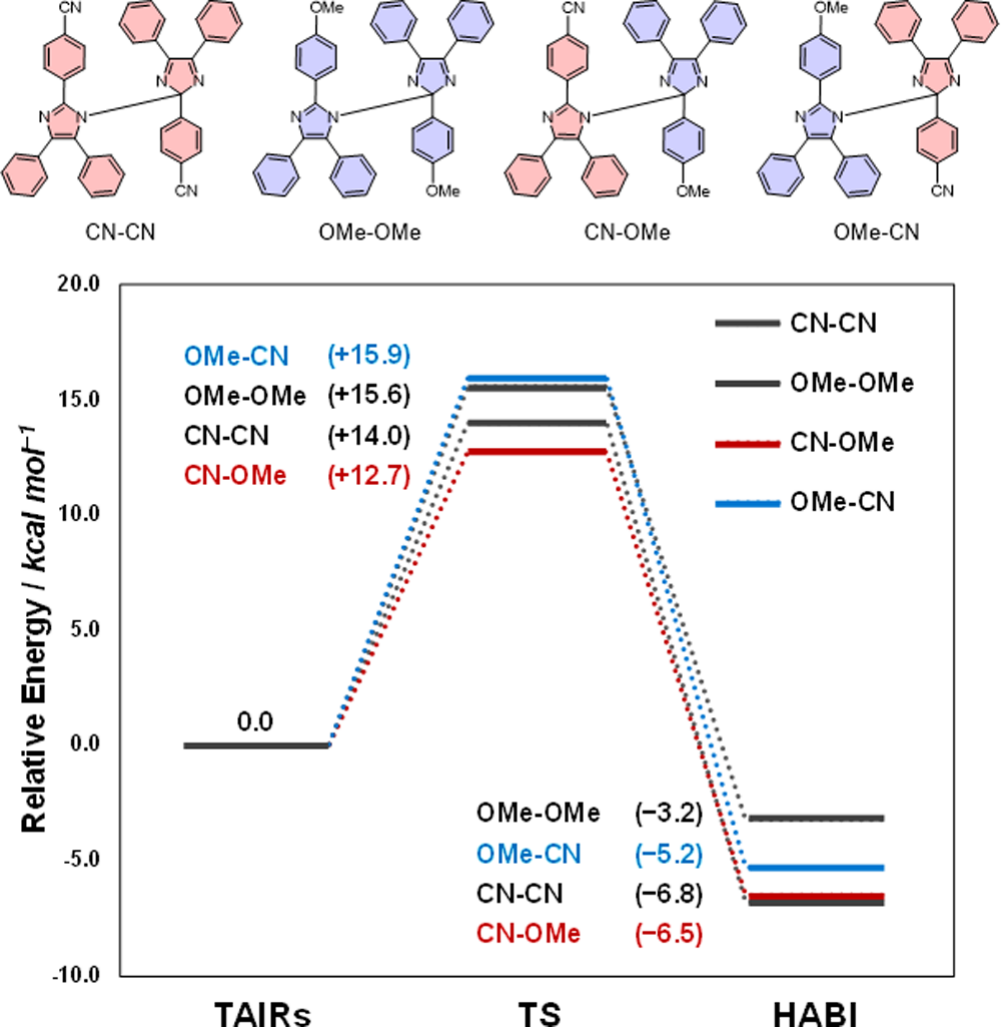
Energy diagram for radical recombination of homo- and hetero-HABI (**CN-CN**, **OMe-OMe**, **CN-OMe**, and **OMe-CN**). Resonance (R) and induced (I) effects of HABI derivatives. Data were simulated by DFT at the B3LYP-D3/6-31G(d) level.

The Gibbs energies of activation (Δ*G*^‡^) for the N1–C2’ radical coupling of the HABI derivatives, **CN-CN**, **OMe-OMe**, **CN-OMe**, and **OMe-CN,** were compared (Figure 7). The Δ*G*^‡^ (=14.0 kcal mol^–1^) for the formation of **CN-CN** in the coupling reaction of the homogeneous radical was 1.6 kcal mol^–1^ lower than that of **OMe-OMe** (Δ*G*^‡^ = 15.6 kcal mol^–1^), and the reaction energy was 3.6 kcal mol^–1^ higher. This is consistent with the experimental NMR results, which show that the dimerization of **CN-TAIR** is faster than that of **OMe-TAIR**. In addition, the calculated Δ*G*^‡^ for **TAIR** formation from the **HABI** derivatives **CN-OMe** and **OMe-CN** in the radical coupling reaction of **CN-TAIR** and **OMe-TAIR** was 12.7 and 15.9 kcal mol^–1^, respectively, consistent with the experimental observation that **CN-OMe** is selectively formed. Interestingly, the difference in the reaction energy of **CN-OMe** versus that of **OMe-CN** was smaller than the difference in Δ*G*^‡^ (3.2 kcal mol^–1^), where the reaction energy of **CN-OMe** was only 1.3 kcal mol^–1^ larger. This negates the selectivity due to radical stability seen in carbon–carbon coupling and suggests specific stabilization of the transition state in the N1–C2’ coupling reaction to produce **CN-OMe**.

### Impact of Transition-State Aromaticity

**Figure 8:**
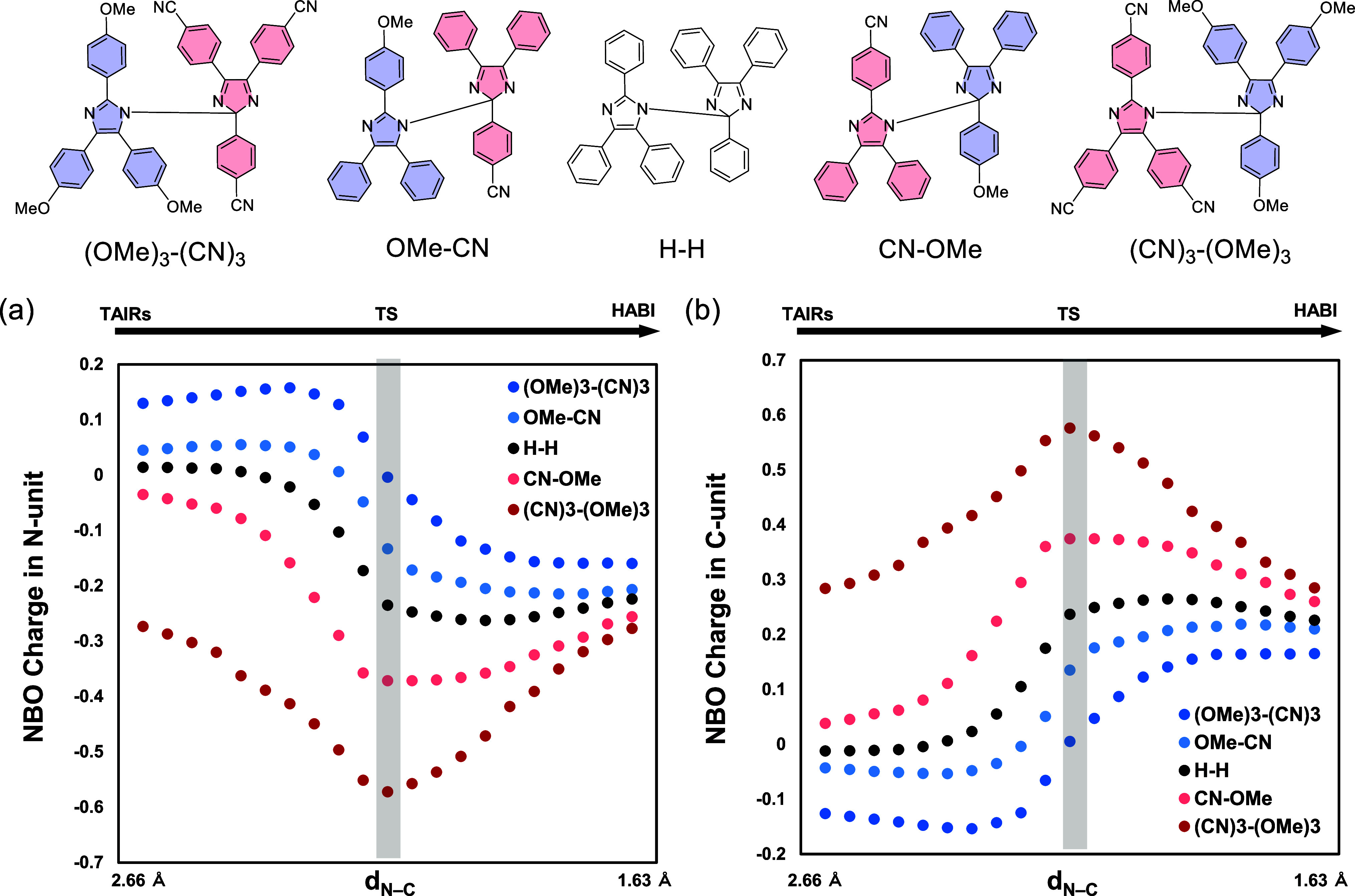
(a) NBO charge for the N-side and (b) C-side TAIR unit in each conformation of HABI derivatives (**(OMe)**_**3**_**-(CN)**_**3**_, **OMe-CN**, **H–H**, **CN-CN**, and **(CN)**_**3**_**-(OMe)**_**3**_).

To understand the selective formation of **CN-OMe**, the charge distribution during the radical coupling process was investigated by plotting the NBO charge of each structure obtained by the intrinsic reaction coordinate (IRC) calculation of the transition state (TS) (Figure 8). In addition to the combination of **CN-TAIR** and **OMe-TAIR** investigated in the experiment, the combination of **(CN)**_**3**_**-TAIR** and **(OMe)**_**3**_**-TAIR**, in which the electronic effect is thought to be more pronounced, was also examined, and these results were compared to those of **H–H**, a dimer of two**H-TAIR**, used as a standard compound. First, the charge distributions at the **CN-TAIR** and **OMe-TAIR** moieties during the reaction were plotted (Figure 8a,b) (note that **CN-OMe** was selectively obtained in the experiment). The results show that the charge on the **CN-TAIR** moiety became negative as the radical coupling reaction proceeded. Conversely, it was revealed that the charge on the **OMe-TAIR** moiety became positive. In other words, when a bond begins to form between N1 of the **CN-TAIR** site and C2 of the **OMe-TAIR** moiety, charge transfer occurs from the **OMe-TAIR** moiety to the **CN-TAIR** moiety. To confirm this phenomenon, we also examined the combination of **(CN)**_**3**_**-TAIR** and **(OMe)**_**3**_**-TAIR** in the same way. The charge distribution plot shows more significant charge transfer than with the combination of **CN-TAIR** and **OMe-TAIR**. Conversely, in the reaction to form **OMe-CN**, the charge at the **OMe-TAIR** moiety that reacts with the nitrogen becomes positive as it approaches the transition state.

**Figure 9:**
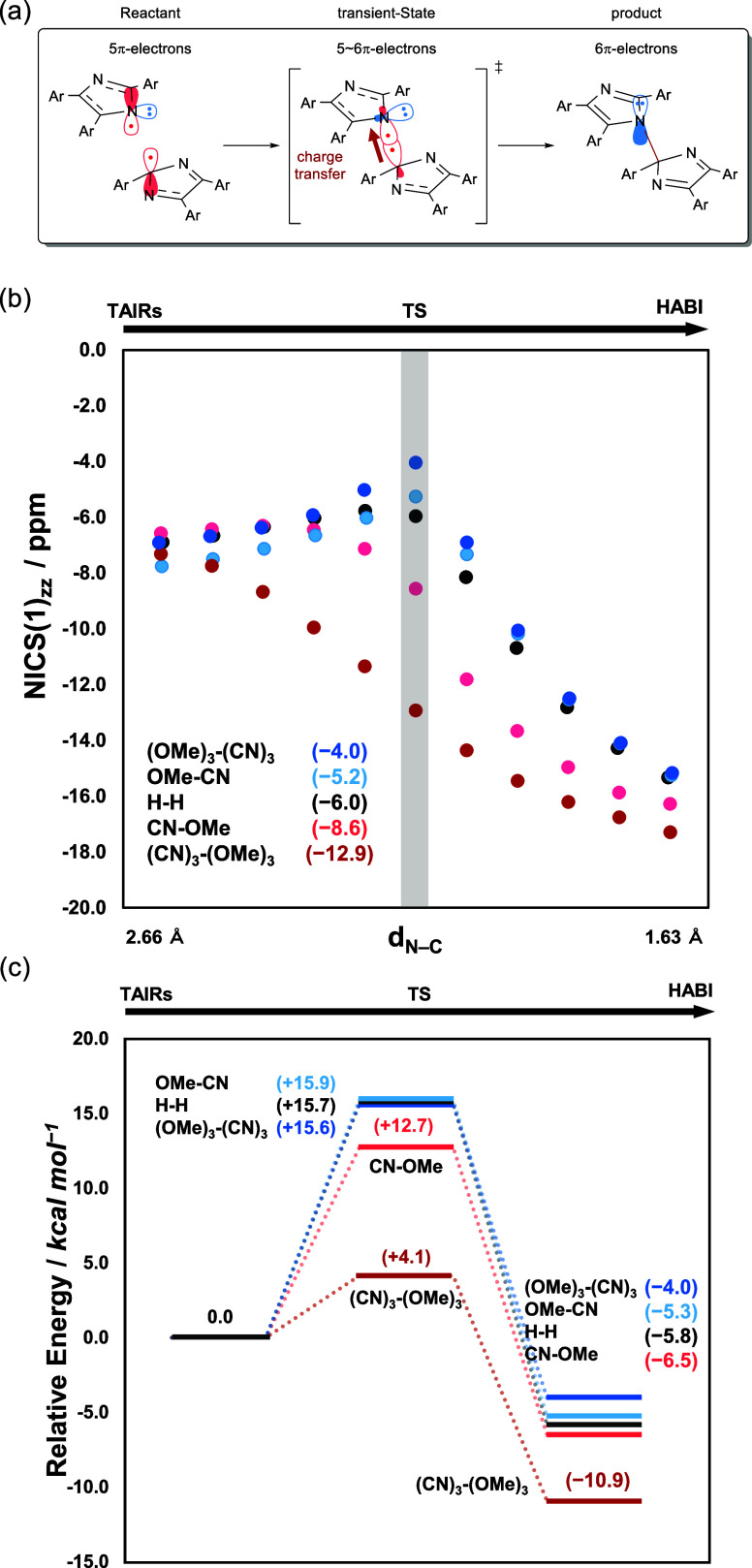
(a) Transition of the electronic system from the reactant (TAIR) to the product (HABI) through the TS. (b) NICS(1)_*zz*_ value of HABI derivatives (**(OMe)**_**3**_**-(CN)**_**3**_, **OMe-CN**, **H–H**, **CN-OMe**, and **(CN)**_**3**_**-(OMe)**_**3**_) near the transition state. (c) Energy diagram of the radical–radical coupling reaction, while the **CN-TAIR** moiety that reacts with C2 becomes negative, and the charge difference was even more significant for the combination of **(CN)**_**3**_**-TAIR** and **(OMe)**_**3**_**-TAIR**. In other words, the difference in the charge distribution can be rationally explained by the transfer of charge from the nucleophilic **OMe-TAIR** moiety to the electrophilic **CN-TAIR** moiety as a bond is formed.

As shown in Figure 9a, when the charge is transferred from the **OMe-TAIR** moiety to the **CN-TAIR** moiety, the number of π-electrons at the **CN-TAIR** moiety increases from 5, and it is thought that aromaticity is developed at the **CN-TAIR** site. Therefore, NICS(1)_*zz*_ calculations in the N-unit and C-unit were performed for each structure obtained by IRC calculation. The results show that the NICS value of the N-unit (Figure 9b) becomes significantly negative as the system approaches the transition state (TS). On the other hand, the NICS value of the C-unit (Figure S45), ∼ −6–7, does not change significantly during the radical–radical coupling reaction. In the reaction to generate **OMe-CN**, the NICS value of the N-unit becomes positive, and after reaching a maximum, a final product with aromaticity is obtained. This change in the NICS value was more significant in the reactions of **(CN)**_**3**_**-TAIR** and **(OMe)**_**3**_**-TAIR**. In other words, the NICS value for the TS that forms **(CN)**_**3**_**-(OMe)**_**3**_ was −12.9 ppm (Figure 9b), which is a larger negative change than that of **(CN)**_**3**_**-TAIR** (−7.5 ppm). On the other hand, the NICS value of the TS that forms **(OMe)**_**3**_**-(CN)**_**3**_ was approximately −4.0 ppm, a large positive change from −6.6 ppm (Figure 9b). This suggests that when the TAIR unit reacting at the N is an electrophilic radical, the aromaticity of the TS becomes greater, resulting in greater stability. Indeed, the energy of TSs that form **CN-OMe** and **(CN)**_**3**_**-(OMe)**_**3**_ is 11.5 and 3.2 kcal mol^–1^ lower than the energy of the transition states that form **OMe-CN** and **(OMe)**_**3**_**-(CN)**_**3**_, respectively, which is in good agreement with the selective production of **CN-OMe** observed in the experiment (Figure 9c).

The orbital interactions were simulated using the NBO second-order perturbation theory (Table 2).^62^ Here, we evaluated which of the lone pairs (LPs) and σ-bonds contribute more to the π*-orbitals of the N-units (π_a_* [N=C antibonding] and π_b_* [C=C antibonding]). The energy for donation of the LP to π* is approximately 2.0–3.0 kcal mol^–1^, whereas the σ- donation energy is 7.0–14.0 kcal mol^–1^, which is significantly higher than that of LP. This means that the σ-bond orbitals are mainly involved in π-conjugation in the TS. The energy gaps of **(CN)**_**3**_**-(OMe)**_**3**_ are the highest at 13.76 and 9.41 kcal mol^–1^ (entry 5), whereas those of **(OMe)**_**3**_**-(CN)**_**3**_ are the lowest at 9.09 and 7.54 kcal mol^–1^. This difference comes from the polarization in the TS due to the substituent effect of the CN and OMe groups. This strong σ-bond donation is thought to be due to the small angle of the σ-bond with the imidazole plane (∼107°) in the transition state, accounting for the greater resonance stabilization. There have been few experimental reports on the involvement of σ-bonds in aromaticity,^63−71^ and this study is the first example in which reaction control is achieved using mixed σ-bond TS aromaticity. The NICS values, NBO charges, and second-order perturbation theory revealed that TS aromaticity is an important factor in selective radical coupling. TS aromaticity can be effectively strengthened by the electronegativity of N and polarization due to the substituent effect.

**Table 2:**
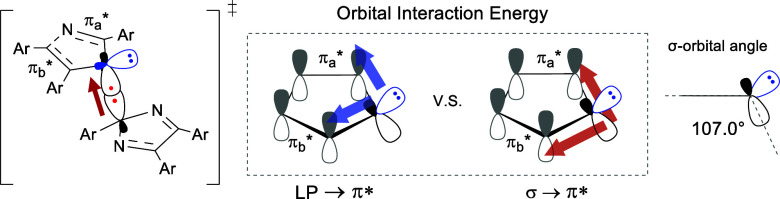
Orbital Interaction Simulated Using the 2nd-Order Perturbation Theory in NBO

entry	compound	Δ*E*_AB_(2) LP → π_a_^*^	Δ*E*_AB_(2) LP → π_b_^*^	Δ*E*_AB_(2) σ → π_a_^*^	Δ*E*_AB_(2) σ → π_b_^*^
1	**(OMe)**_**3**_**-(CN)**_**3**_	2.50	2.30	9.09	7.54
2	**OMe-CN**	2.76	2.27	11.60	8.84
3	**H–H**	2.88	2.30	12.73	9.21
4	**CN-OMe**	2.92	2.02	13.09	9.19
5	**(CN)**_**3**_**-(OMe)**_**3**_	3.05	2.15	13.76	9.41

### Hammett Plot

To investigate the effect of TS aromaticity caused by substituents on radical–radical cross-coupling reactions, Hammett plots were conducted for the formation of HABI derivatives **X-OMe** and **CN–X** (Table 3).^72,73^ Each reaction rate constant (*k*) was determined from the Gibbs energy of activation calculated at 298 K (Figure S48), and the logarithm of the relative ratio based on *k*_H_ (rate constant for X = H) was taken. For HABI-derivative **X-OMe**, the Hammett plot showed a positive slope and a good linear relationship was obtained. Thus, the EWG in X accelerate the radical–radical coupling reaction. In contrast, for derivative **CN–X**, the Hammett plot had a negative slope, indicating that EDG accelerates the coupling reaction. As mentioned above, TAIR is stabilized by EDGs; thus, this result is contrary to the tendency of thermodynamic stabilization of TAIR, and the negative slope of the Hammett plot cannot be explained only by the stability of the radical. In other words, it is thought that the TS aromaticity increased by introducing an EDG on to X and the reaction proceeded at a faster rate.

**Table 3:**
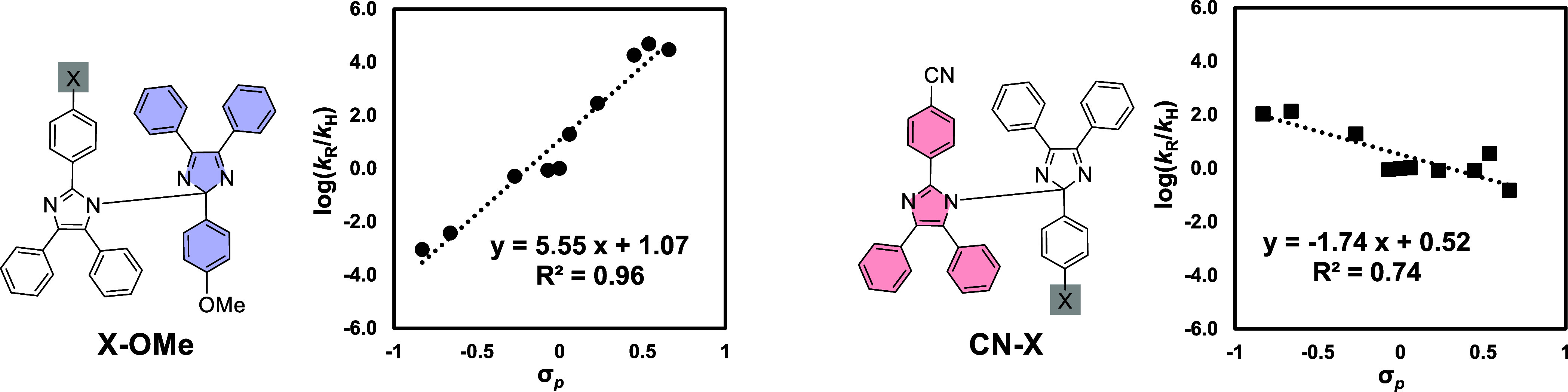
Hammett Plot for the Formation of HABI-Derivative **X-OMe** and **CN–X** in the Radical–Radical Coupling Reaction

			X–OMe				CN–X			
entry	X substituent (σ_p_)^a^	Δ*H*° (kcal mol^–1^)^b^	Δ^‡^*G*^○^ (kcal mol^–1^)^c^	*k*_R_^d^	*k*_R_/*k*_H_	NICS(1)_zz_	Δ^‡^*G*^○^ (kcal/mol)^c^	*k*_R_^d^	*k*_R_/*k*_H_	NICS(1)_zz_
1	CN (0.66)	+0.27	12.7	2858.1	4.5	–12.0	14.0	343.7	–0.8	–11.0
2	CF_3_ (0.54)	+0.38	12.6	3532.4	4.7	–12.8	13.1	1683.1	0.8	–11.7
3	COOMe (0.45)	+0.68	12.9	2312.5	4.3	–13.1	13.6	721.3	–0.1	–11.5
4	Cl (0.23)	–0.34	13.9	382.1	2.5	–12.4	13.6	721.3	–0.1	–11.3
5	F (0.06)	–0.72	14.6	119.2	1.3	–11.4	13.5	801.9	0.0	–11.3
6	H (0.00)	0.00	15.4	32.7	0.0^e^	–10.5	13.5	785.1	0.0^e^	–11.0
7	Me (−0.07)	–0.41	14.4	184.0	1.7	–12.3	12.5	4412.3	1.7	–12.0
8	OMe (−0.27)	–1.58	15.6	24.3	–0.3	–11.2	12.7	2858.1	1.3	–12.5
9	NH_2_ (−0.66)	–2.65	16.8	2.9	–2.4	–11.7	12.2	6598.5	2.1	–13.3
10	NMe_2_ (−0.83)	–2.98	17.2	1.6	–3.1	–9.5	12.3	5998.6	2.0	–14.6

a Hammett parameter (σ_p_).

b Radical stability was evaluated using the isodesmic reaction.

c Δ*G* was determined as the energy gap between TAIR and the TS by theoretical calculation.

d Rate constant (*k*) is defined in eq 3 (Figure S48).

e *k*_R_/*k*_H_ represents the rate constant for each HABI derivative with X ≠ H divided by the rate constant for X = H.

## Conclusions

Although the selectivity of radical–radical coupling reactions is usually low, in the radical cross-coupling of **CN-TAIR** and **OMe-TAIR**, **CN-OMe** was selectively bound to N1 of **CN-TAIR** and C2 of **OMe-TAIR** and was isolated with high selectivity from 24 possible isomers; the structure was determined by X-ray analysis. The rate constant of the cross recombination was faster than that of homodimerization to form **CN-CN** and **OMe-OMe**, and it was revealed that **CN-TAIR** and **OMe-TAIR** reacted selectively. When heteroradicals of **CN-TAIR** and **OMe-TAIR** were generated at low temperature such as 220 K, the isomer **CN-C2C2** with a C–C bond was selectively obtained by homodimerization of **CN-TAIR**. Experimentally, it was revealed that **CN-TAIR** and **OMe-TAIR** did not react selectively at low temperature and that the interaction between electrophilic and nucleophilic radicals alone does not contribute to the heteroradical coupling to produce **CN-OMe**.

The reactivity of **TAIR** was predicted by using theoretical calculations. As observed experimentally, the activation energy of **CN-OMe** is smaller than that of **OMe-CN**, in which the bond formation center is reversed, even though both reactions involve a cross-coupling reaction between the same **TAIR** molecules. This selectivity cannot be explained by the effect of the thermodynamic stability of the radicals. Therefore, the bond polarization-induced aromaticity in the TS structure was evaluated from the NICS value. The NICS value of the transition state giving the **CN-OMe** structure was more negative than that of the transition state giving the **OMe-CN** structure, and it was revealed that the transition state giving the **CN-OMe** structure is stabilized by the transition-state aromaticity. This study reveals, for the first time, that cross-radical coupling can be performed regioselectively with high yield and provides interesting fundamental knowledge about TS-aromaticity in radical coupling reactions.

## Supporting Information Available

Materials and methods, synthetic procedures, characterization of compounds, detailed experimental procedures, calculation data, and spectroscopic data (PDF)
