# Supplementary material for: Impact of Transition-State Aromaticity on Selective Radical–Radical Coupling of Triarylimidazolyl Radicals
Source: J Am Chem Soc. 2024 Nov 28;147(3):2559–70. doi: 10.1021/jacs.4c14095 (PMC11760180; doi:10.1021/jacs.4c14095)
Supplement: Supplementary file 1 — ja4c14095_si_001.pdf [file ja4c14095_si_001.pdf]

Supporting Information

Impact of Transition-State Aromaticity on Selective Radical-Radical Coupling of Triarylimidazolyl Radicals

Kazunori Okamoto, Sayaka Hatano and Manabu Abe*

Department of Chemistry, Graduate School of Advanced Science and Engineering, Hiroshima University, Higashi-Hiroshima 739-8526, Japan, *mabe@hiroshima-u.ac.jp

Table of Contents

1. General Information S2-S3
2. Synthesis and Spectral Data S4-S15
3. UV-vis Absorption Spectra and Photo Stability S16
4. Single X-ray Structure Analysis Data S17-19
5. Rate constant for Radical-Radical Coupling S20-S21
6. Photoreaction for Each HABI Derivative S22-S26
7. Photoreaction at Low Temperature S27-S30
8. Simulated NMR Data S31-S32
9. Isodesmic Reaction S33
10. Transient-State and Energy Diagram S34-S35
11. NICS Computation S36-37
12. NBO Charge S38-39
13. Hammett Plot S40-S41
14. Theoretical Calculations S41-S61
15. Reference S62

1. General Information

Reagents and Solvents

Materials obtained from commercial suppliers were used as received. Otherwise noted, all reactions were performed with dry solvents under an atmosphere of nitrogen gas in dried glassware.

Procedure

All workup and purification procedures were carried out with reagent-grade solvents in the air. Thin-layer chromatography (TLC) analyses were performed on commercial aluminum sheets of Merck silica gel 60F254 and visualized with an ultraviolet lamp (λ = 254 nm). Purification was done by column chromatography using silica gel (63−210 μm).

^{1}H & ^{13}C NMR Spectroscopy

NMR spectra were recorded on a Bruker Ascend 400 to give ^{1}H NMR (400 MHz) spectra and ^{13}C NMR (100 MHz) spectra. Chemical shifts for ^{1}H NMR are expressed in parts per million (ppm) relative to tetramethylsilane (δ 0.00 ppm) or the residual peak of $CDCl_3$ (δ 7.26 ppm), C_6D_6 (δ 7.16 ppm) and referenced. Chemical shifts for ^{13}C NMR are expressed in ppm relative to $CDCl_3$ (δ 77.16 ppm). Data are reported as follows: chemical shift, multiplicity (s = singlet, dt = doublet of triplet, tt = triplet of triplet, m = multiplet), coupling constant (Hz), and integration.

Mass Spectroscopy

Mass-spectrometric data were measured with a mass spectrometric Thermo Fisher Scientific LTQ Orbitrap XL.

Single-Crystal X-ray Diffraction Analysis.

The diffraction data of the single crystals were collected on a Bruker APEX-II Ultra CCD diffractometer and Rigaku XtaLAB Synergy R, DW. The structures were solved using direct methods and refined full-matrix least-squares techniques using the SHELX program package. All non-hydrogen atoms were anisotropically refined. Single crystal X-ray structure was visualized by ORTEP.

UV−Vis Absorption Spectroscopy.

UV−vis absorption spectra were recorded on a SHIMADZU UV-3600 Plus spectrophotometer. All samples were measured in spectrograde solvents (CH_2Cl_2, benzene) with a 10 mm × 10 mm quartz cell.

EPR Spectroscopy.

EPR spectra were recorded on a Bruker BioSpin Elexsys E500. A solution of tBu-1,4′-HABI in toluene was transferred to a quartz EPR tube and degassed under a high vacuum. The EPR tube was sealed under the vacuum conditions (???? Pa) after three freeze−pump−thaw cycles.

Light Source.

All photoreaction of tBu-1,4′-HABI was carried out using a 365 nm LED apparatus (CL-1501, Asahi Spectra) equipped with LED head unit (CL-H1-365-9-1) for generating tBu-TPIR.

DFT Calculations.

DFT calculations were carried out in the gas phase using the Gaussian 16 (Revision C.02) program package. All calculations were performed in the gas phase. The energy profile for each HABI derivative was calculated at the B3LYP-D3/ 6-31G(d) level of theory. In addition, intrinsic reaction coordinate (IRC) calculations were performed to verify that the transition state is associated with the reactant and the expected product.

2. Synthesis and Spectral Data [S1-2]

Synthesis of CN-TAI

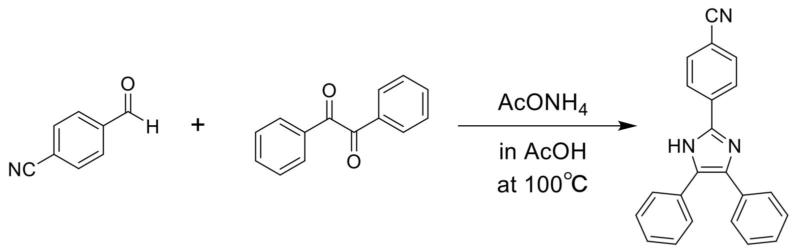

1-CN **2** **CN-TAI**

The solution of 1-CN (4-cyanobenzoaldehyde, 500 mg, 3.8 mmol), 2 (benzil, 802 mg, 3.9 mmol) and ammonium acetate (2.9 g, 37.6 mmol) was stirred in AcOH at 100 °C for 12 h (overnight). After cooling to room temperature, the reaction mixture was neutralized by adding saturated K_2CO_3 *aq*. dropwise in an ice bath, resulting in the formation of a white precipitate. The mixture was extracted three times with CH_2Cl_2. The combined organic layer was dried over $MgSO_4$. After filtration, the solution was concentrated under in vacuo to obtain a white solid. Recrystallization from hot acetonitrile at ~80 °C afforded white needle crystals of CN-TAI (1.2 g, 37.3 mmol, 95 %). ^{1}H NMR (400 MHz, $CDCl_3$): δ 9.56 (br 1H), 8.04-8.02 (dt, *J* = 8.4 and 1.8 Hz, 2H), 7.74-7.71 (dt, J = 8.7 and 1.3 Hz, 2H), 7.65−7.64 (d, J = 7.3, 2H), 7.48-7.47 (m, 2H), 7.43-7.38 (m, 3H), 7.36-7.27 (m, 3H). ^{13}C{1 H} NMR (100 MHz, $CDCl_3$) δ 143.7, 133.8, 132.8, 129.1, 128.5, 128.1, 128.0, 127.7, 127.4, 125.4, 125.2, 118.7, 111.8.

Synthesis of OMe-TAI

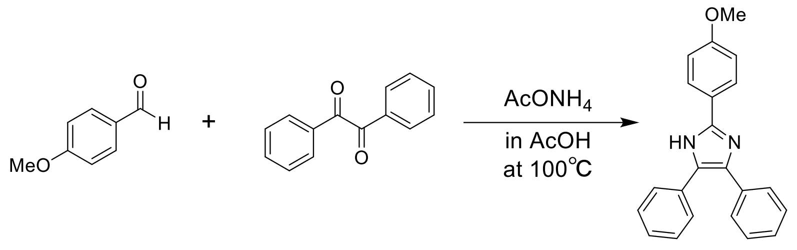

1-OMe **2** **OMe-TAI**

The solution of 1-OMe (4-methoxybenzoaldehyde, 510 mg, 3.7 mmol), 2 (benzil, 787.5 mg, 3.7 mmol) and ammonium acetate (2.9 g, 37.5 mmol) was stirred in AcOH at 100 °C for 12 h (overnight). After cooling to room temperature, the reaction mixture was neutralized by adding saturated K_2CO_3 *aq*. dropwise in an ice bath, resulting in the formation of a white precipitate. The mixture was extracted three times with CH_2Cl_2. The combined organic layer was dried over $MgSO_4$. After filtration, the solution was concentrated under in vacuo to obtain a white solid. Recrystallization from hot acetonitrile at ~80 °C afforded white needle crystals of OMe-TAI (1.2 g, 37.5 mmol, 97 %). ^{1}H NMR (400 MHz, $CDCl_3$): 7.86-7.83 (dt, *J* = 8.9 and 2.2 Hz, 2H), 7.56 (Br, 3H), 7.36−7.33 (t, J = 7.2, 4H), 7.30-7.29 (d, 6.8 2H), 6.99-6.97 (dt, J = 8.9 and 2.5 Hz, 2H). ^{13}C NMR (100 MHz, $CDCl_3$) δ 160.3, 146.0, 128.6, 127.8, 126.7, 122.8, 114.3, 55.4.

Synthesis of CN-CN

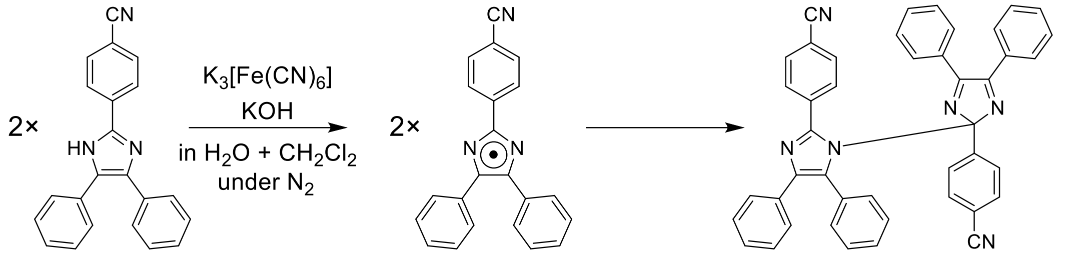

CN-TAI **CN-TAIR** **CN-CN**

The solution-1 of potassium ferricyanide ($K_3[Fe(CN)_6]$, 523.8 mg, 1.6 mmol) and KOH (87.6 mg, 1.6 mmol) in degassed H_2O (10 ml) was frozen at −77 K in the liquid N_2. The solution-2 of CN-TAI (2-(4-cyanophenyl)-4,5-diphenyl-1*H*-imidazole, 20.0 mg, 0.03 mmol) in degassed CH_2Cl_2 (10 ml) was added to the solution-1 under keeping temperature. The oxygen was removed from the solution by freeze-pump-thaw in three times. The two-layer solution was vigorously stirred for 10 h at room temperature under the dark condition. The organic layer was extracted by CH_2Cl_2 (3 × 10 mL), and washed with water (3 × 20 mL). The combined organic layer was dried over $MgSO_4$, filtered, and the solution was concentrated under in vacuo to obtain a pale yellow solid, CN-CN (39.8 mg, 0.06 mmol), quantitatively. The single crystal was prepared in the mixing solution (EtOAc : Hex = 5 : 1), in the test tubes, under the dark condition, at 5 °C.

^{1}H NMR (400 MHz, $CDCl_3$): δ 7.64 (d, J = 8.0 Hz, 2H), 7.54 (t, J = 7.4 Hz, 2H), 7.45 (d, J = 7.8, J = 7.9 Hz, 6H), 7.28-7.25 (m, 9H), 7.23-7.14 (m, 7H). ^{13}C NMR (100 MHz, $CDCl_3$) δ 167,93, 142.47, 132.11, 132.06, 131.39, 131.31, 130.83, 130.36, 129.29, 128.38, 128.35, 128.17, 128.06, 127.10, 118.30, 118.08, 112.47, 111.38. FTMS (p-ESI, MeOH sol.) m/z: Calcd for $C_{44}H_{29}N_6$ [M + H]$^+$, 641.24482, found 641.24493.

Synthesis of OMe-OMe

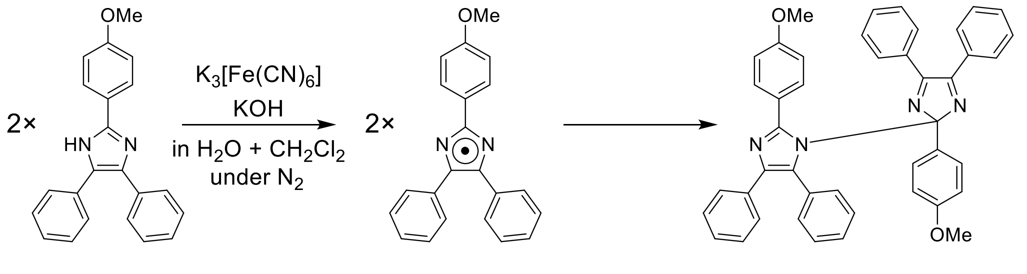

OMe-TAI **OMe-TAIR** **OMe-OMe**

The solution-1 of potassium ferricyanide ($K_3[Fe(CN)_6]$, 505.9 mg, 1.5 mmol) and KOH (86.2 mg, 1.6 mmol) in degassed H_2O (10 ml) was frozen at −77 K in the liquid N_2. The solution-2 of OMe-TAI (2-(4-methoxyphenyl)-4,5-diphenyl-1*H*-imidazole, 20.0 mg, 0.03 mmol) in degassed CH_2Cl_2 (10 ml) was add to the solution-1 under keeping temperature. The oxygen was removed from the solution by freeze-pump-thaw in three times. The two layers solution was vigorously stirred for 10 h at room temperature under the dark condition. The organic layer was extracted by CH_2Cl_2 (3 × 10 mL), and washed with water (3 × 20 mL). The combined organic layer was dried over $MgSO_4$, filtered, and the solution was concentrated under in vacuo to obtain a pale yellow solid, OMe-OMe (39.9 mg, 0.06 mmol), quantitatively. The single crystal was prepared in the mixing solution (EtOAc : Hex = 5 : 1), in the test tubes, under the dark condition, at 5 °C.

^{1}H NMR (400 MHz, $CDCl_3$): δ 7.44-7.42 (m, 4H), 7.35 (dd, J = 6.8 and 1.6 Hz, 2H), 7.31-7.24 (m, 9H), 7.16-7.06 (m, 9H), 6.72 (dt, J = 8.8 and 2.4 Hz, 2H), 6.44 (dt, J = 8.9 and 2.5 Hz, 2H), 3.74 (s, 3H), 3.67 (s, 3H). ^{13}C NMR (100 MHz, $CDCl_3$) δ 159.49, 159.45, 148.84, 137.78, 134.60, 133.12, 131.98, 131.52, 130.93, 130.40, 130.24, 129.37, 129.33, 127.98, 127.80, 127.73, 127.15, 127.10, 126.06, 113.00, 112.90, 112.22, 55.27. FTMS (p-ESI, MeOH sol.) m/z: Calcd for $C_{44}H_{34}N_4O_2$ [M + H]$^+$, 651.27545, found 651.27563.

Synthesis of CN-OMe

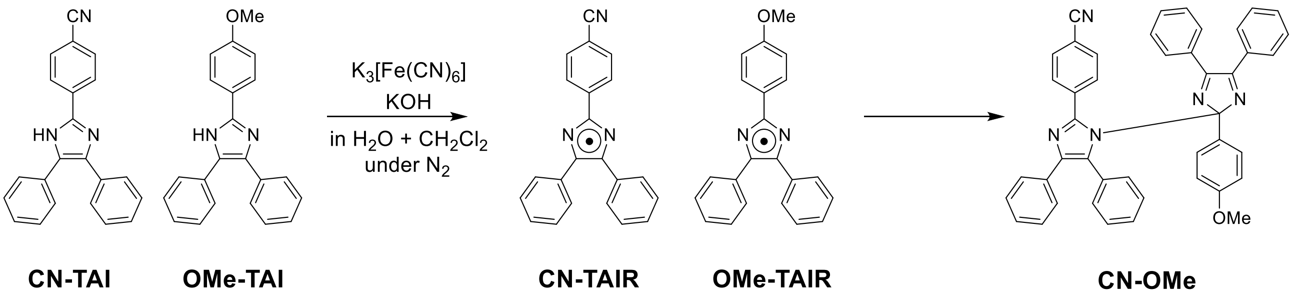

The solution-1 of potassium ferricyanide ($K_3[Fe(CN)_6]$, 505.9 mg, 1.5 mmol) and KOH (86.2 mg, 1.6 mmol) in degassed H_2O (10 ml) was frozen at −77 K in the liquid N_2. The solution-2 of CN-TAI (2-(4-cyanophenyl)-4,5-diphenyl-1*H*-imidazole, 10.0 mg, 0.02 mmol) and OMe-TAI (2-(4-methoxyphenyl)-4,5-diphenyl-1*H*-imidazole, 10.2 mg, 0.02 mmol) in degassed CH_2Cl_2 (10 ml) was add to the solution-1 under keeping temperature. The oxygen was removed from the solution by freeze-pump-thaw in three times. The two layers solution was vigorously stirred for 2 h at room temperature under the dark condition. The organic layer was extracted by CH_2Cl_2 (3 × 10 mL), and washed with water (3 × 20 mL). The combined organic layer was dried over $MgSO_4$, filtered, and the solution was concentrated under in vacuo to obtain a pale yellow solid. The crude mixture was quickly purified by silica gel column chromatography (Hexane : EtOAc = 2 : 1), then the solvent was removed under reduced pressure. The yellow solid was washed by MeCN to give the pale-yellow solid, CN-OMe (16.5 mg, 0.03 mmol) in 82 % yield. The single crystal was prepared in the mixing solution (EtOAc : Hex = 5 : 1), in the test tubes, under the dark condition, at 5 °C.

^1H NMR (400 MHz, $CDCl_3$): δ 7.68 (dt, *J* = 8.6 and 1.8 Hz, 2H), 7.50-7.43 (m, 4H), 7.34-7.24 (m, 12H), 7.18-7.07 (m, 8H), 6.46 (dt, J = 9.0 and 2.6 Hz, 2H), 6.44 (dt, J = 8.9 and 2.5 Hz, 2H), 3.68 (s, 3H). ^{13}C NMR (100 MHz, $CDCl_3$) δ 166.55, 159.83, 147.07, 139.04, 138.52, 134.05, 132.54, 132.10, 131.29, 131.13, 130.80, 129.81, 128.36, 128.01, 126.47, 118.82, 113.05, 112.12, 111.15, 55.32. FTMS (p-ESI, MeOH sol.) m/z: Calcd for $C_{44}H_{34}N_4O_2$ $[M + H]^+$, 646.26014, found 646.26093.

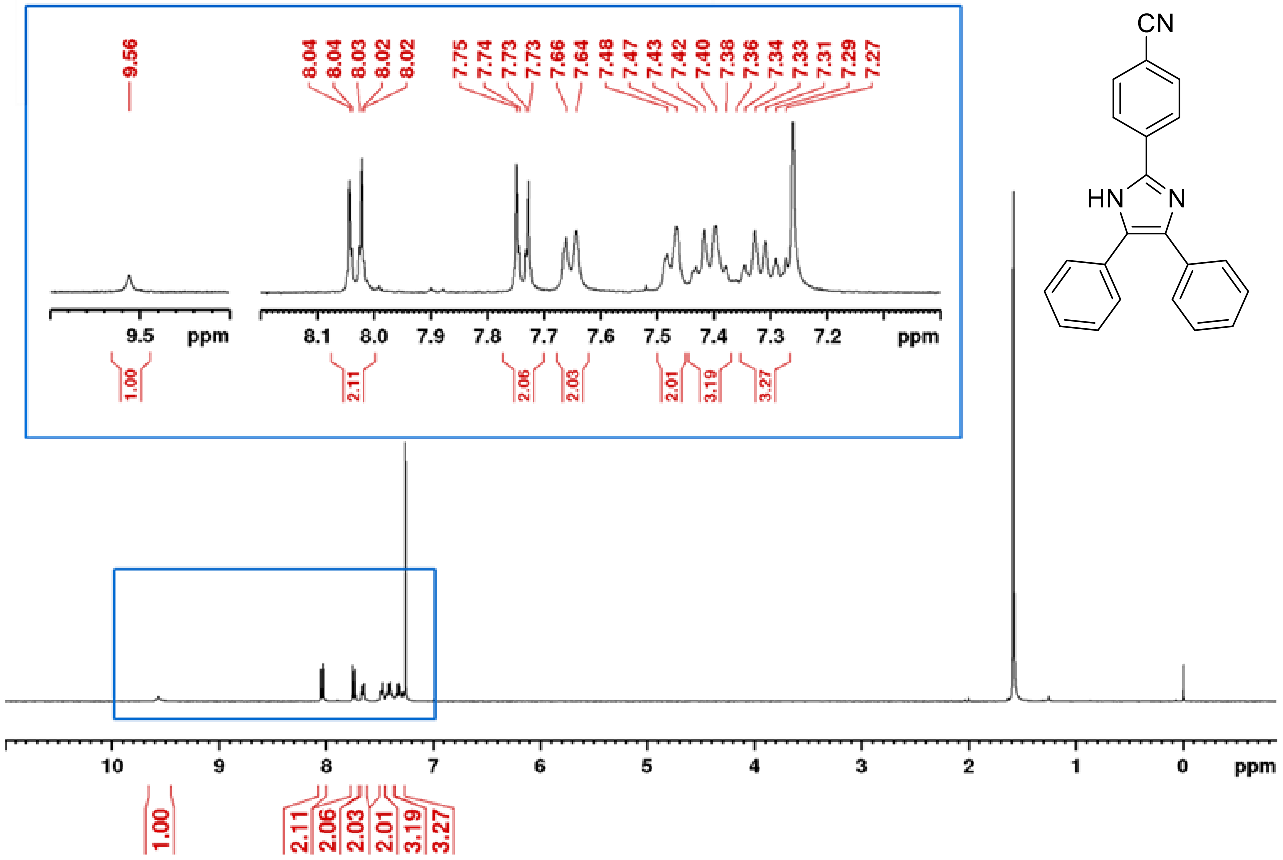

Figure S1. ^{1}H NMR spectrum of **CN-TAI**

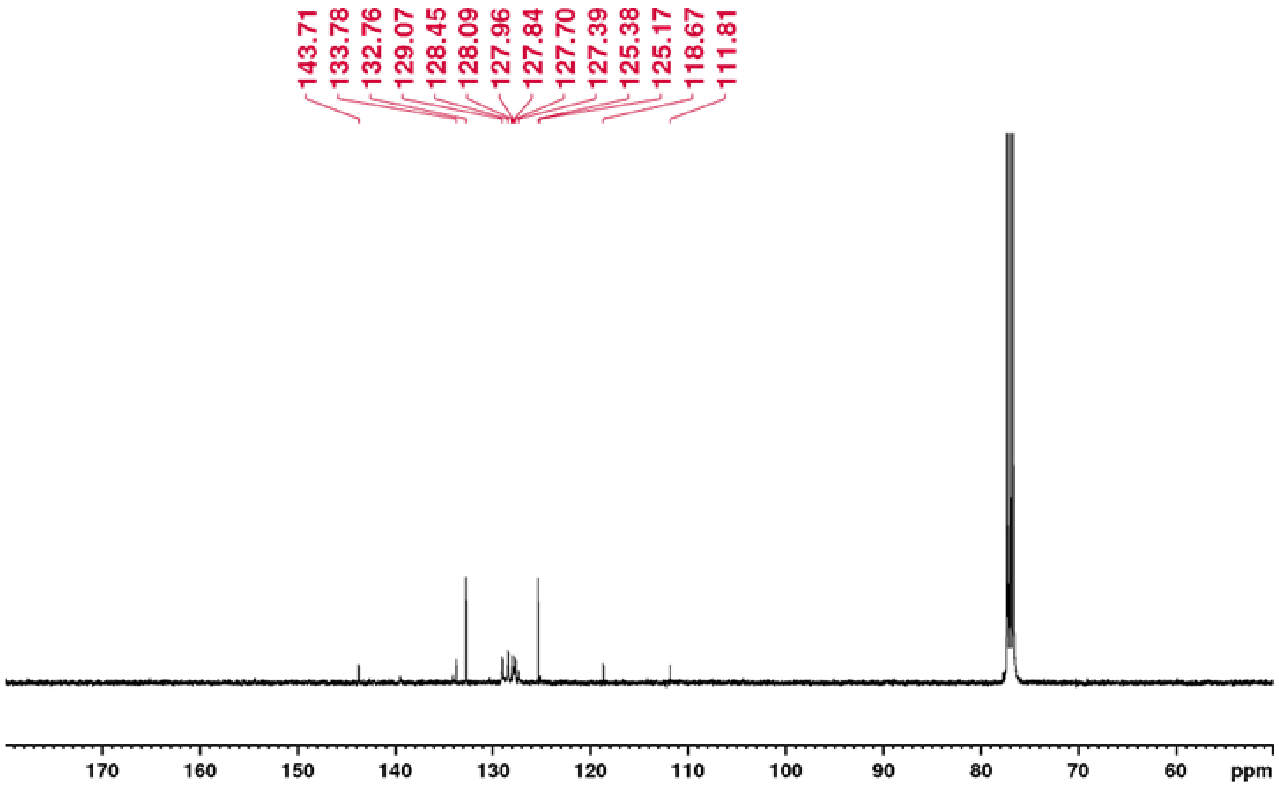

Figure S2. ^{13}C NMR spectrum of **CN-TAI**

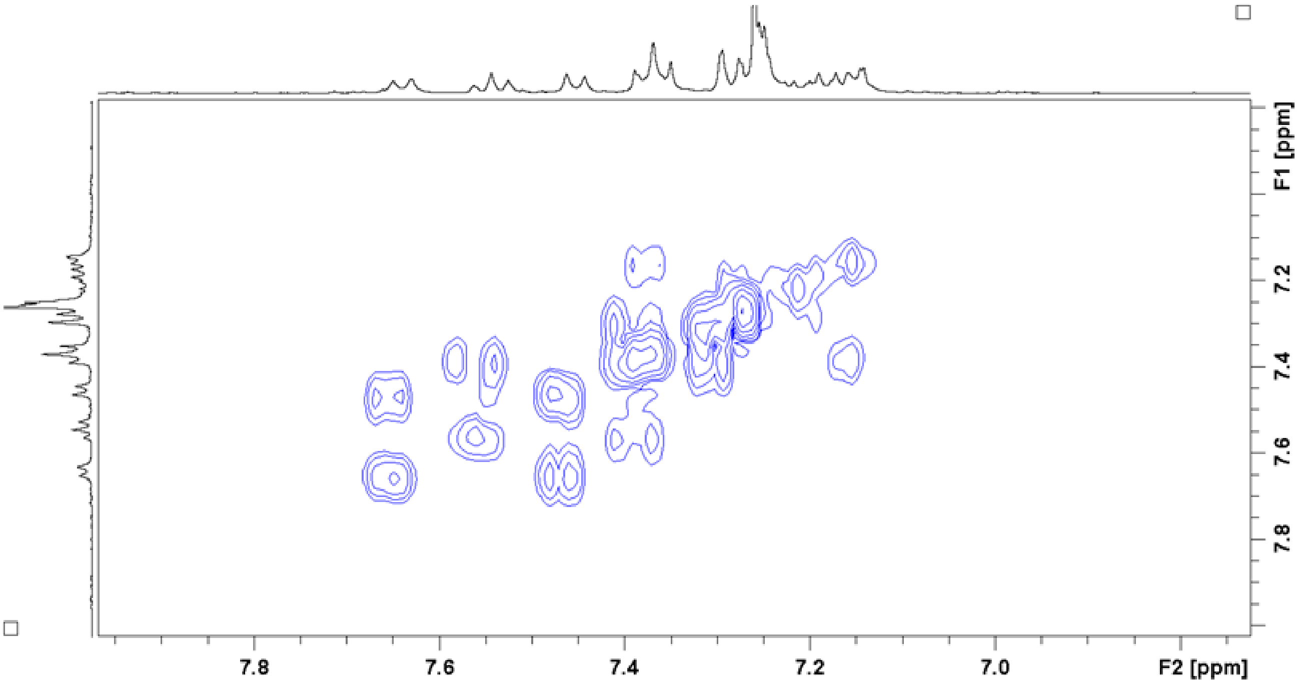

Figure S3. COSY spectrum of **CN-TAI**

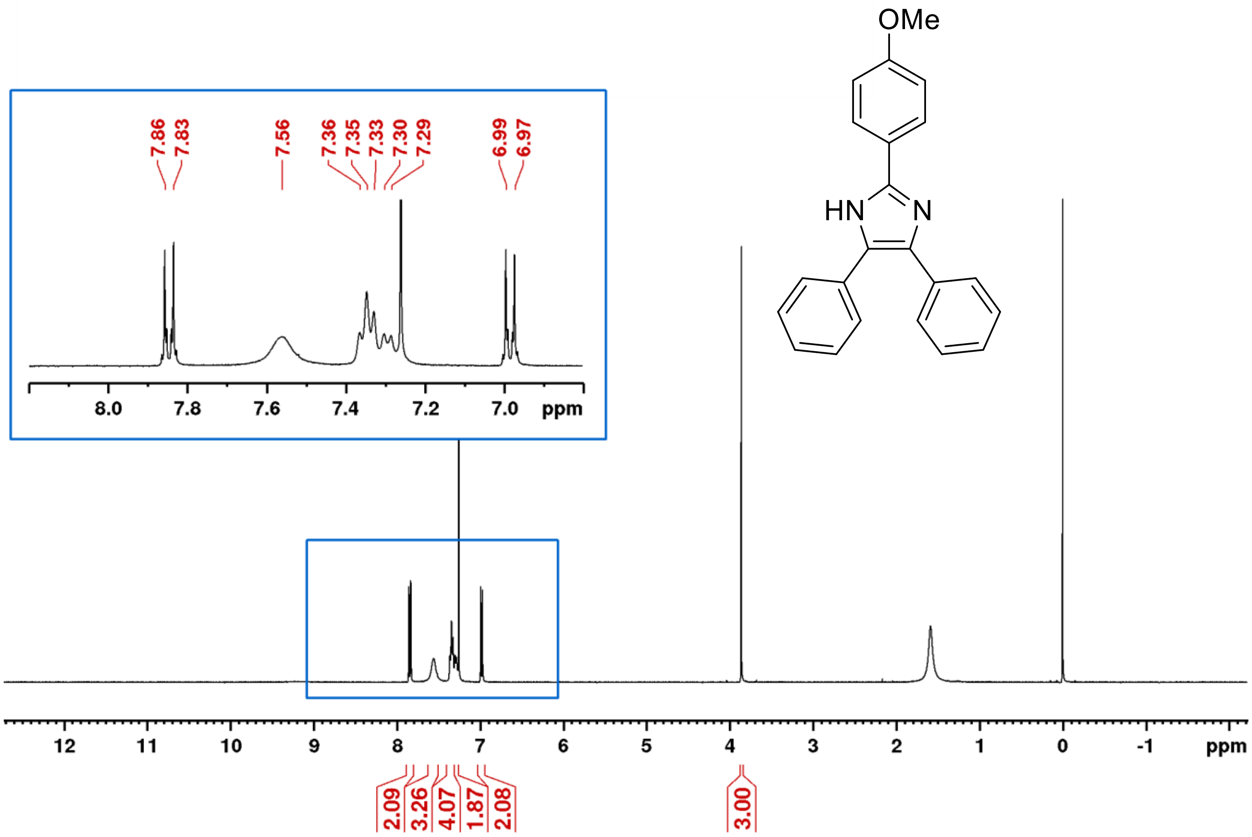

Figure S4. ^{1}H NMR spectrum of **OMe-TAI**

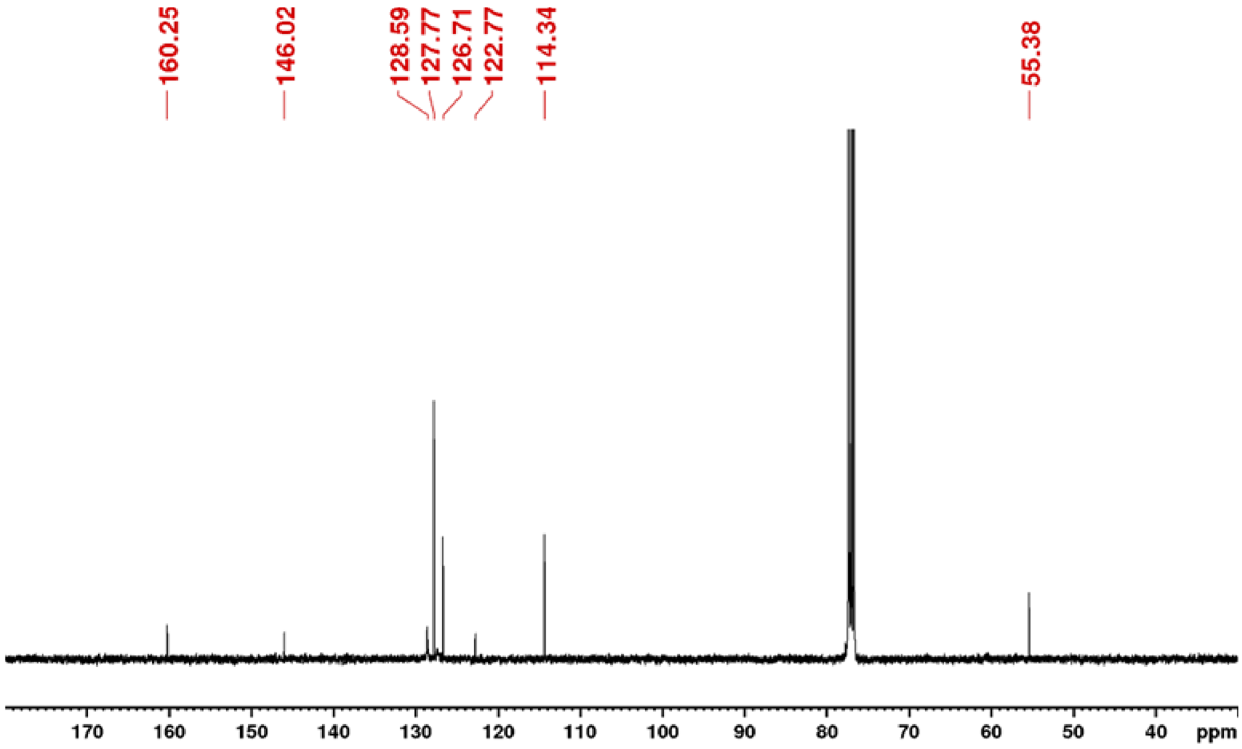

Figure S5. ^{13}C NMR spectrum of **OMe-TAI**

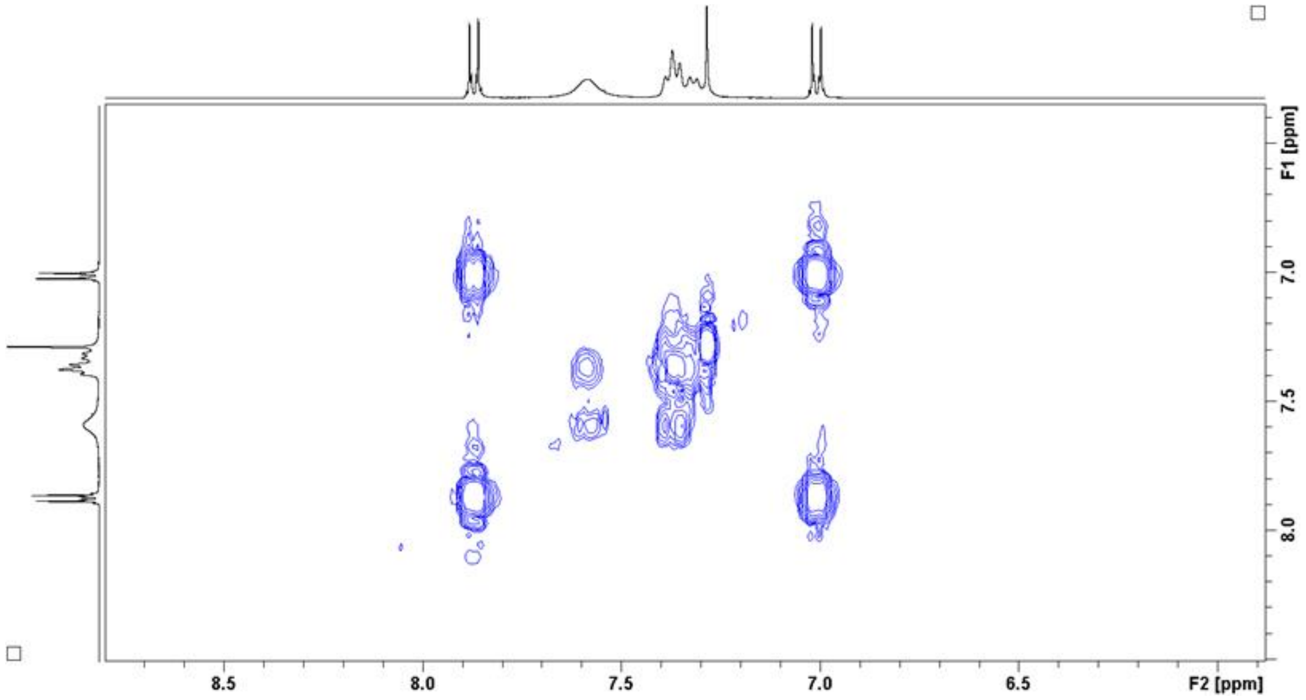

Figure S6. COSY spectrum of **OMe-TA**I

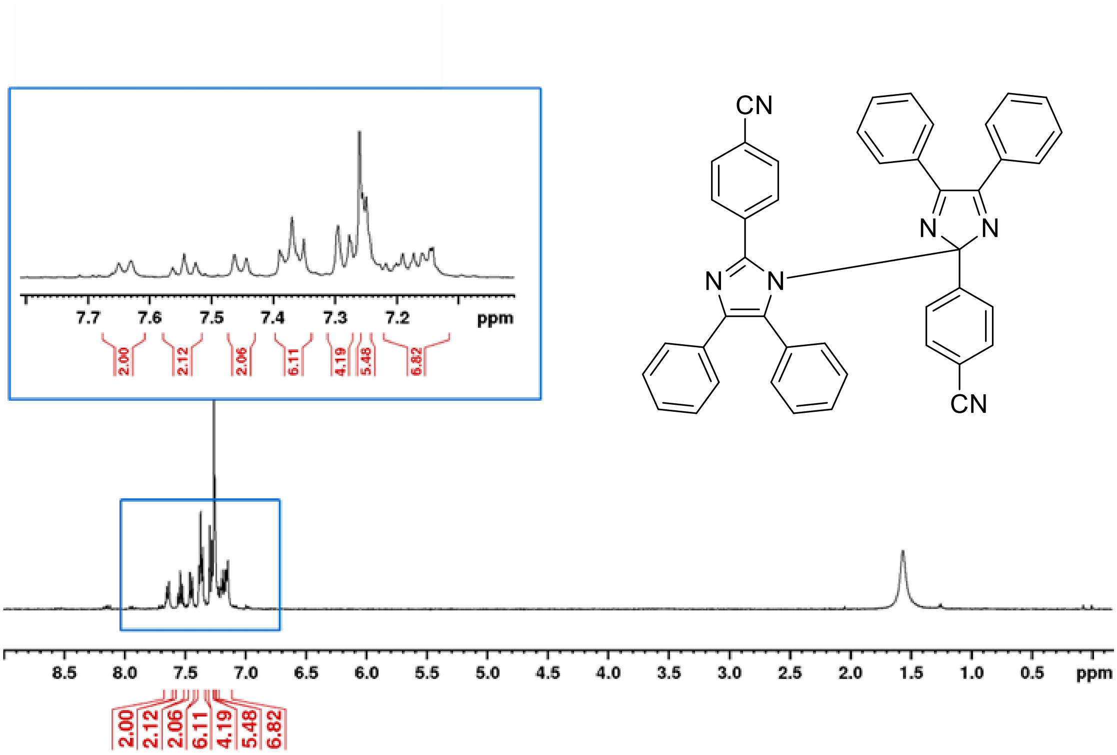

Figure S7. ^{1}H NMR spectrum of **CN-CN**

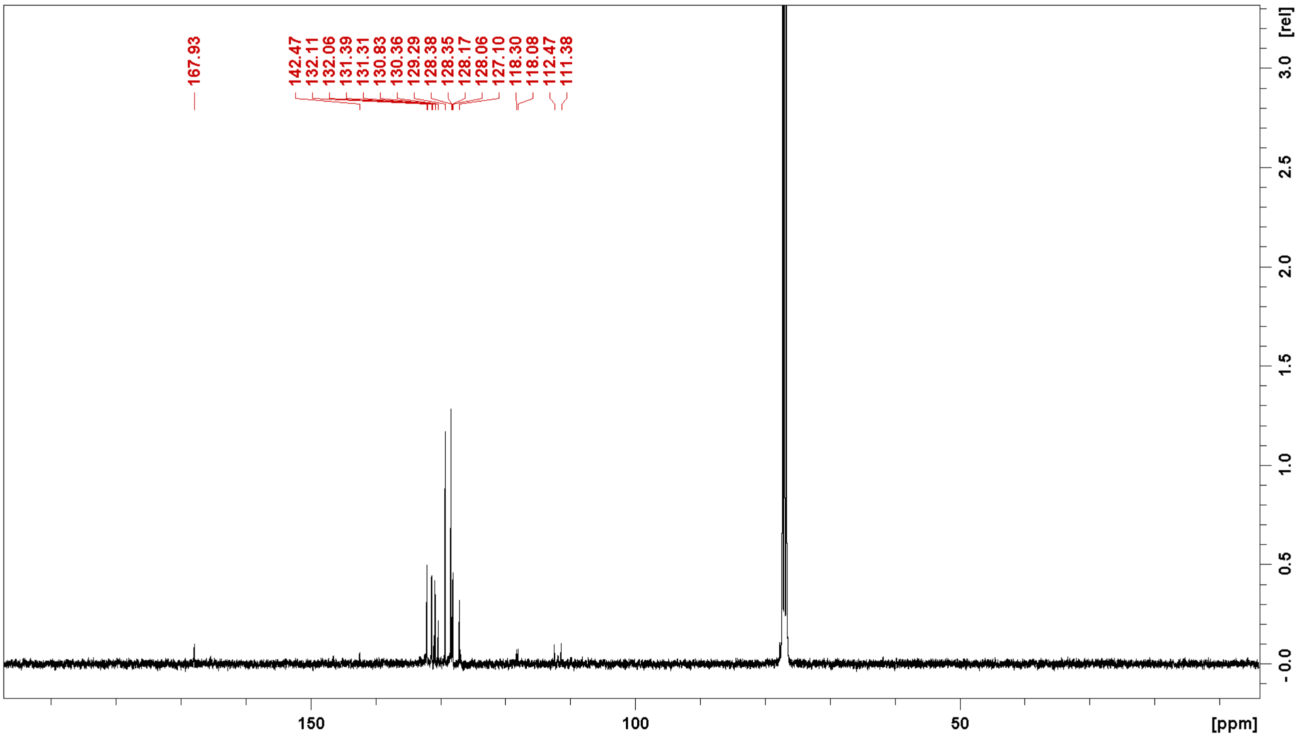

Figure S8. ^{13}C NMR spectrum of **CN-CN**

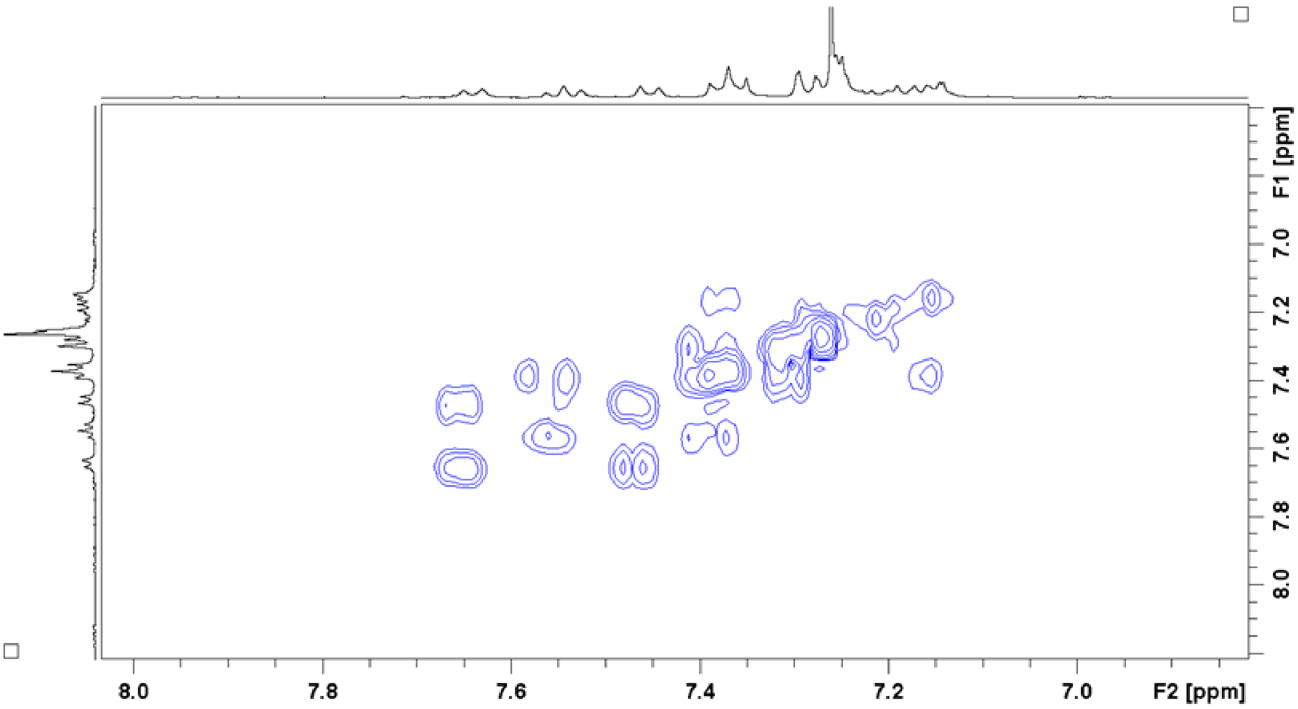

Figure S9. COSY spectrum of **CN-CN**

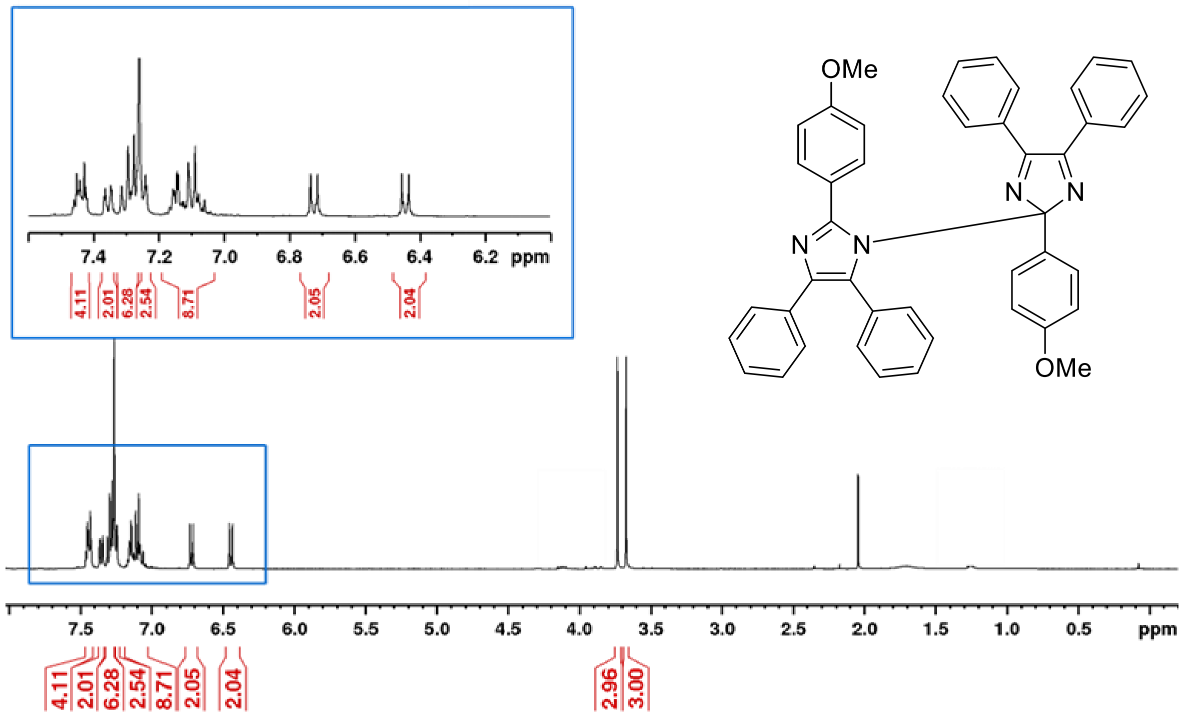

Figure S10. ^{1}H NMR spectrum of **OMe-OMe**

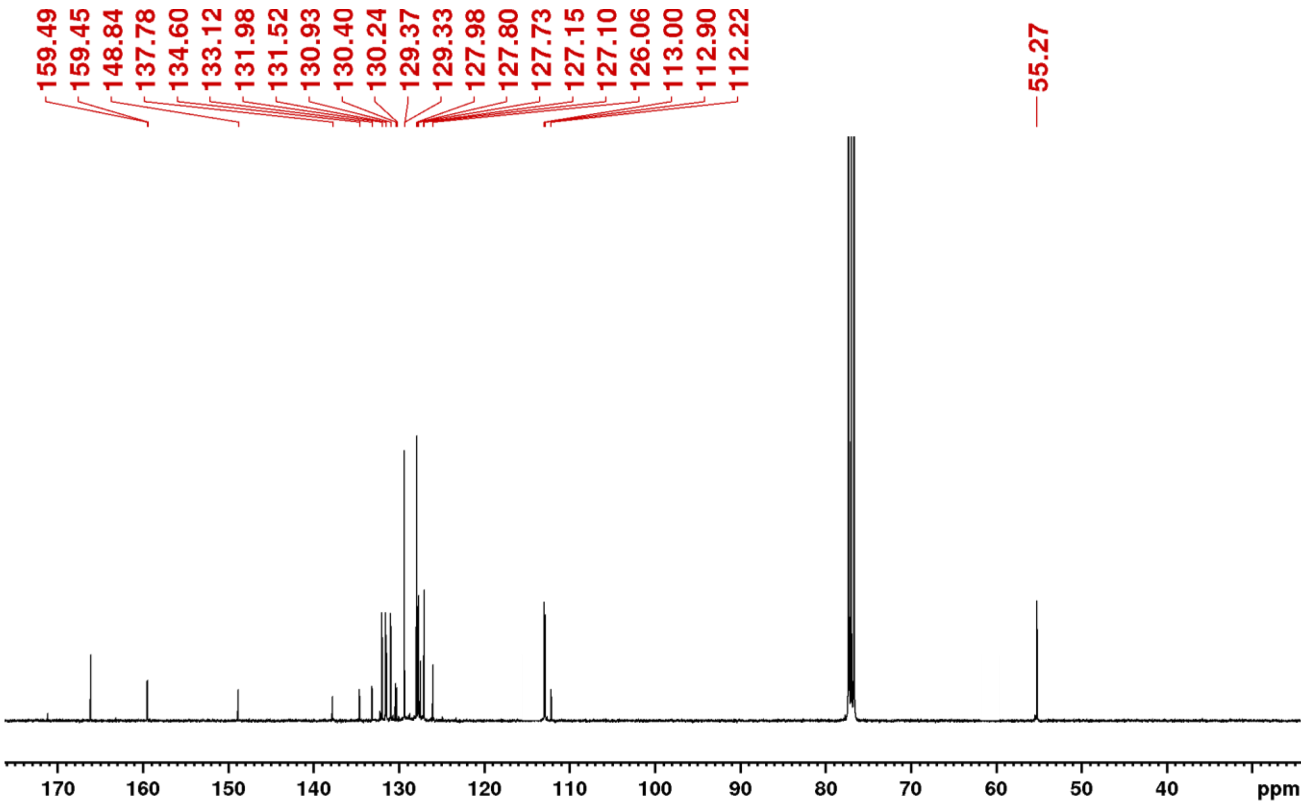

Figure S11. ^{13}C NMR spectrum of **OMe-OMe**

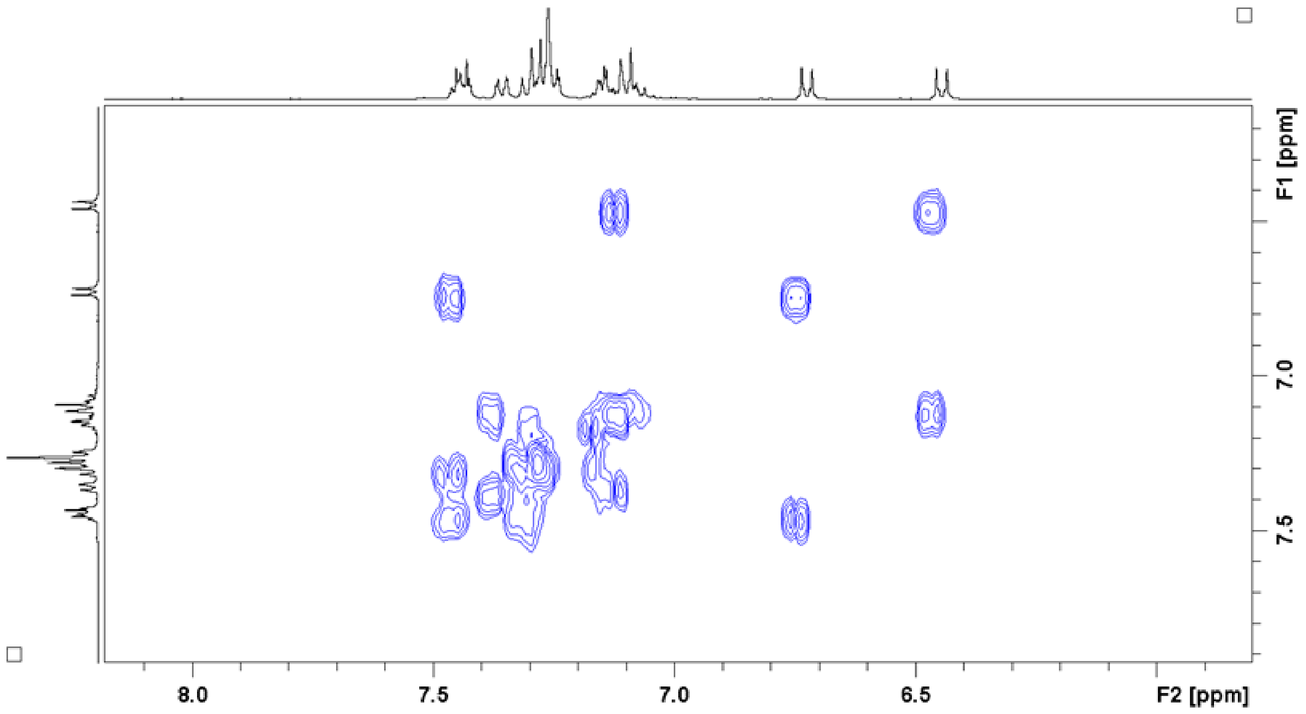

Figure S12. COSY spectrum of **OMe-OMe**

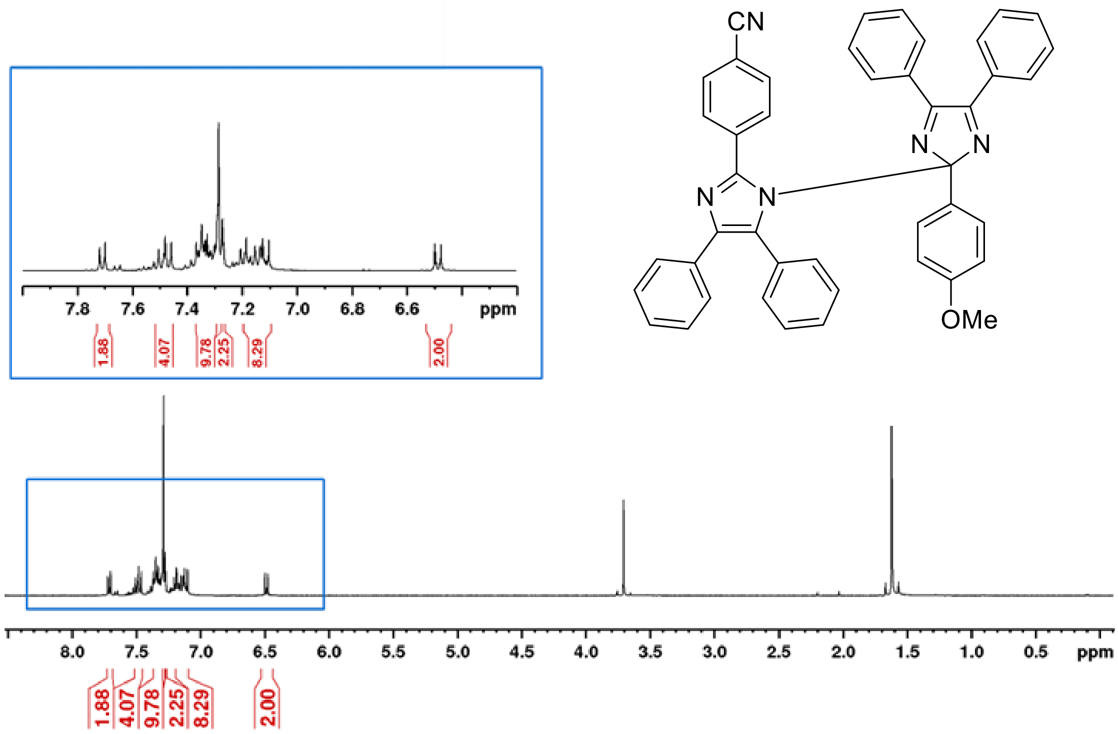

Figure S13. ^{1}H NMR spectrum of **CN-OMe**

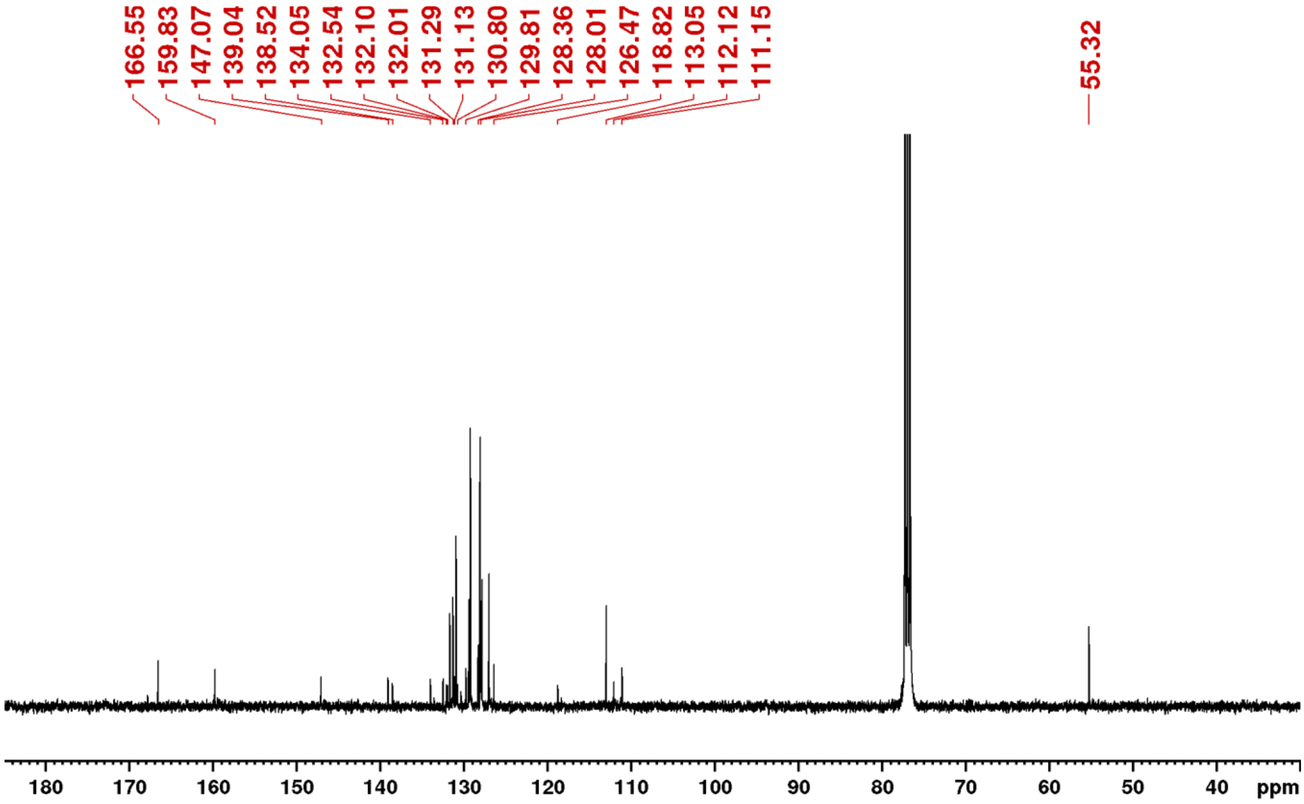

Figure S14. ^{13}C NMR spectrum of **CN-OMe**

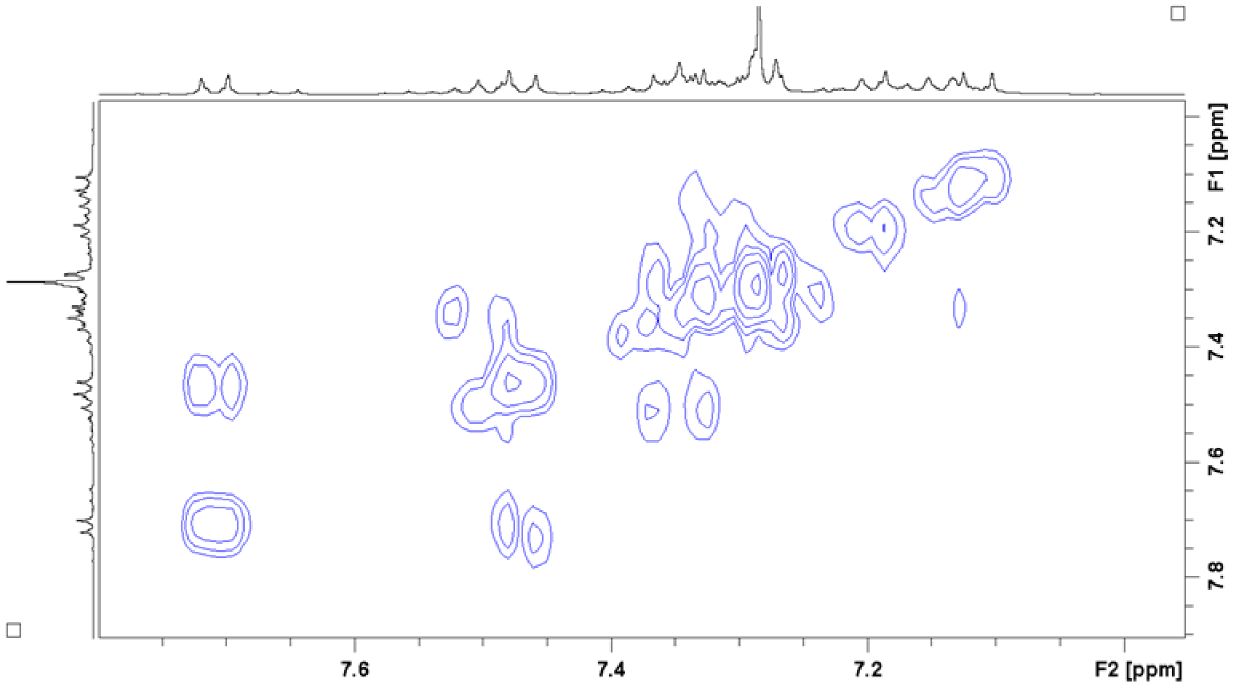

Figure S15. COSY spectrum of **CN-OMe**

Mass Data

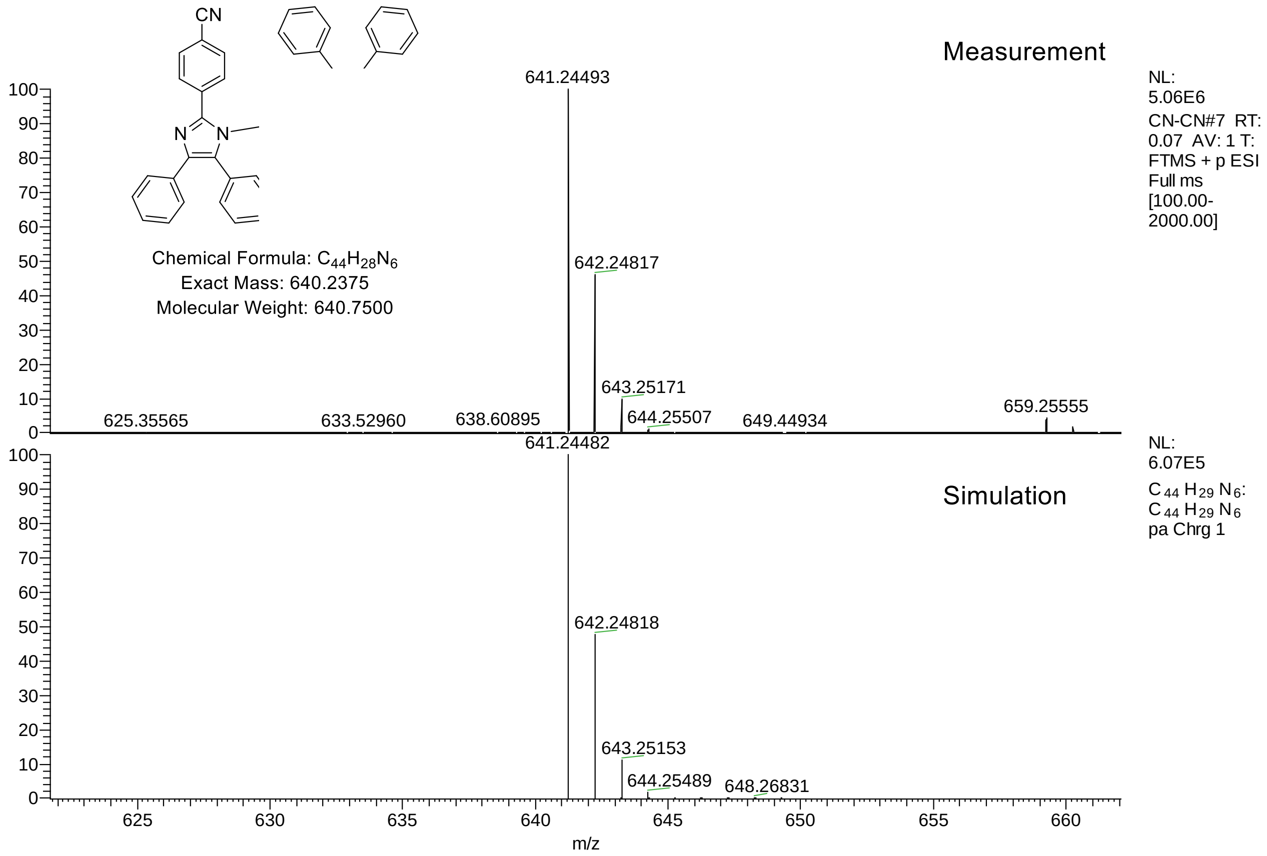

Figure S16. Mass spectrum and simulation of **CN-CN**

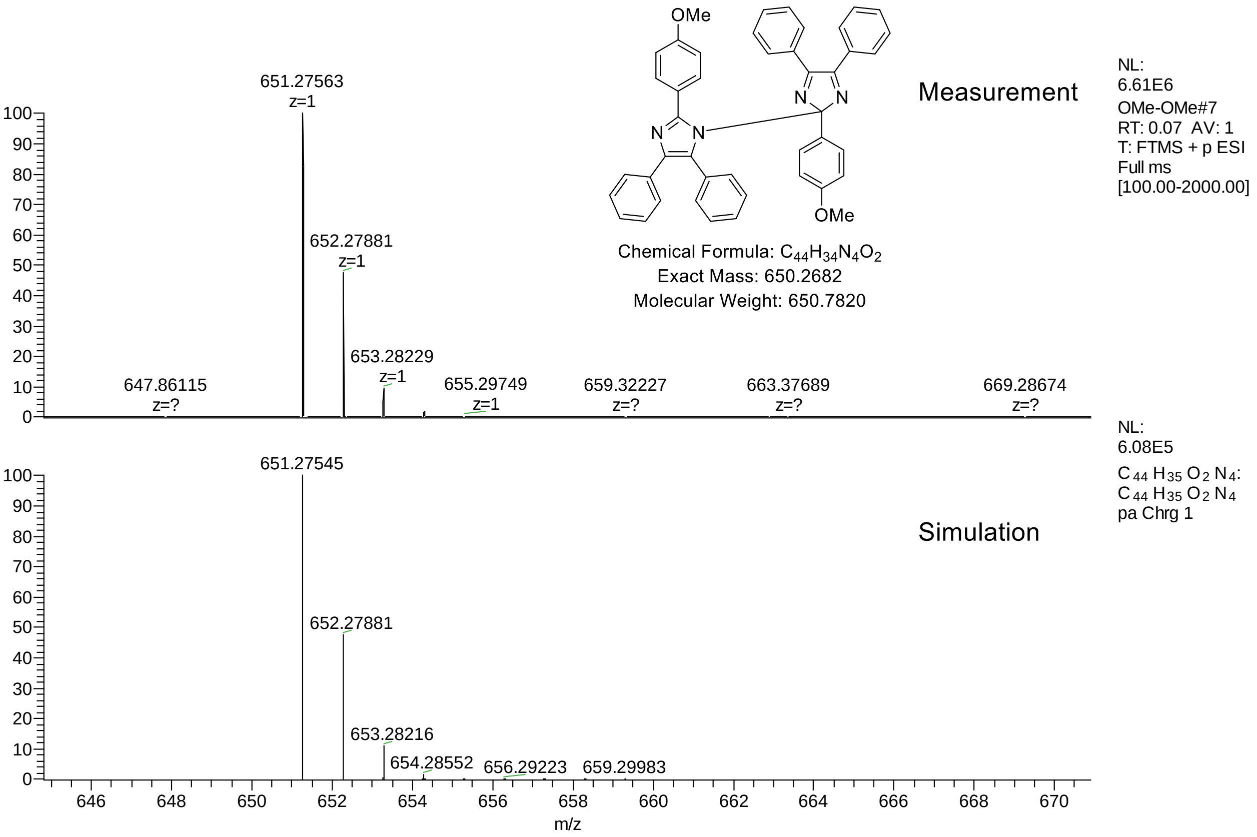

Figure S17. Mass spectrum and simulation of **OMe-OMe**

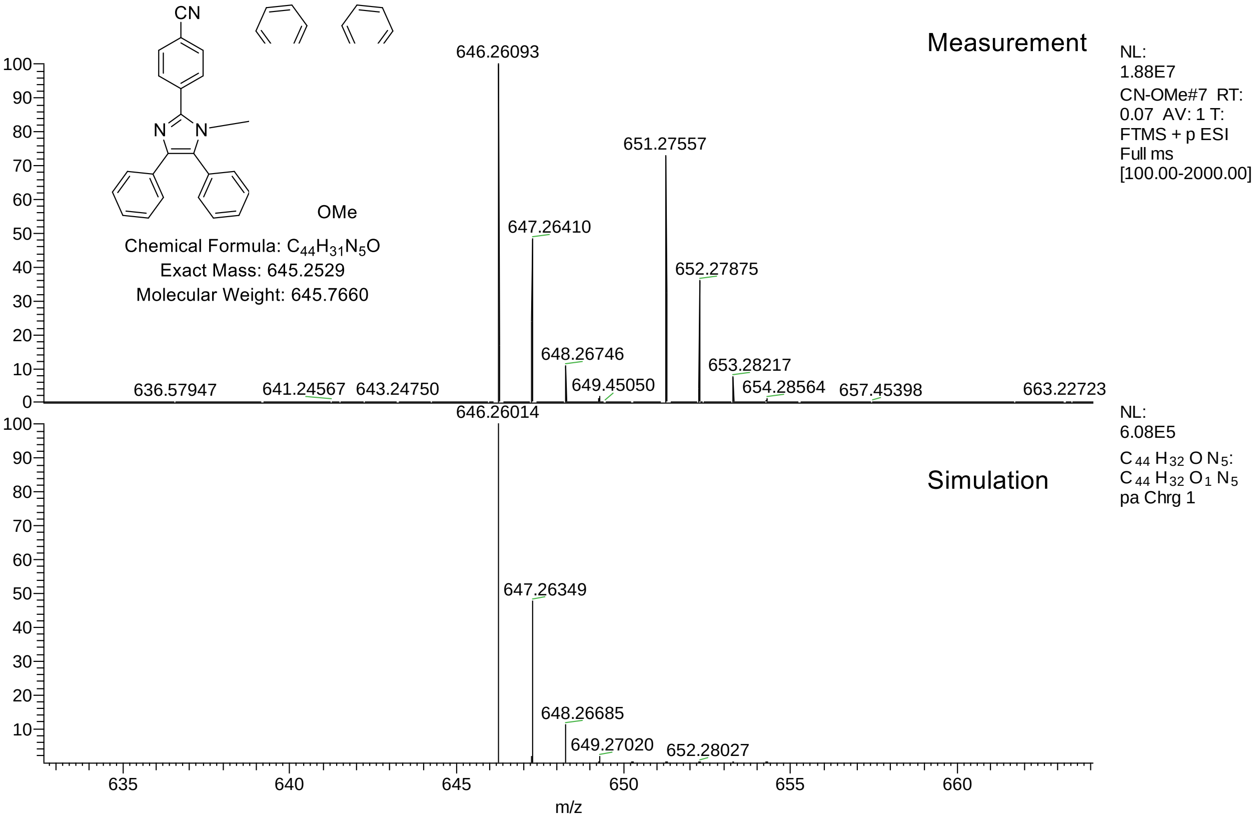

Figure S18. Mass spectrum and simulation of **CN-OMe**

3. UV-vis Absorption Spectra

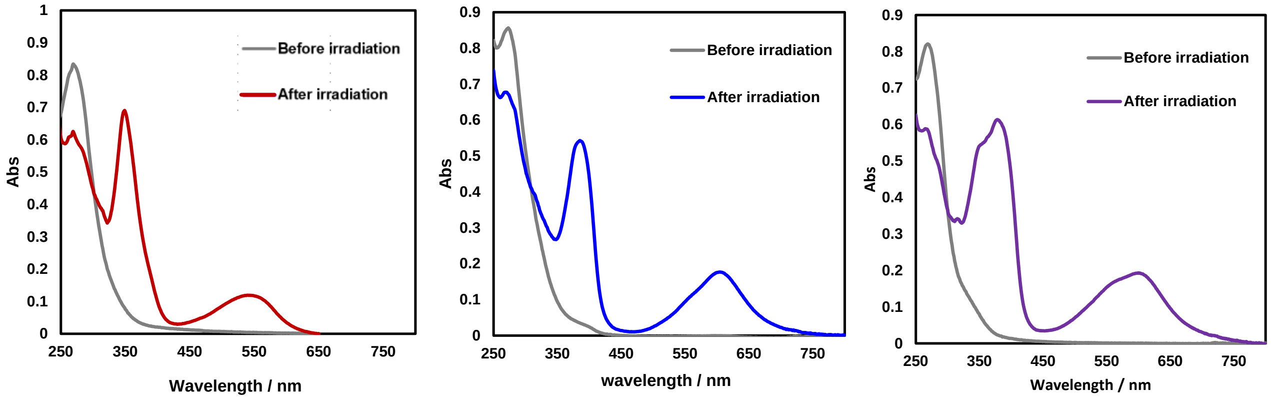

Figure S19. UV-vis absorption spectra of **CN-CN** (1.2×10^{-5} M), **OMe-OMe** (1.2×10^{-5} M) and **CN-OMe** (1.2×10^{-5} M) in CH_2Cl_2 before light (365 nm LED) irradiation and after irradiation.

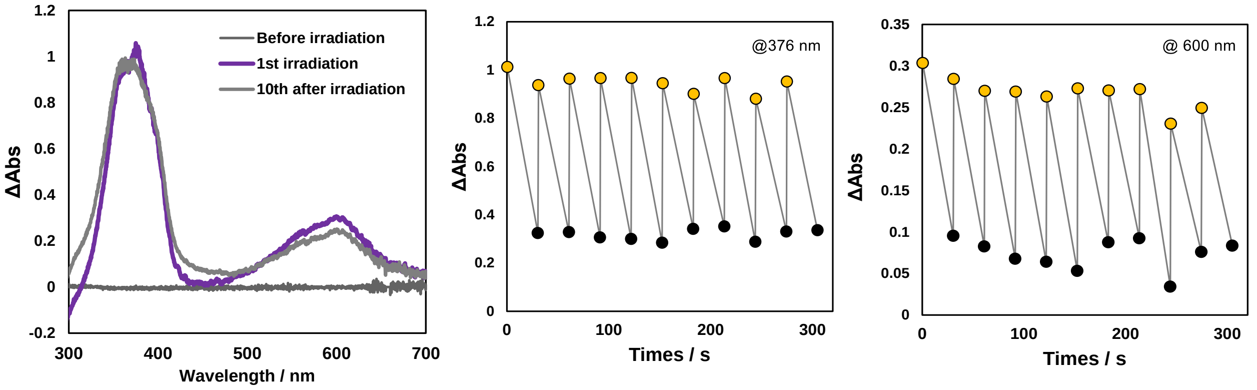

Figure S20. To investigate the photostability of CN-OMe under repeated light irradiation, we conducted a series of ON and OFF cycles over ten repetitions. During the ON state, the sample was irradiated to light (365 nm LED) for 30 seconds in degassed benzene. In the OFF state, light irradiation was stopped, and the sample was kept in the dark for 30 minutes. Changes in absorbance intensity at 376 nm and 603 nm were plotted.

4. Single X-ray Structure Analysis Data

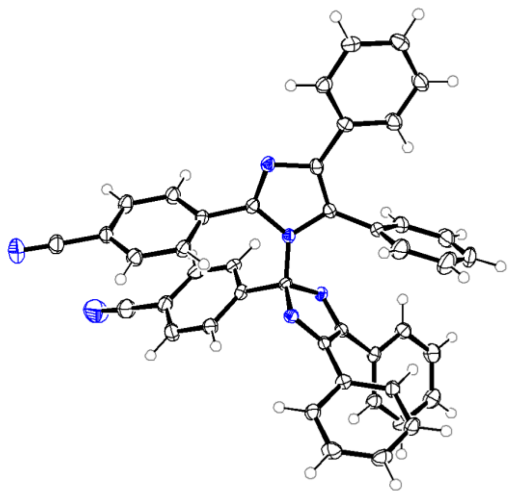

Table S1. X-ray crystal structures of **CN-CN** with thermal ellipsoids (50% probability) and crystal data

Empirical formula	C44 H28 N6	
Formula weight	640.72	
Temperature	100 K	
Radiation type	MoKa	
Wavelength	0.71073 Å	
Crystal system	Monoclinic	
Space group	P 1 21 1	
Unit cell dimensions	a = 9.8629(12) Å	α = 90 °
	b = 11.8736(15) Å	β = 99.790(2) °
	c = 14.2335(18) Å	γ = 90 °
Volume	1642.6(4) $Å^3$	
Z	2	
Goodness-of-fit on F^2	0.809	
Final R indices [I>2sigma(I)]	R1 = 0.0364, wR2 = 0.1036	
R indices (all data)	R1 = 0.0451, wR2 = 0.1128	
Density (calculated)	1.295 Mg/m^3	
Absorption coefficient	0.072 mm^{-1}	
F(000)	372.0	
Crystal size	0.25 × 0.25 × 0.20 mm^3	
Theta range for data collection	2.2473 to 26.8061°	
Index ranges	-10<=h<=12, -15<=k<=11, -18<=l<=16	
Absorption correction	Multi-scan	
Max. and min. transmission	0.98 and 0.87	
Data / restraints / parameters	5920 / 1 / 452	
Detector area resolution	8.3333	
Highest diff. peak and deepest hole	0.213 and -0.178 e.$Å^{-3}$	

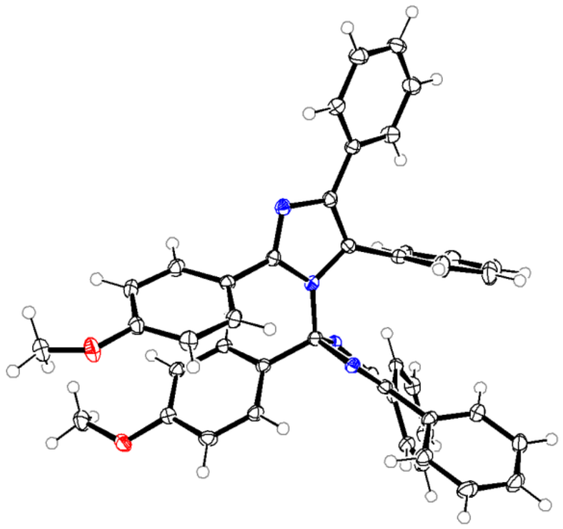

Table S2. X-ray crystal structures of **OMe-OMe** with thermal ellipsoids (50% probability) and crystal data

Empirical formula	C44 H34 N4 O2	
Formula weight	650.75	
Temperature	100 K	
Radiation type	MoKa	
Wavelength	0.71073 Å	
Crystal system	Monoclinic	
Space group	P 1 21 1	
Unit cell dimensions	a = 9.8821(16) Å	α = 90 °
	b = 11.8243(19) Å	β = 101.908(2) °
	c = 14.4510(20) Å	γ = 90 °
Volume	1652.1(5) $Å^3$	
Z	2	
Goodness-of-fit on F^2	0.784	
Final R indices [I>2sigma(I)]	R1 = 0.0355, wR2 = 0.0997	
R indices (all data)	R1 = 0.0438, wR2 = 0.1083	
Density (calculated)	1.308 Mg/m^3	
Absorption coefficient	0.081 mm^{-1}	
F(000)	684.0	
Crystal size	0.25 × 0.25 × 0.20 mm^3	
Theta range for data collection	2.11 to 29.21°	
Index ranges	-13<=h<=12, -11<=k<=16, -17<=l<=19	
Absorption correction	Multi-scan	
Max. and min. transmission	0.99 and 0.85	
Data / restraints / parameters	5954 / 1 / 454	
Detector area resolution	8.3333	
Highest diff. peak and deepest hole	0.240 and -0.194 e.$Å^{-3}$	

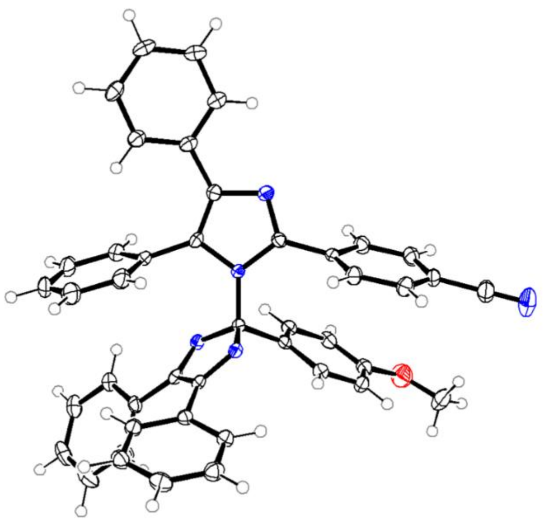

Table S3. X-ray crystal structures of **CN-CN** with thermal ellipsoids (50% probability) and crystal data

Empirical formula	C44 H31 N5 O1	
Formula weight	643.82	
Temperature	100 K	
Radiation type	MoKa	
Wavelength	0.71073 Å	
Crystal system	Monoclinic	
Space group	P 1 21/c 1	
Unit cell dimensions	a = 14.1977(6) Å	α = 90 °
	b = 11.8122(5) Å	β = 98.796(4) °
	c = 20.1325 (9) Å	γ = 90 °
Volume	3336.6(3) $Å^3$	
Z	4	
Goodness-of-fit on F^2	1.027	
Final R indices [I>2sigma(I)]	R1 = 0.0497, wR2 = 0.0980	
R indices (all data)	R1 = 0.0799, wR2 = 0.1071	
Density (calculated)	1.282 Mg/m^3	
Absorption coefficient	0.0717 mm^{-1}	
F(000)	1348.0	
Crystal size	0.05 × 0.09 × 0.16 mm^3	
Theta range for data collection	2.02 to 29.95°	
Index ranges	-17<=h<=17, -13<=k<=14, -24<=l<=124	
Absorption correction	Multi-scan	
Data / restraints / parameters	6100 / 0 / 453	
Detector area resolution	10.0000	
Highest diff. peak and deepest hole	0.560 and -0.420 $e.Å^{-3}$	

5. Rate constant for Radical-Radical Coupling

Calibration Curve 1

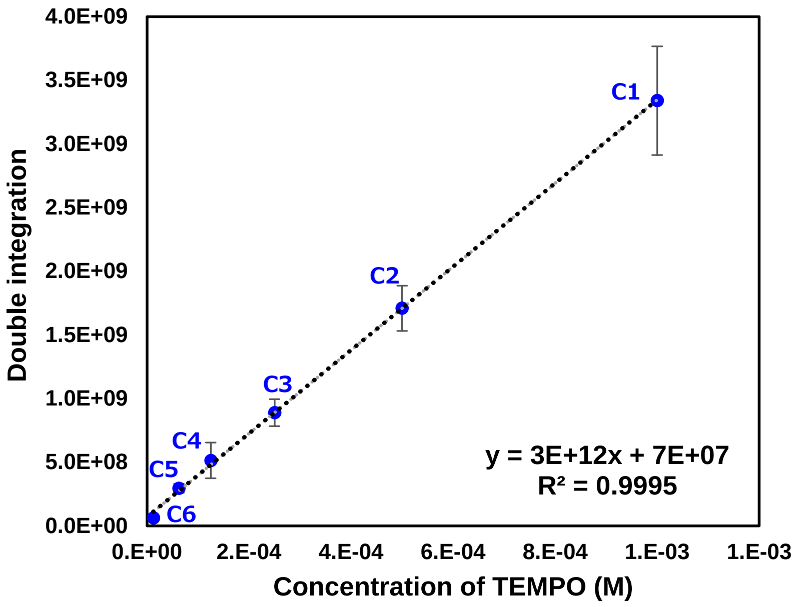

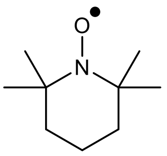

TEMPO

C1 = 1.0×10^{-3} M
C2 = 5.0×10^{-4} M
C3 = 2.5×10^{-4} M
C4 = 1.3×10^{-4} M
C5 = 6.3×10^{-5} M
C6 = 1.3×10^{-5} M

Figure S21. The calibration curve for spin concentration of TEMPO in degassed benzene solution at 294 K was established. Double integration values were obtained for each concentration sample (C1 to C6), and the concentration of TAIR was determined from the slope of the calibration curve.

Calibration Curve 2

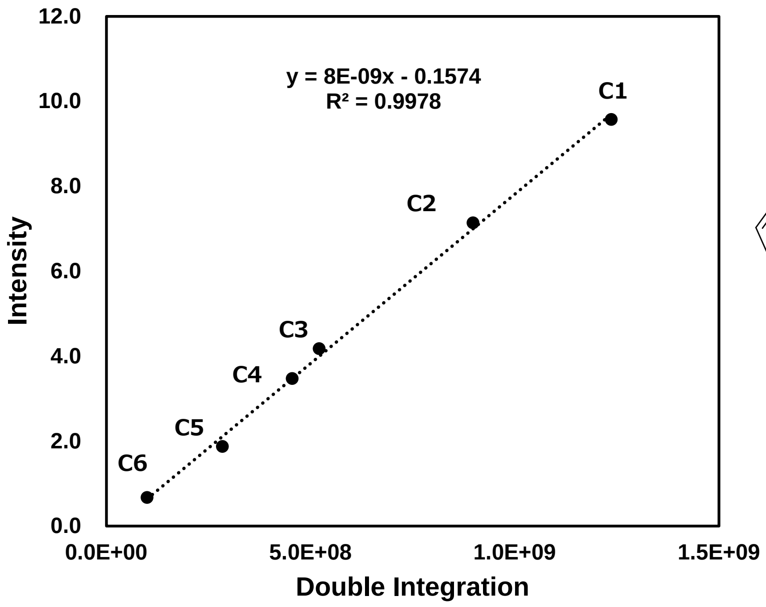

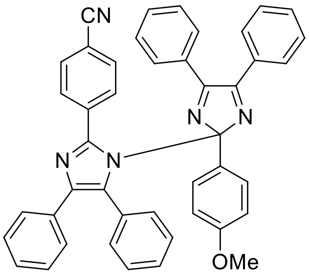

C1 = 9.9×10^{-4} M
C2 = 4.9×10^{-4} M
C3 = 2.5×10^{-4} M
C4 = 1.5×10^{-4} M
C5 = 6.3×10^{-5} M
C6 = 1.5×10^{-5} M

Figure S22. The calibration curve for converting the double integration of HABI to intensity on the decay curve of radical-radical coupling.

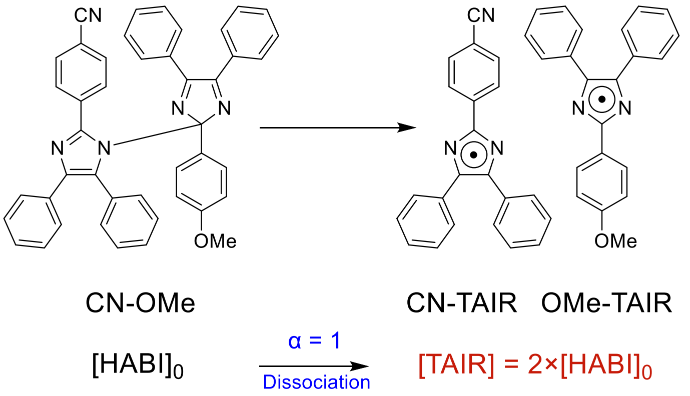

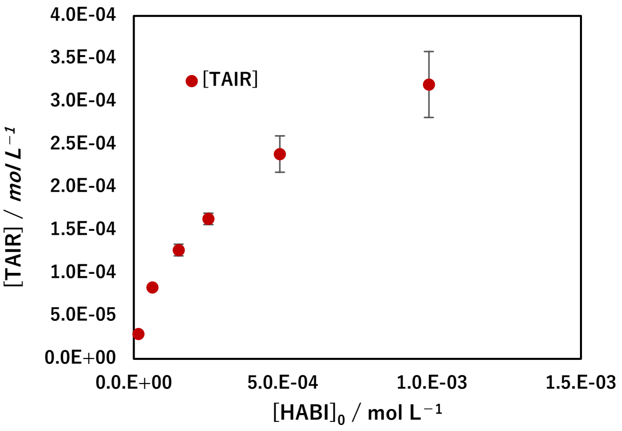

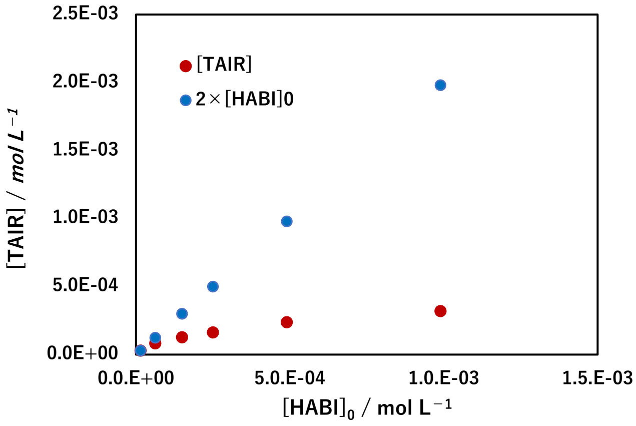

Entry	**[HABI]$_0$**	**1**	**2**	**3**	**Average**	**Error**	**[TAIR]** (*mol L^{-1}*)	**2 × [HABI]$_0$** (*mol L^{-1}*)	**Dissociation α** ([TAIR] / 2 × [HABI]$_0$)
1	9.9×10^{-4}	3.7×10^{-4}	2.7×10^{-4}	3.2×10^{-4}	3.2×10^{-4}	3.8×10^{-5}	3.2×10^{-4}	2.0×10^{-3}	0.16
2	4.9×10^{-4}	2.7×10^{-4}	2.3×10^{-4}	2.2×10^{-4}	2.4×10^{-4}	2.1×10^{-5}	2.4×10^{-4}	9.8×10^{-4}	0.24
3	2.5×10^{-4}	1.5×10^{-4}	1.6×10^{-4}	1.7×10^{-4}	1.6×10^{-4}	6.8×10^{-5}	1.6×10^{-4}	5.0×10^{-4}	0.33
4	1.5×10^{-4}	1.4×10^{-4}	1.2×10^{-4}	1.3×10^{-4}	1.3×10^{-4}	6.8×10^{-6}	1.3×10^{-4}	3.0×10^{-4}	0.42
5	6.3×10^{-5}	8.4×10^{-5}	8.6×10^{-5}	8.0×10^{-5}	8.4×10^{-5}	2.6×10^{-6}	8.4×10^{-5}	1.3×10^{-4}	0.67
6	1.5×10^{-5}	3.0×10^{-5}	2.9×10^{-5}	3.0×10^{-5}	2.9×10^{-5}	4.2×10^{-7}	2.9×10^{-5}	3.0×10^{-5}	0.98

Figure S23. The yield of TAIR and the dissociation (α) in each concentration. Since TAIR is theoretically generated in two equivalents from the initial concentration of HABI, the α was defined as [TAIR] / 2 × [HABI]$_0$. The α in this experiment was measured when the sample was irradiated with a 365 nm LED for 30 s in benzene solution at 294 K. Under high concentration conditions, the α value significantly decreased due to the lower light transmittance.

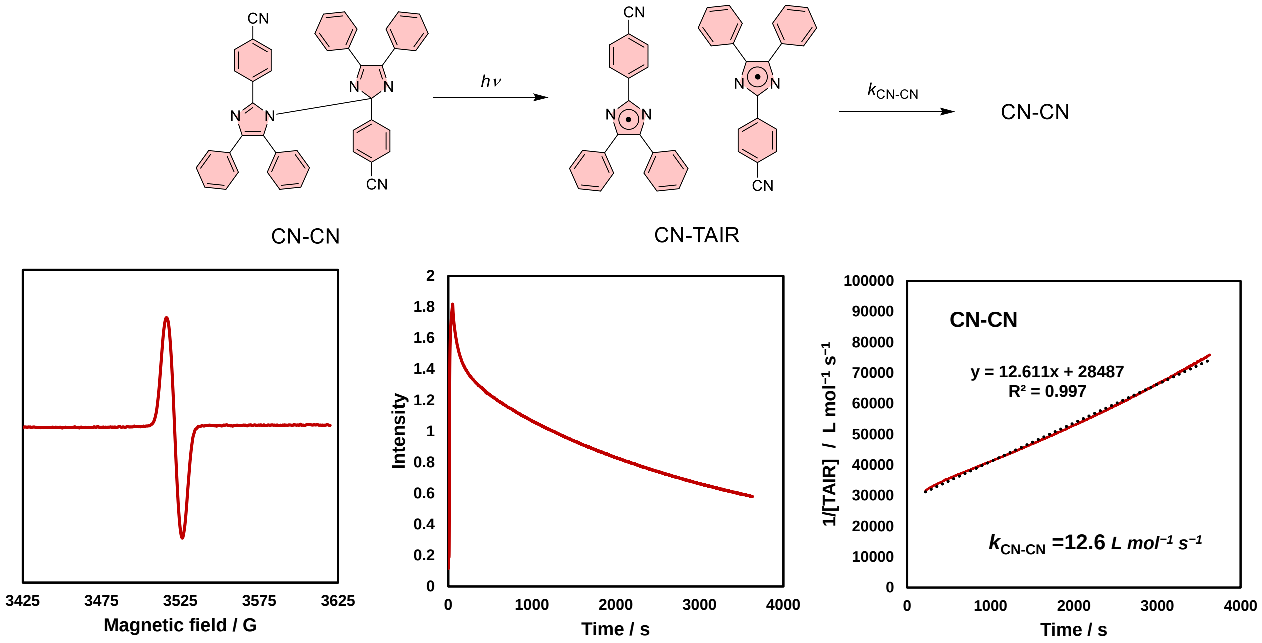

Figure S24. The EPR signal and decay profile of CN-CN. The concentration of TAIR was determined using calibration curves 1 and 2 (Figure S25 and S26). The rate constant for radical coupling was obtained from the slope of the second-order reaction plot (Eq.1).

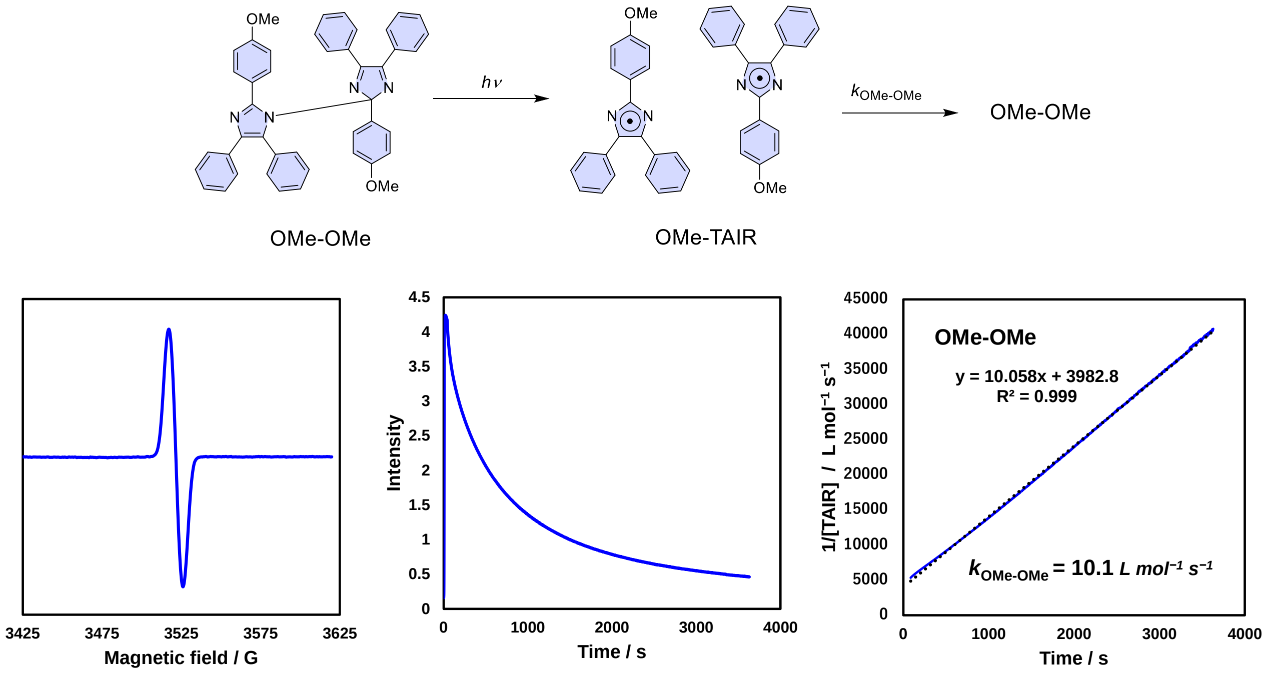

Figure S25. The EPR signal and decay profile of OMe-OMe. The concentration of TAIR was determined using calibration curves 1 and 2 (Figure S25 and S26). The rate constant for radical coupling was obtained from the slope of the second-order reaction plot (Eq.1).

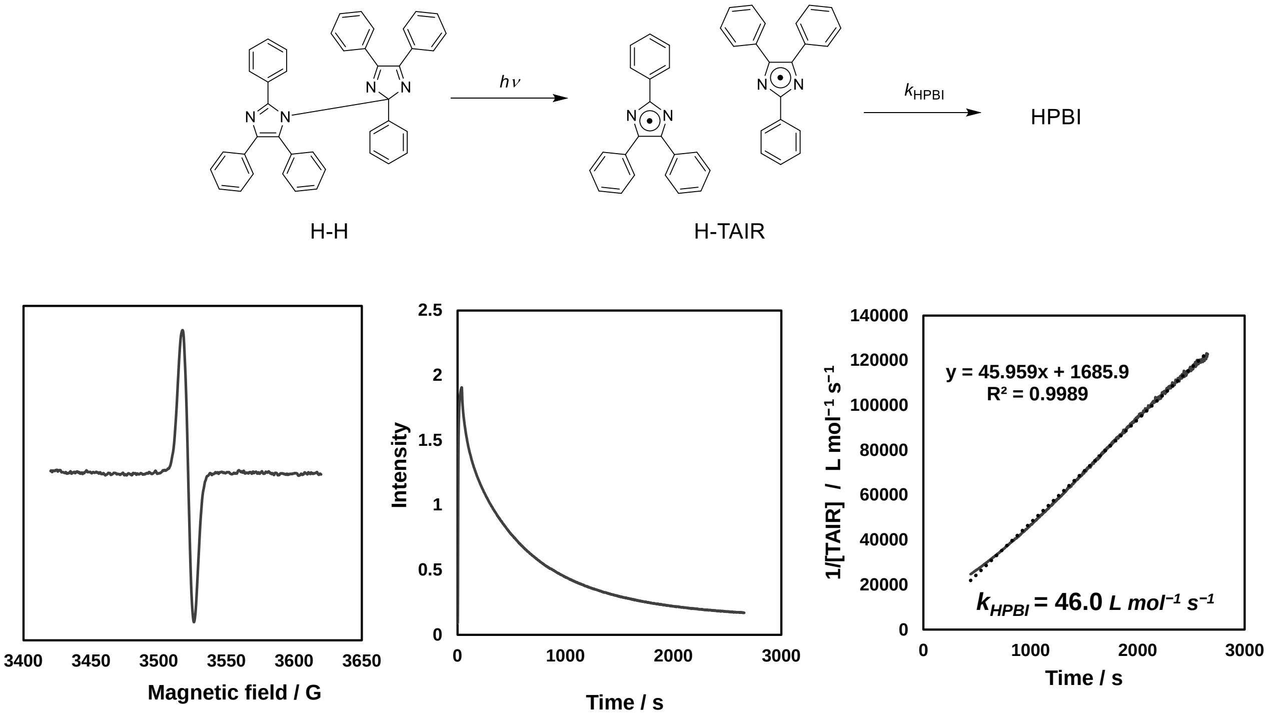

Figure S26. The EPR signal and decay profile of HPBI. The concentration of TAIR was determined using calibration curves 1 and 2 (Figure S25 and S26). The rate constant for radical coupling was obtained from the slope of the second-order reaction plot (Eq.1).

Reaction rate equation for radical-radical cross coupling

$$2[R] \xrightarrow{k} [P]$$

$$\frac{d[R]}{dt} = -k[R]^2$$

$$\int_{[R]_0}^{[R]} \frac{1}{[R]^2} d[R] = -\int_{t_0}^{t} k\, dt$$

$$\frac{1}{[R]_0} - \frac{1}{[R]} = -kt \qquad \text{(Eq.1)}$$

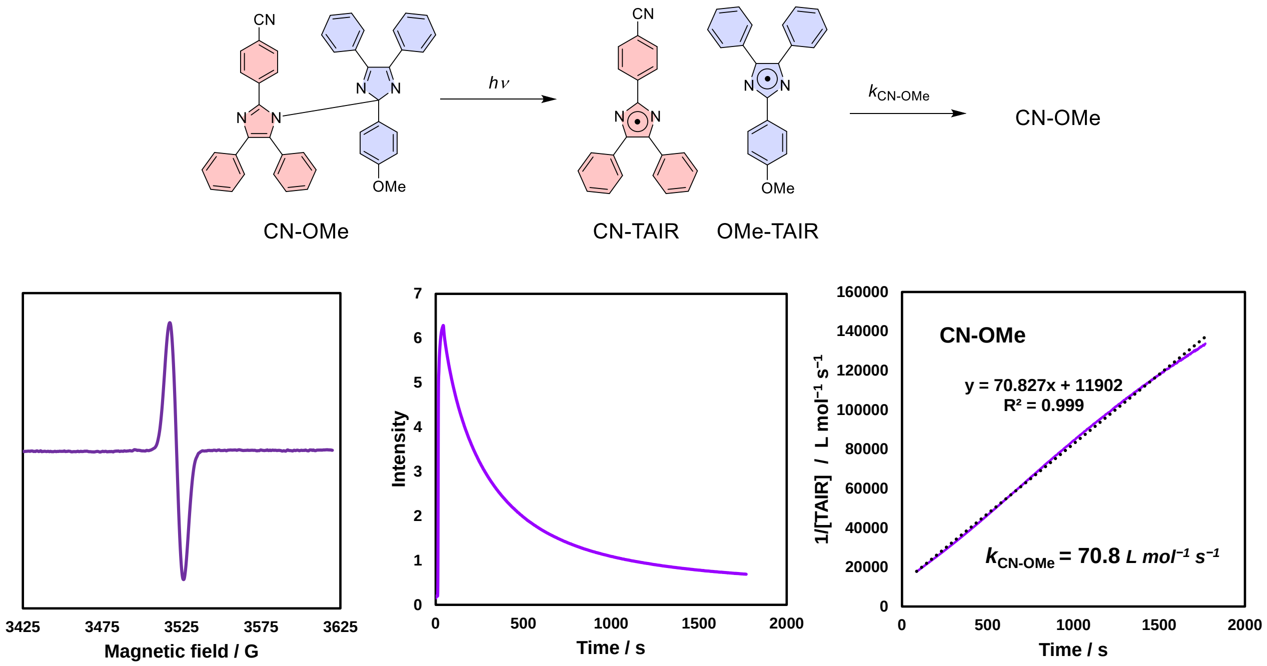

Figure S27. The EPR signal and decay profile of CN-OMe. The concentration of TAIR was determined using calibration curves 1 and 2 (Figure S25 and S26). The rate constant for radical coupling was obtained from the slope of the second-order reaction plot. In the case of CN-OMe, based on the UV-vis absorption spectrum results, we assumed that the presence of two radicals in equal amounts (R1 = R2), and the second-order rate constant was determined(Eq.2).

Reaction rate equation for radical-radical cross coupling

$$[R_1] + [R_2] \xrightarrow{k} [P]$$

$$\frac{d[R_1]}{dt} = -k[R_1][R_2]$$

where the concentrations of R_1 and R_2 are equal

$$[R_1] \approx [R_2]$$

$$\frac{d[R_1]}{dt} = -k[R_1]^2$$

$$\int_{[R_1]_0}^{[R_1]} \frac{1}{[R_1]^2} d[R_1] = -\int_{t_0}^{t} k\, dt$$

$$\frac{1}{[R_1]_0} - \frac{1}{[R_1]} = -kt \qquad \text{(Eq.2)}$$

6. Photoreaction for Each HABI Derivatives

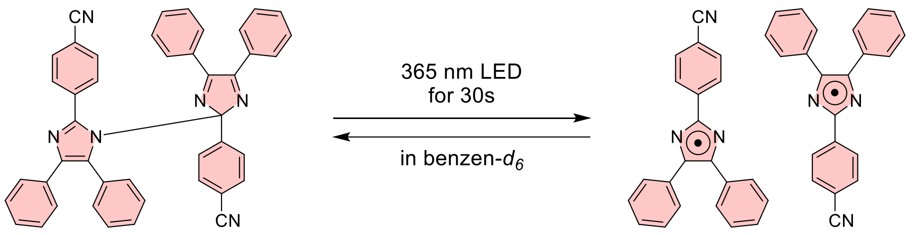

CN-CN CN-TAIR

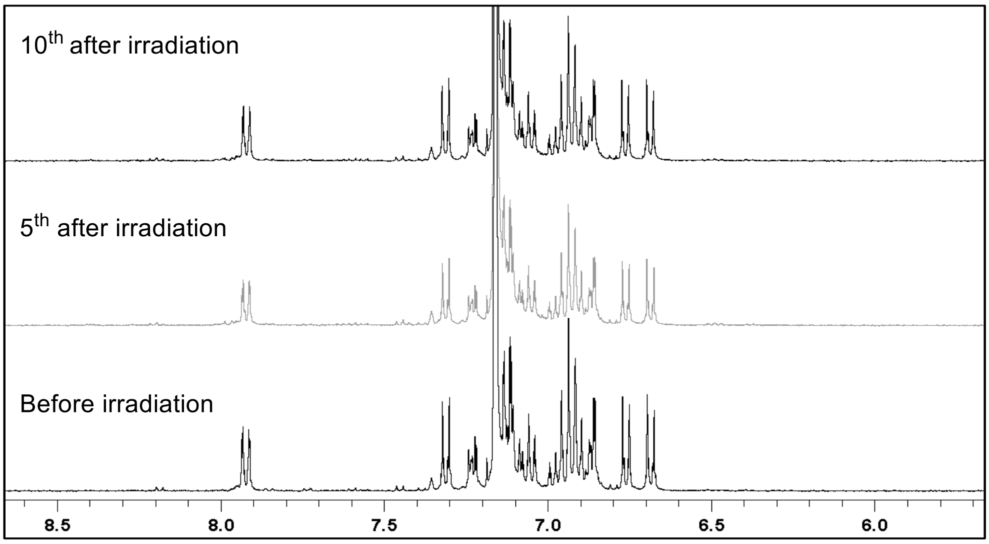

Figure S28. ^{1}H NMR spectra of CN-CN in degassed benzene-d_8 (5.6×10^{-3}) before and several after light irradiation (30 s with a 365 nm LED).

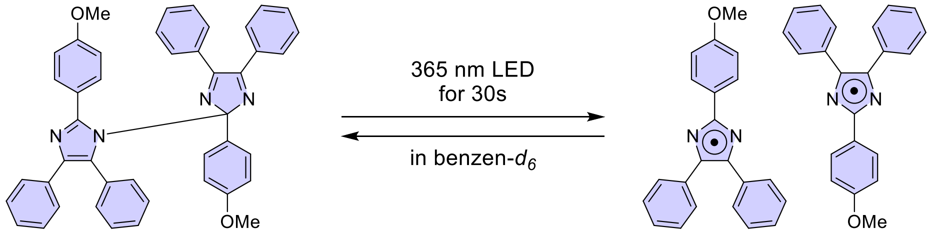

OMe-OMe OMe-TAIR

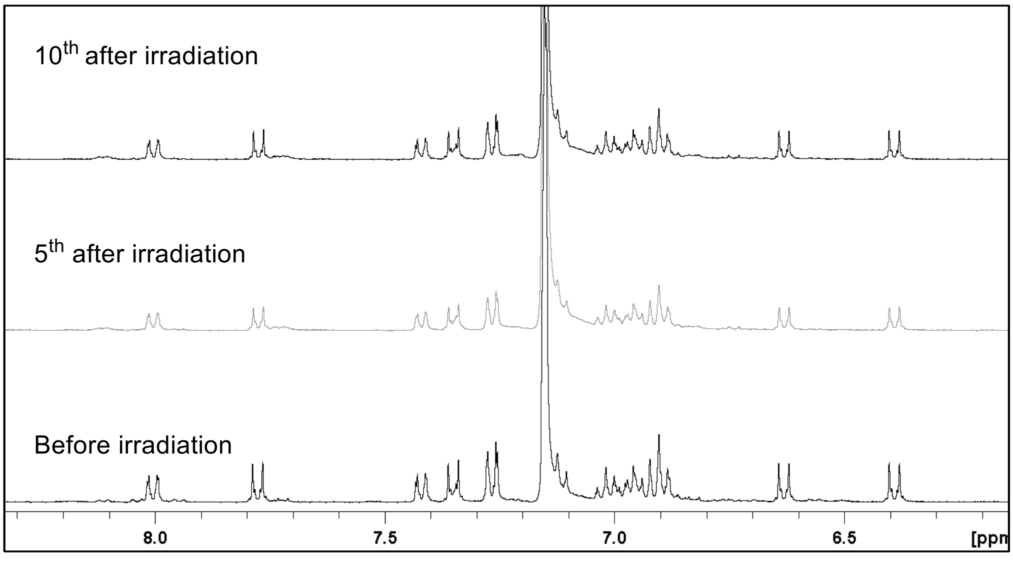

Figure S29. ^{1}H NMR spectra of OMe-OMe in degassed benzene-d_8 (5.6×10^{-3}) before and several after light irradiation (30 s with a 365 nm LED).

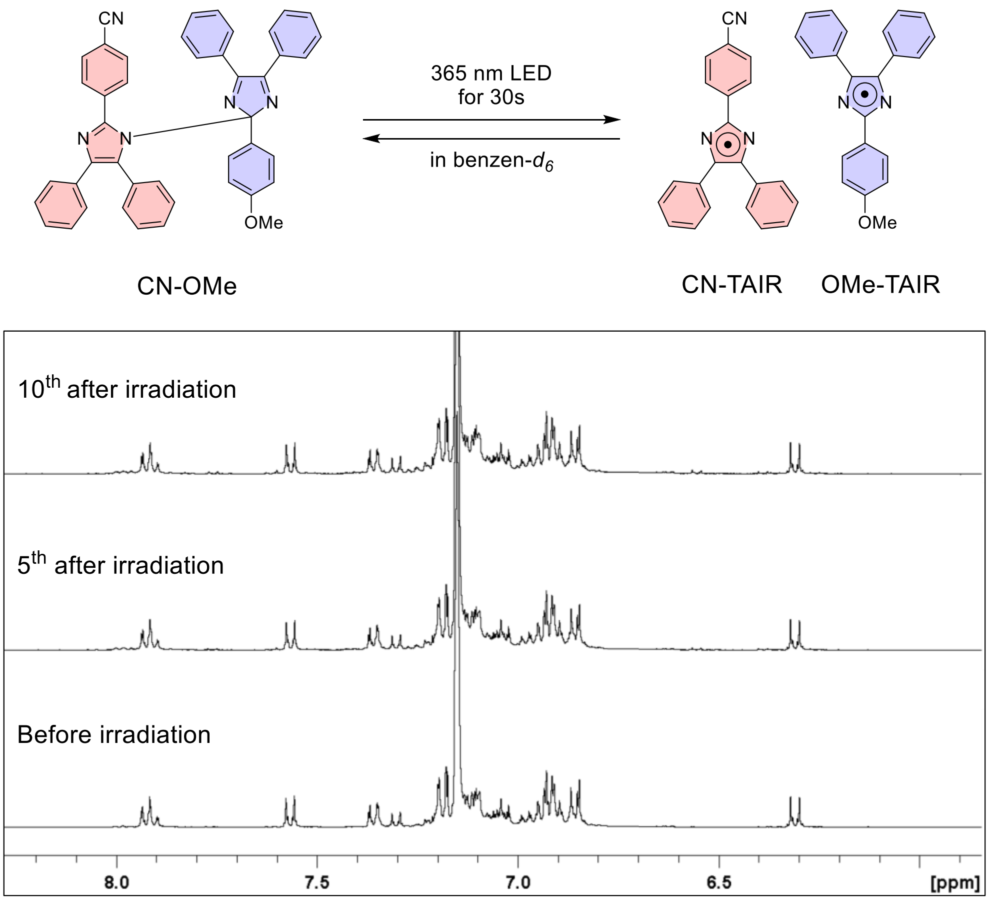

Figure S30. ^{1}H NMR spectra of CN-OMe in degassed benzene-d_8 (6.6$\times$10^{-3}) before and several after light irradiation (30 s with a 365 nm LED).

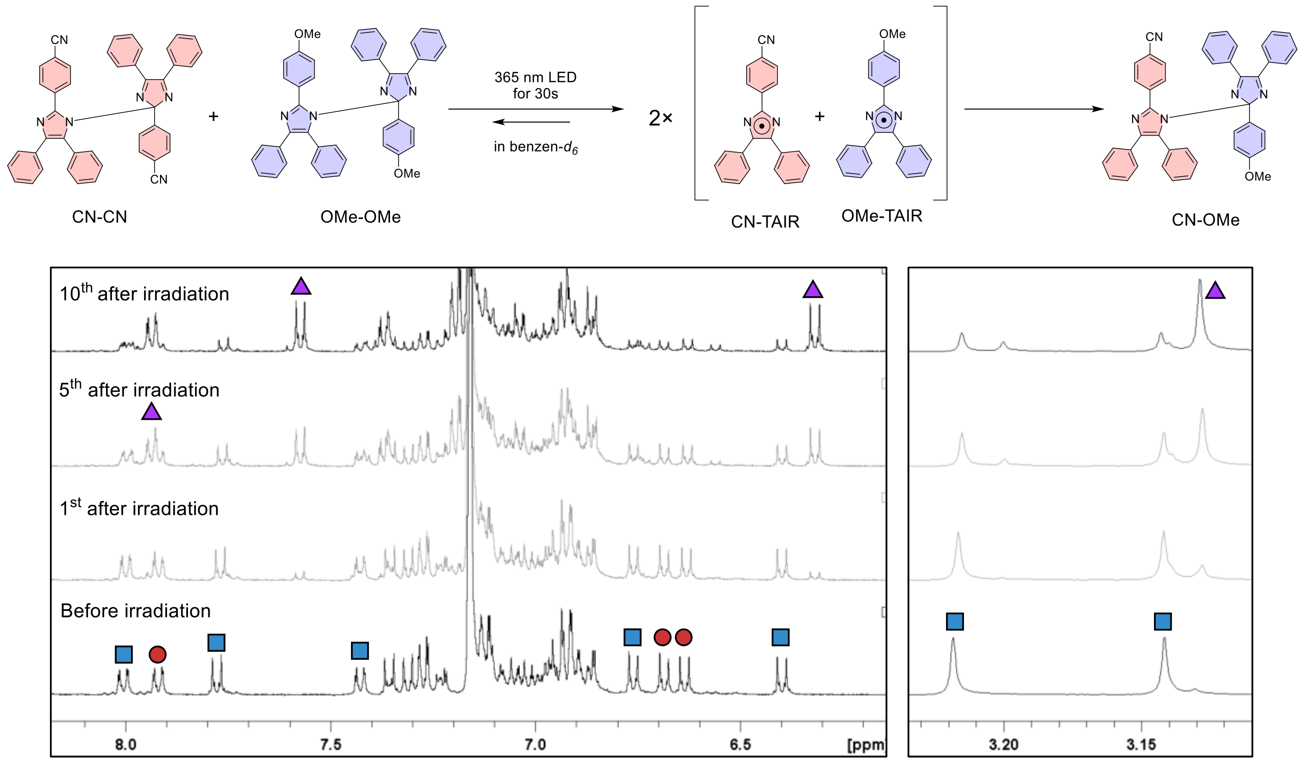

Figure S31. ^{1}H NMR spectra of the mixture sample for CN-CN and OMe-OMe (CN-CN; 4.9$\times$10$^{-3,}$ OMe-OMe; 5.2 $\times$10^{-3}). in degassed benzene-d_8 before and several after light irradiation (30 s with a 365 nm LED).

6. Photoreaction at Low Temperature

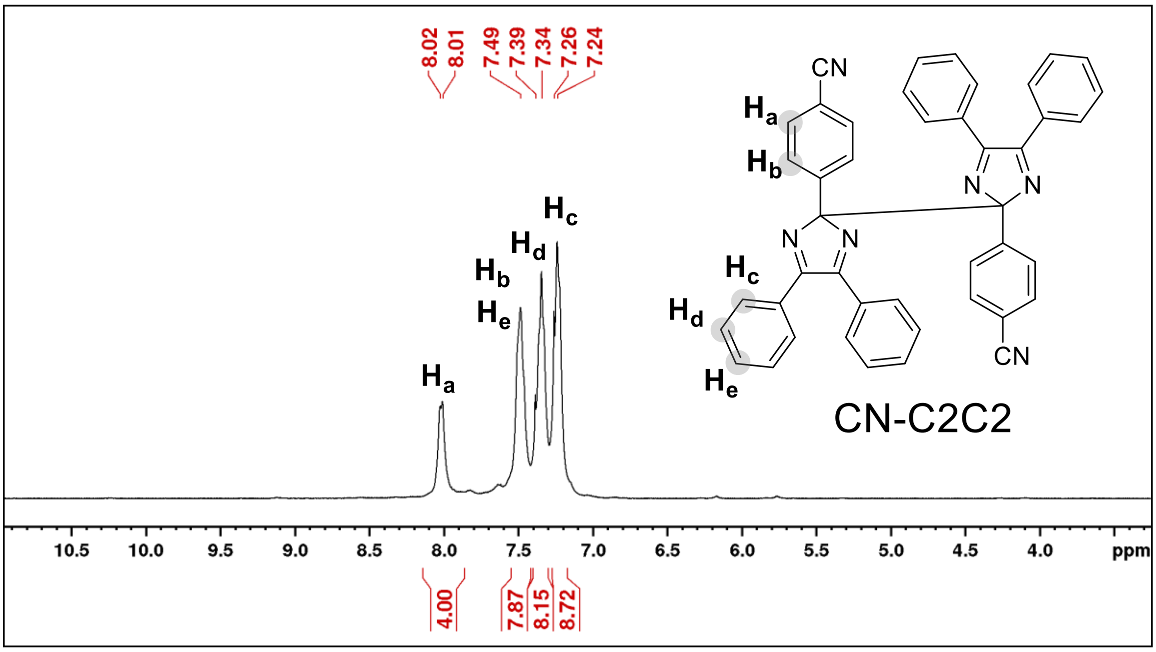

Figure S32. ^{1}H NMR spectrum of **CN-C2C2** at 220 K

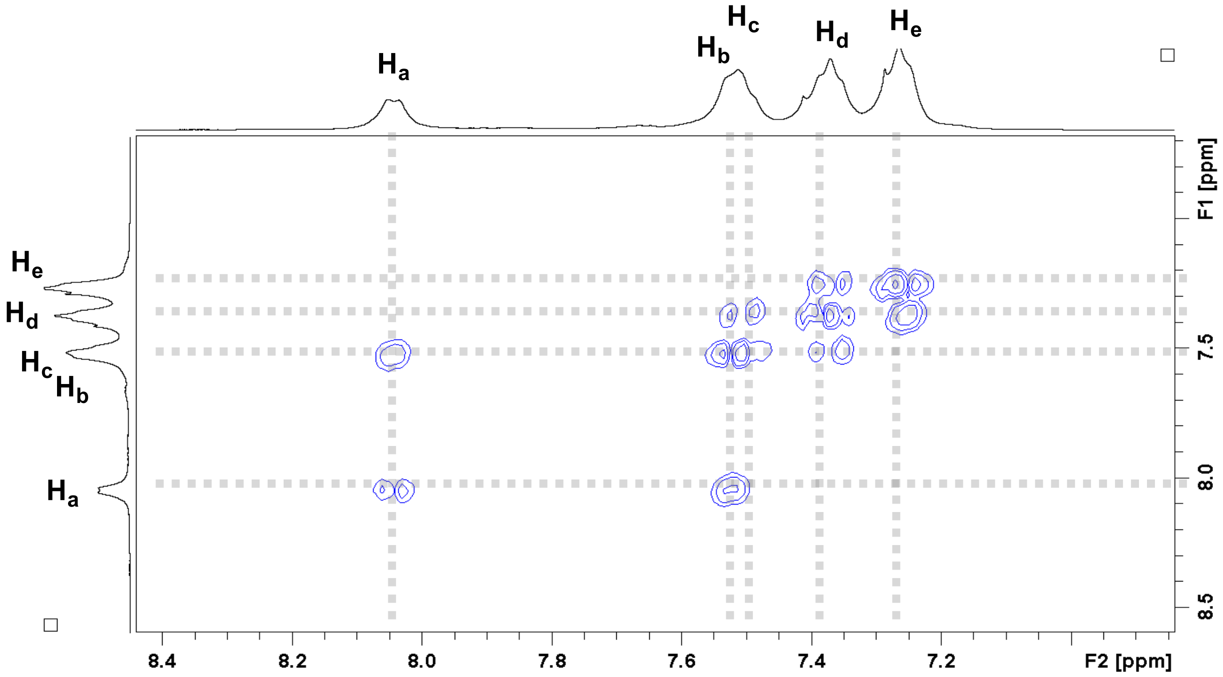

Figure S33. COSY spectrum of **CN-C2C2** at 220 K

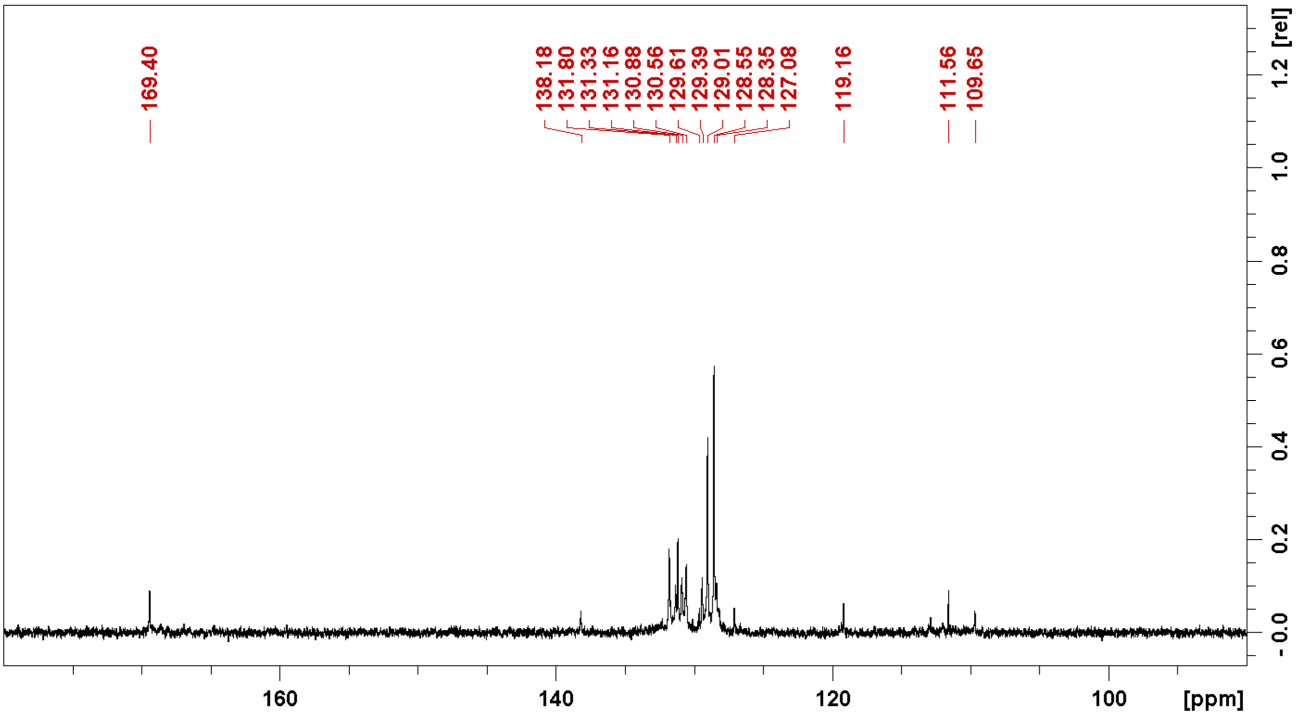

Figure S34. ^{13}C NMR spectrum of **CN-C2C2** at 220 K

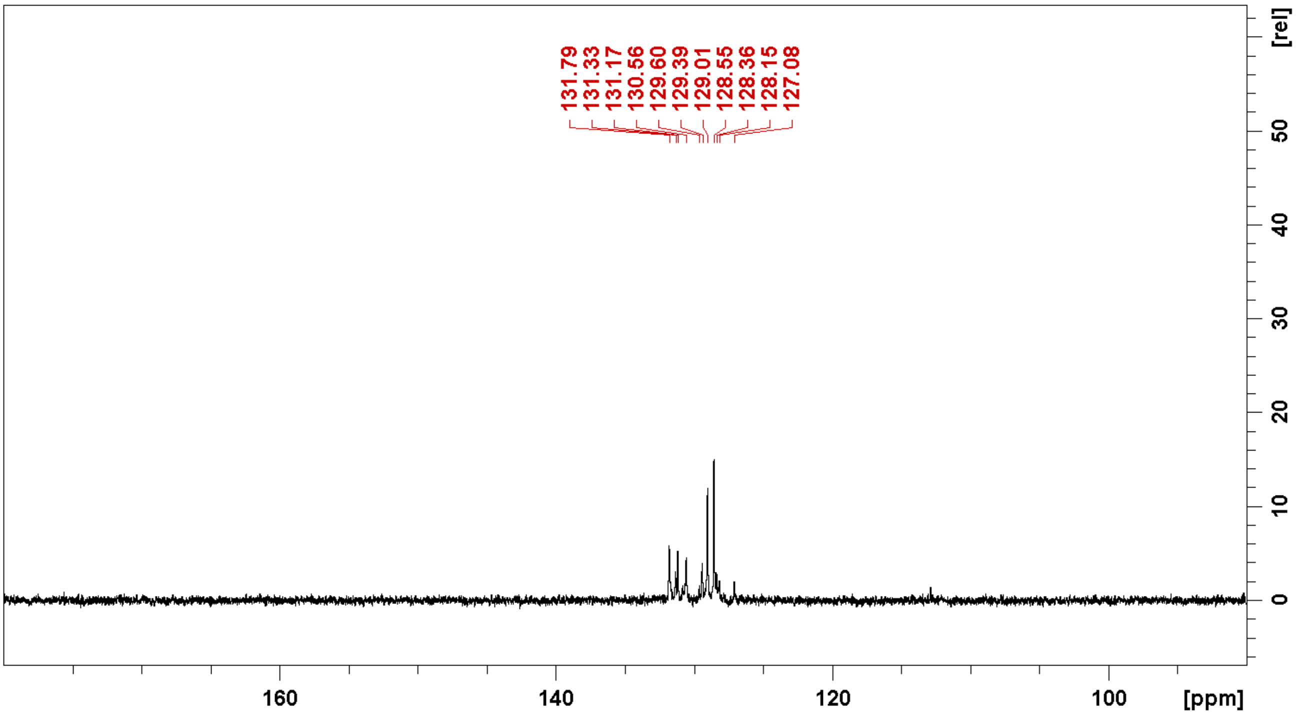

Figure S35. DEFT135 of **CN-C2C2** at 220 K

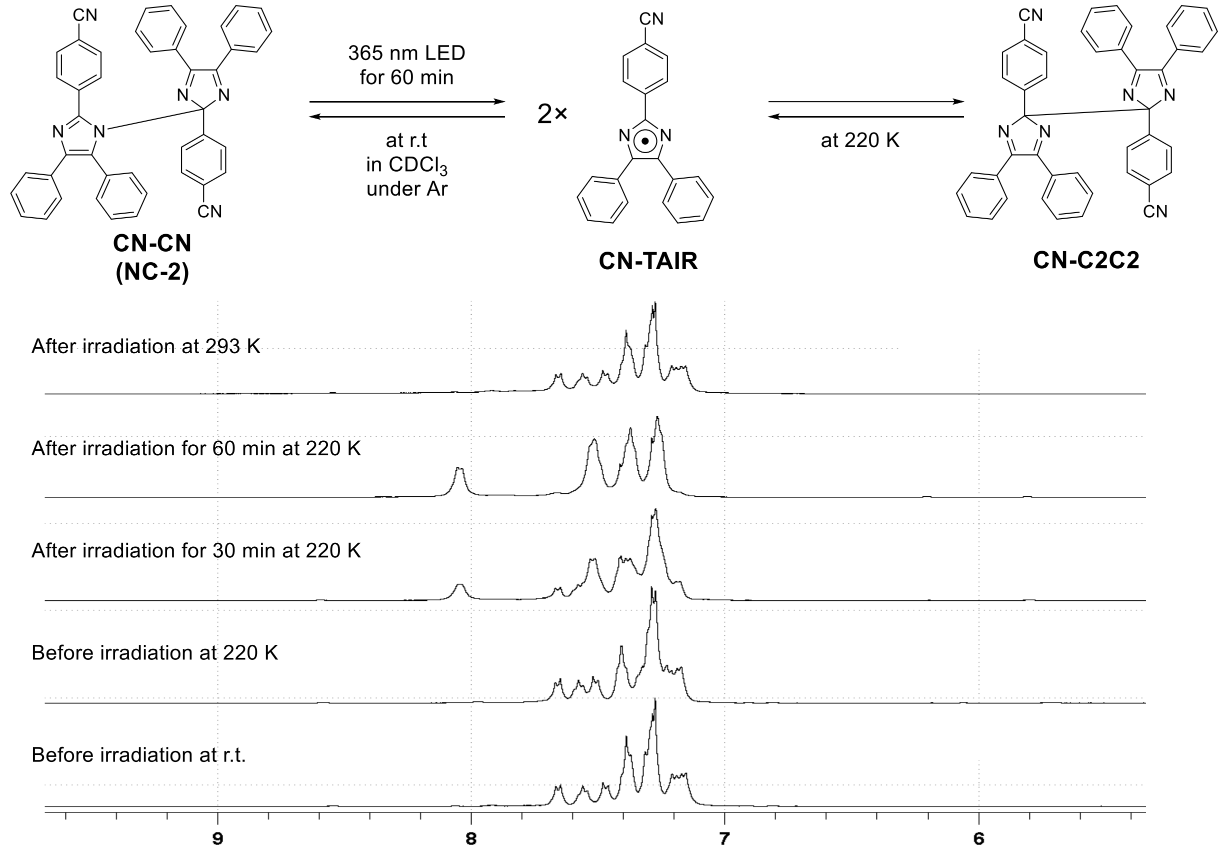

Figure S36. The ^{1}H NMR spectra of CN-CN(NC-2) before and after 60 minutes of photoirradiation at 220 K using a 365 nm LED in $CDCl_3$, along with the spectrum obtained after increasing from 220 K to 293 K.

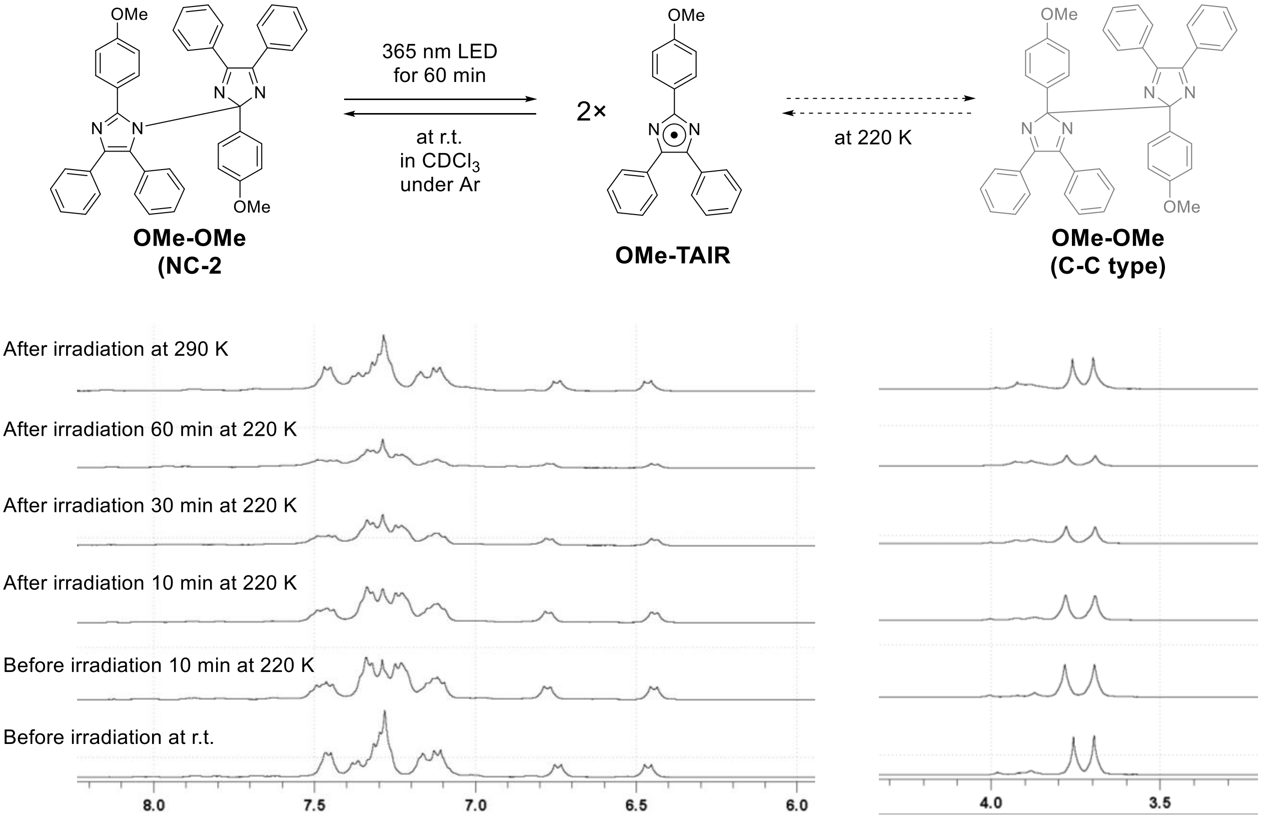

Figure S37. The ^{1}H NMR spectra of OMe-OMe (NC-2) before and after 60 minutes of photoirradiation at 220 K using a 365 nm LED in $CDCl_3$, along with the spectrum obtained after increasing from 220 K to 293 K.

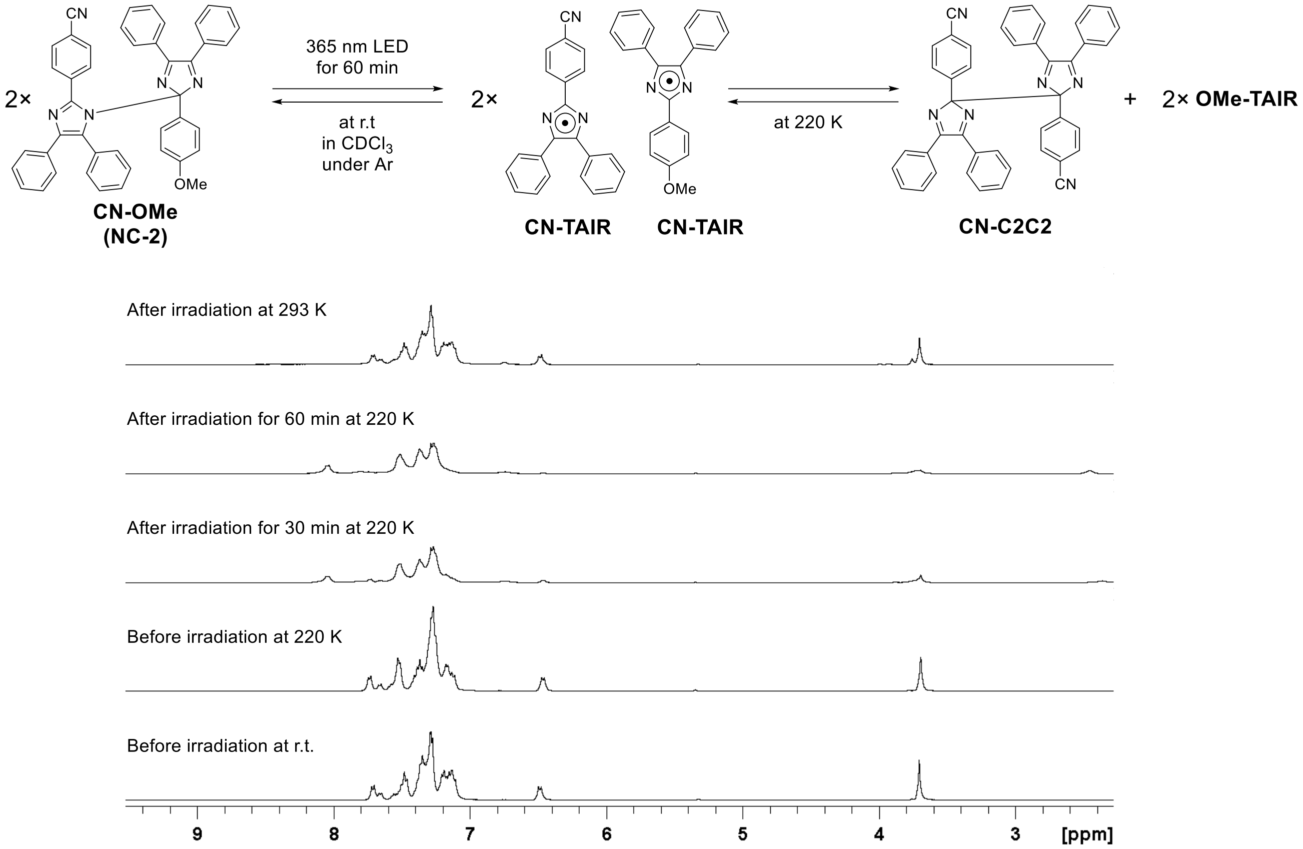

Figure S38. The ^{1}H NMR spectra of CN-OMe(NC-2) before and after 60 minutes of photoirradiation at 220 K using a 365 nm LED in $CDCl_3$, along with the spectrum obtained after increasing from 220 K to 293 K.

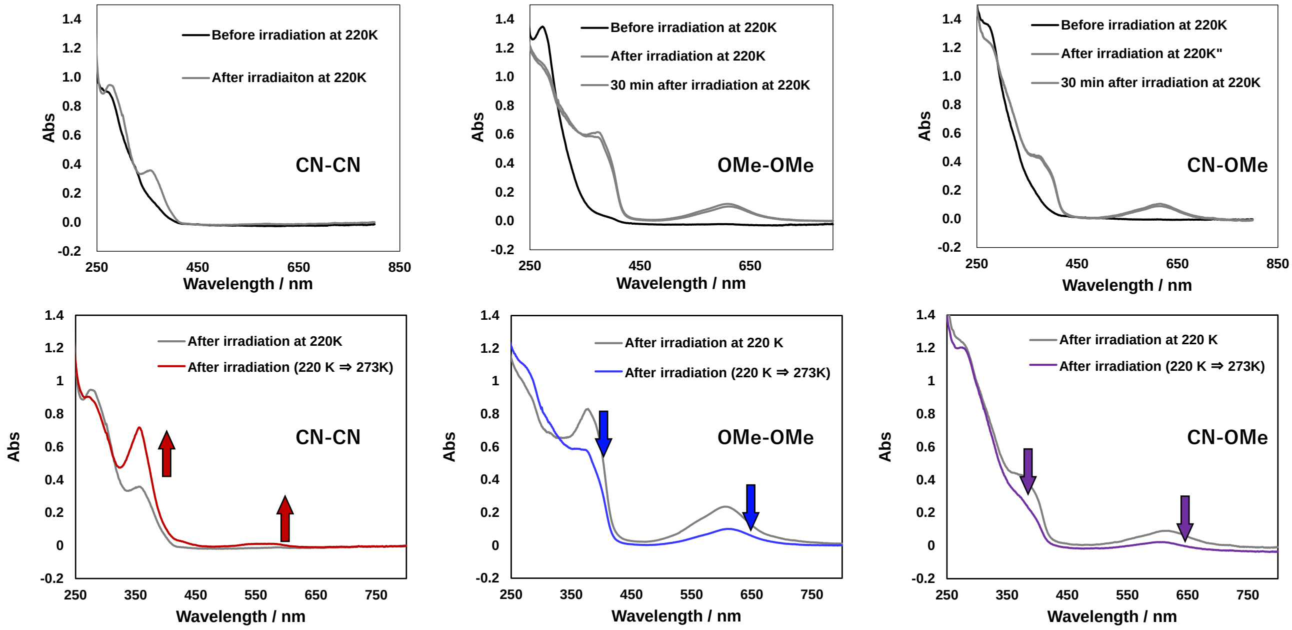

Figure S39. UV-vis absorption spectrum of CN-CN OMe-OMe and CN-OMe in degassed CH_2Cl_2 at 220 K. before and after photoirradiation (30 s with 365 nm LED). VT-UV-vis absorption spectra with increasing temperature from 220 K to 273 K. CN-TAIR is generated because the C-C type HABI (CN-C2C2) returns to the radicals at higher temperature. (Conc.; CN-CN = 1.0×10^{-5}, OMe-OMe = 1.3×10^{-5}, CN-OMe =1.1×10^{-5}.)

8 Simulated NMR Data

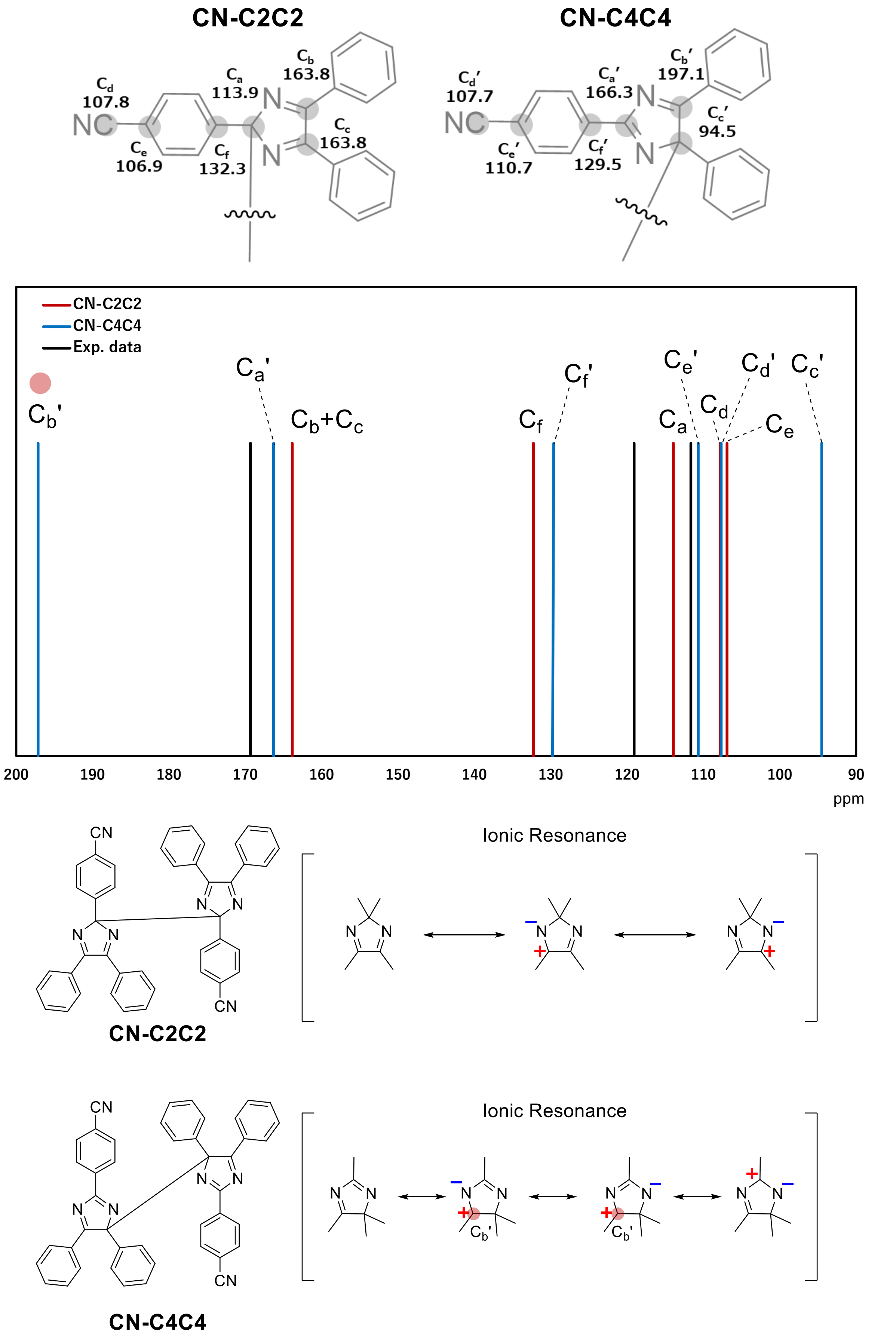

Figure S40. Comparison of calculated ^{13}C NMR data with experimental data and the ionic resonance structures of CN-C2C2 and CN-C4C4. The characteristic quaternary peaks ($C_a \sim C_f$, $C_a' \sim C_f'$) are pick up in the figure. ^{13}C NMR data are calculated by GIAO B3LYP/6-31G(d) [S3-S5].

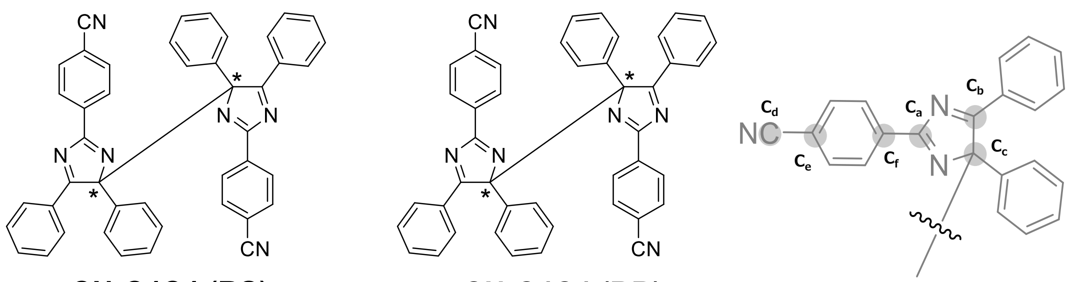

CN-C4C4 (RS) CN-C4C4 (RR)

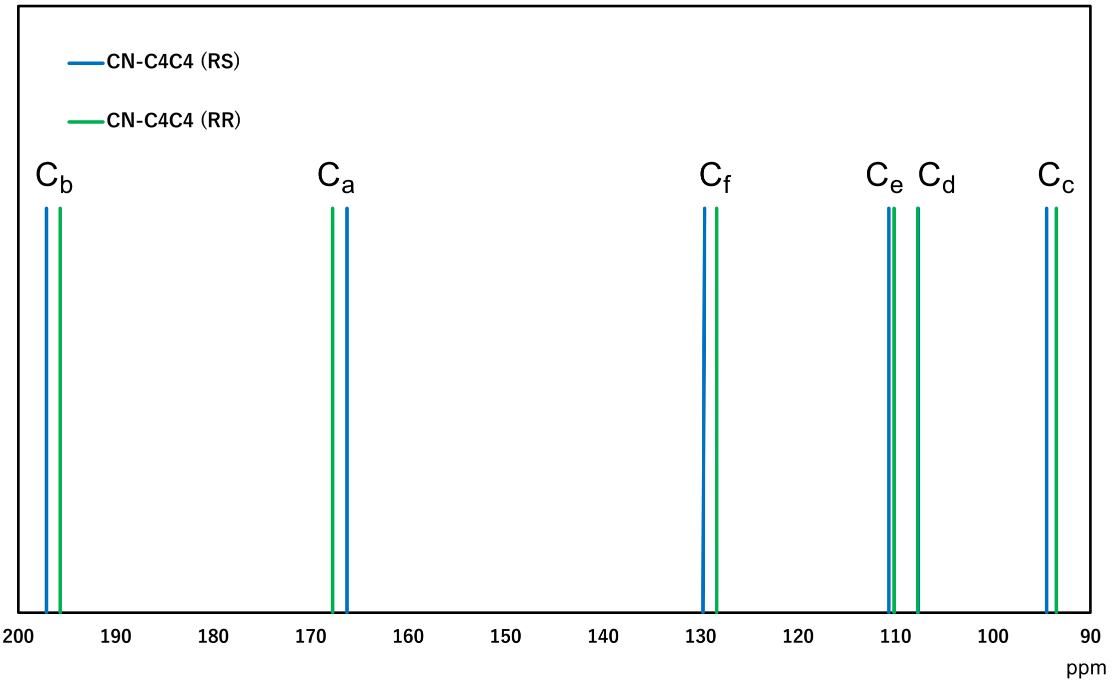

Figure S41. Comparison of calculated ^{13}C NMR data of CN-C4C4 (RS) and CN-C4C4 (RR). The characteristic quaternary peaks ($C_a \sim C_f$) are pick up in the figure. ^{13}C NMR data are calculated by GIAO B3LYP/6-31G(d).

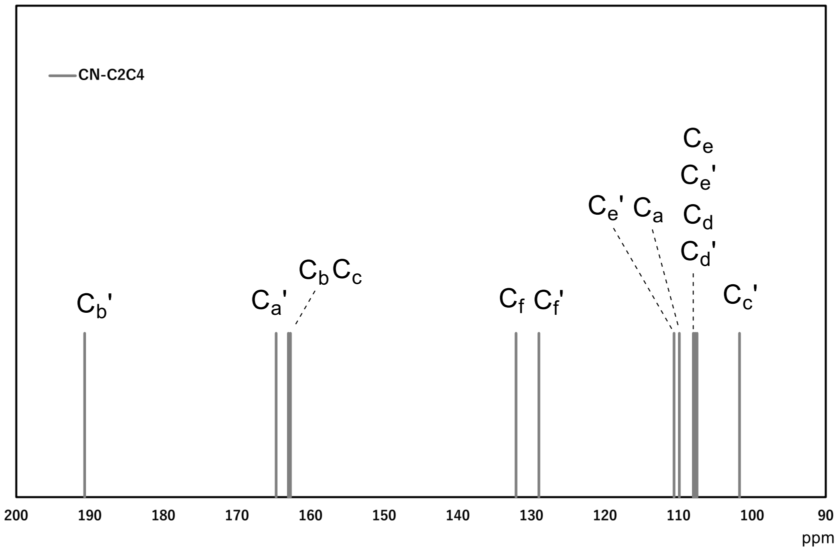

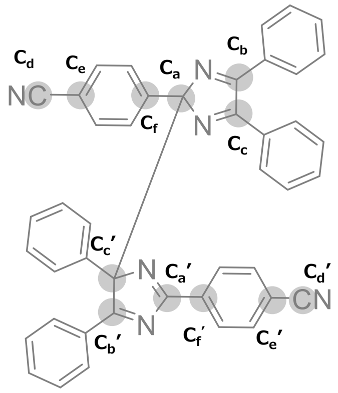

CN-C2C4

Figure S42. Comparison of calculated ^{13}C NMR data of CN-C2C4. The characteristic quaternary peaks ($C_a \sim C_f$) are pick up in the figure. ^{13}C NMR data are calculated by GIAO B3LYP/6-31G(d).

9. Isodesmic reaction

Table S4. Isodesmic reaction and spin density are calculated by DFT B3LYP-D3/6-31G(d) level of the theory. The relative stability of radicals was assessed by the enthalpy difference between the products and the reactants $\Delta(H_p^\circ - H_r^\circ)$.

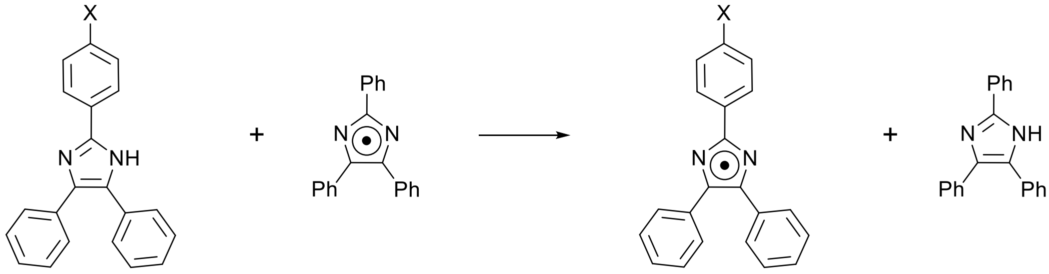

Substituents (-X)	$\Delta(H_p^\circ - H_r^\circ)$	Spin Density on N1	Spin Density on C2	Spin Density on C4
**CN**	+0.27	−0.077	+0.322	+0.260
**CF_3**	+0.38	−0.075	+0.323	+0.258
**COOMe**	+0.68	−0.073	+0.320	+0.263
**Cl**	−0.34	−0.069	+0.313	+0.259
**F**	−0.72	−0.066	+0.308	+0.261
**H**	0.00	−0.068	+0.314	+0.259
**Me**	−0.41	−0.064	+0.306	+0.257
**OMe**	−1.58	−0.057	+0.288	+0.250
**NH_2**	−2.98	−0.041	+0.254	+0.228
**NMe_2**	−2.65	−0.043	+0.258	+0.232

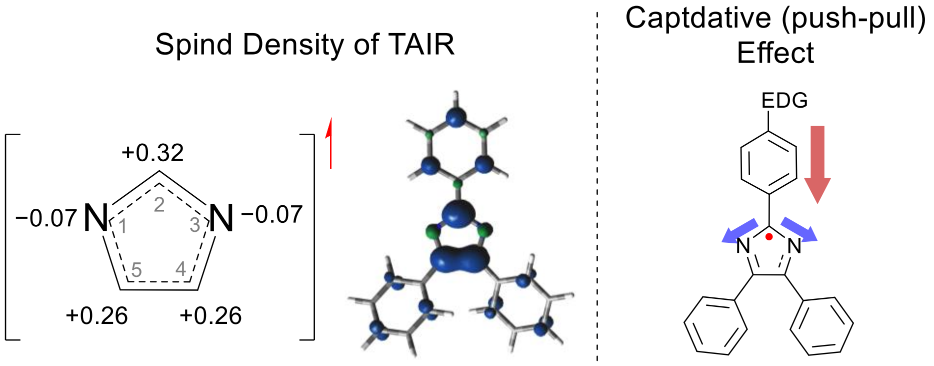

10. Transient-State and Energy Diagram

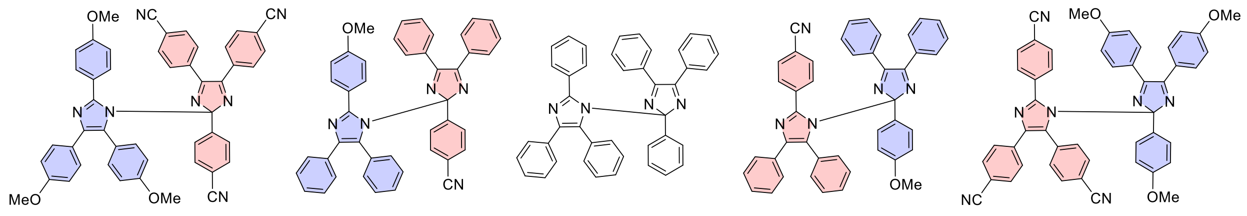

(OMe)$_3$-(CN)$_3$ OMe-CN H-H CN-OMe (CN)$_3$-(OMe)$_3$

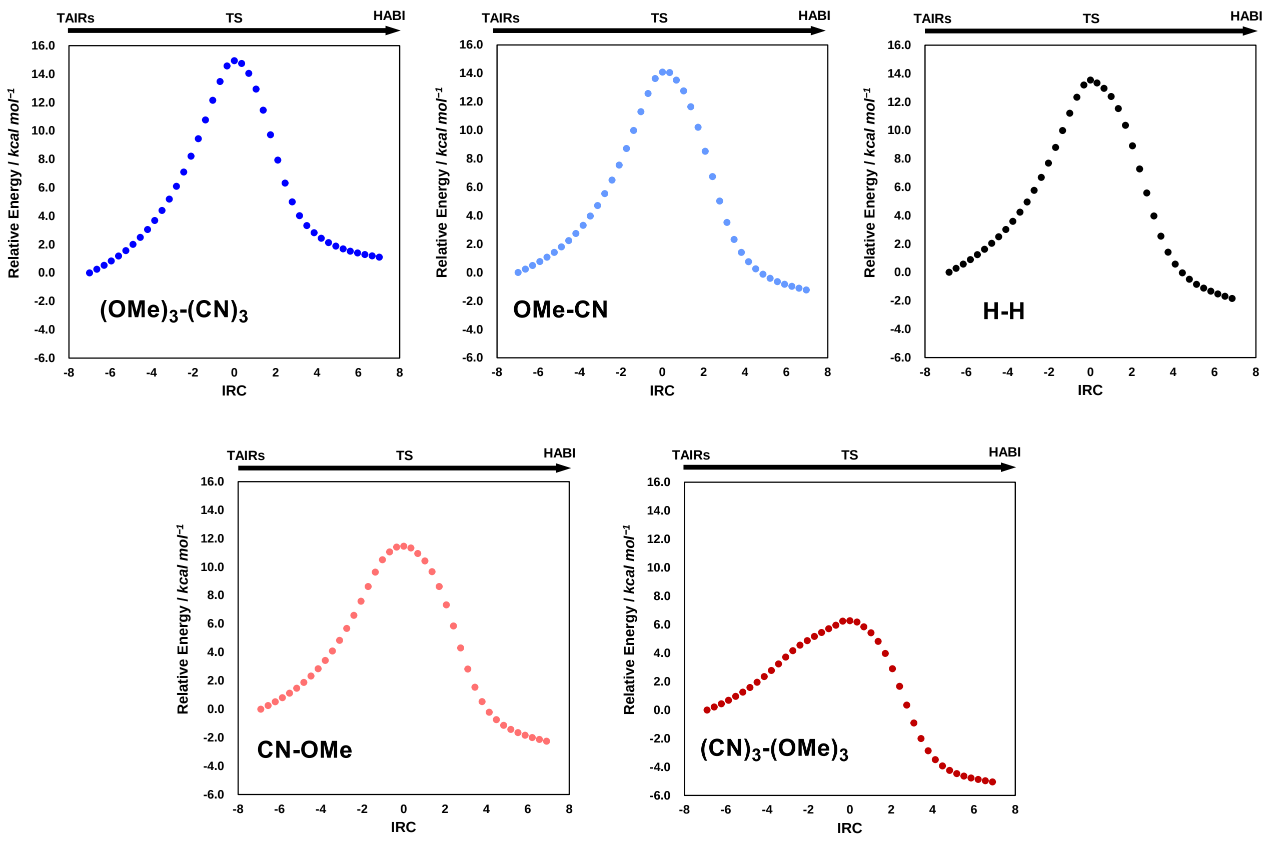

Figure S43. IRCs for each HABI ((OMe)$_3$-(CN)$_3$, OMe-CN, HPBI, CN-OMe and (CN)$_3$-(OMe)$_3$) formation calculated by DFT B3LYP-D3/6-31G(d) level of the theory.

Table S5. Energy value (kcal mal⁻¹) of each C-C type HABI isomer (C2C2, C2C4, C4C4 (RS), C4C4 (RR)) and transient sate for the radical-radical coupling (DFT B3LYP-D3/6-31G(d) level of the theory).
).

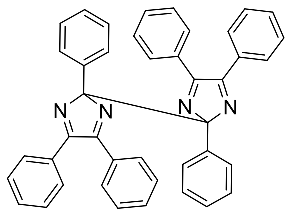

C2C2

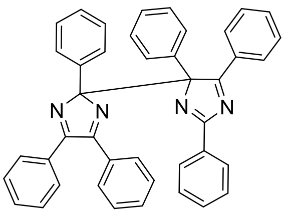

C2C4

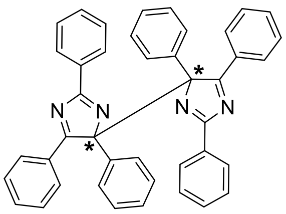

C4C4 (RS)

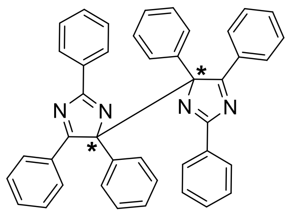

C4C4 (RR)

Entry	C-C type	2×TAIR	TS	HABI
**1 (H-H)**	C2C2	0.0	5.8	1.0
	C2C4	0.0	7.1	2.2
	C4C4 (RS)	0.0	9.1	1.2
	C4C4 (RR)	0.0	9.8	1.2
**2 (CN-CN)**	C2C2	0.0	3.1	−1.4
	C2C4	0.0	7.5	1.3
	C4C4 (RS)	0.0	8.3	1.2
	C4C4 (RR)	0.0	8.9	0.9
**3 (OMe-OMe)**	C2C2	0.0	5.7	3.0
	C2C4	0.0	9.4	4.3
	C4C4 (RS)	0.0	9.8	2.2
	C4C4 (RR)	0.0	10.4	1.8
**4 (CN-OMe)**	C2C2	0.0	3.8	0.6
	C2C4	0.0	6.8	1.6
	C4C2	0.0	8.0	2.9
	C4C4 (RS)	0.0	8.7	1.6
	C4C4 (RR)	0.0	8.9	1.0

(kcal mol^{-1})

11. NICS Computation

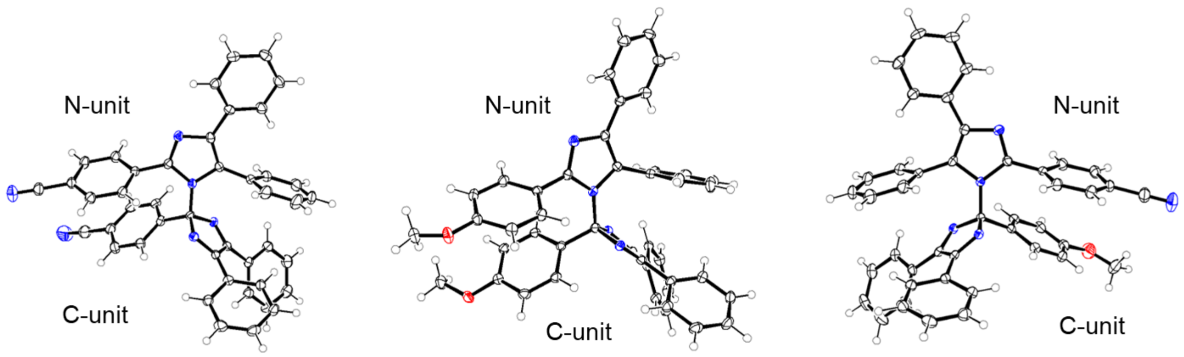

	NICS(1)$_{zz}$	
	**N-unit**	**C-unit**
**CN-CN**	−20.8	−8.9
**OMe-OMe**	−21.1	−9.0
**CN-OMe**	−20.3	−9.4

Figure S44. The NICS values of N-unit and C-unit of CN-CN, OMe-OMe and CN-OMe obtained from the single crystal XRD.

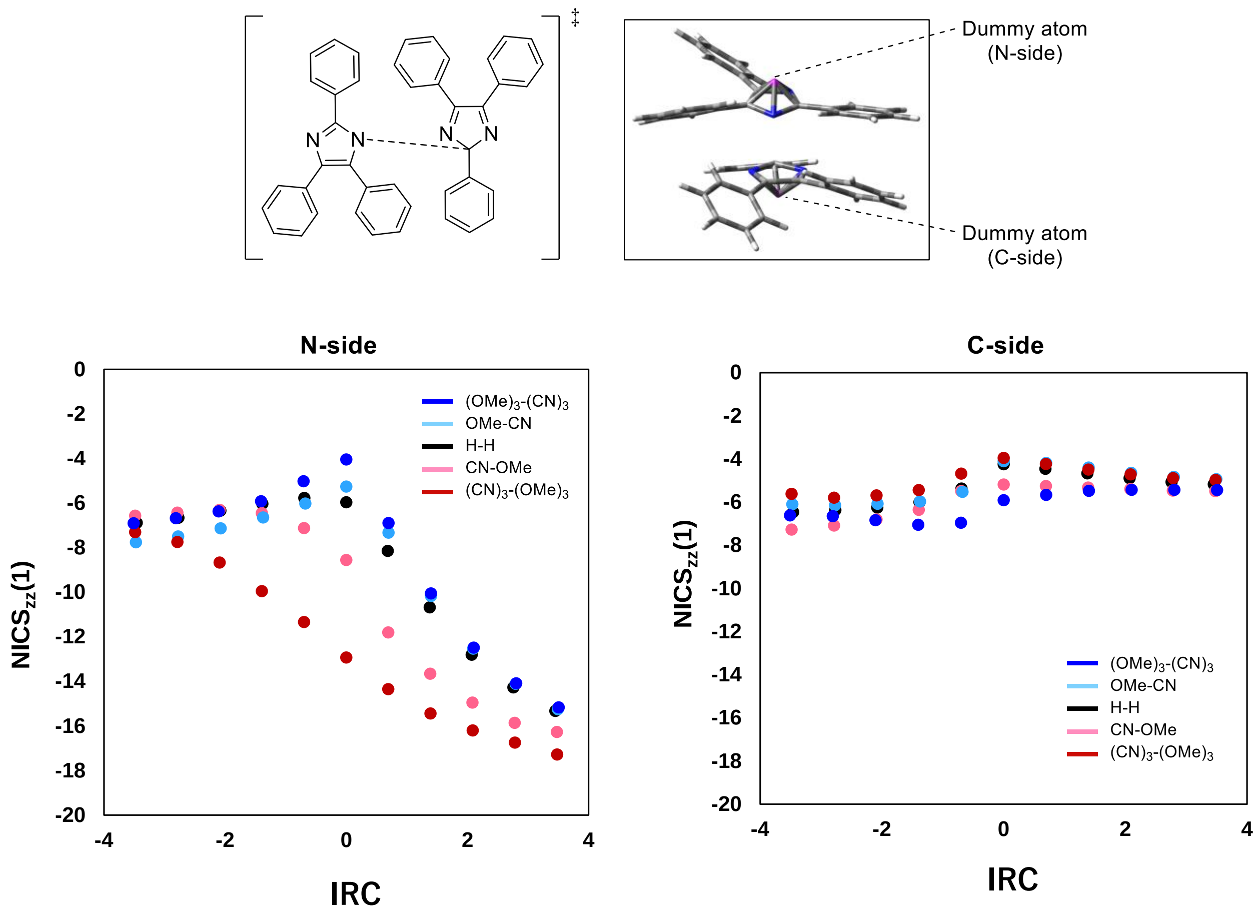

Figure S45. NICS(1)$_{zz}$ near the transition state (TS) for each HABI. The NICS(1)$_{zz}$ values were determined from the shielding effects at a position 1.0 Å above the center of the imidazole rings (N-side and C-side), using dummy atoms placed at the ring centers. The structures used were obtained from IRC calculations

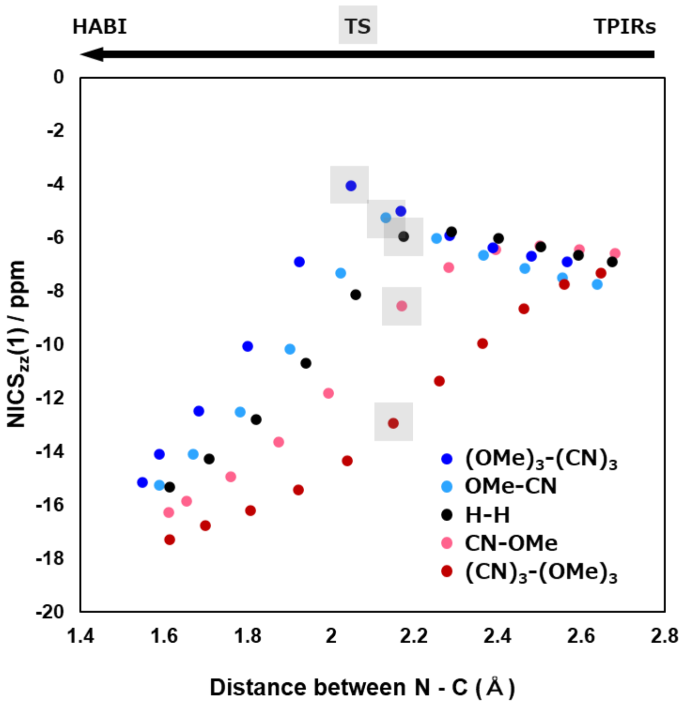

Figure S46. NICS(1)$_{zz}$ for the N–C bond transformation

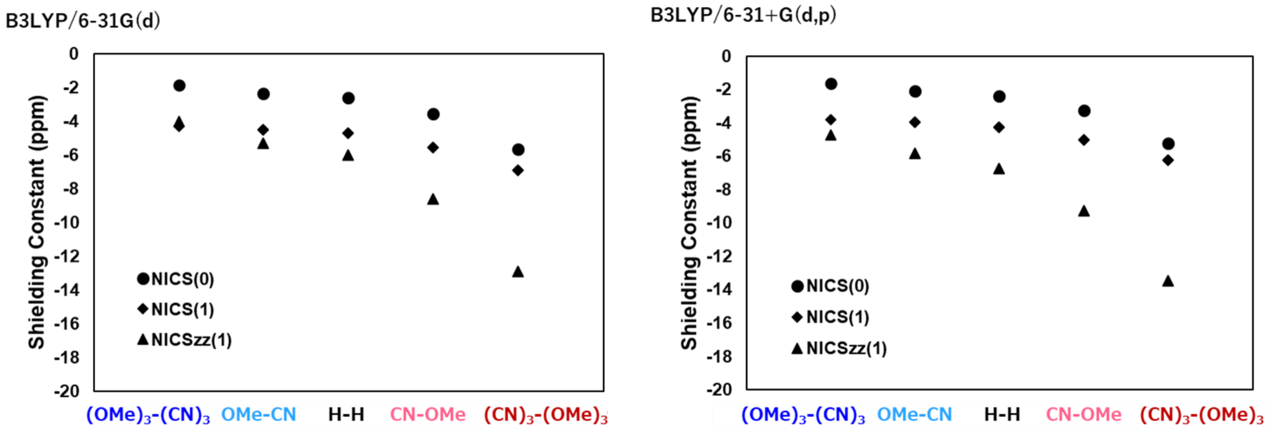

Figure S47. NICS(0), NICS(1) and NICS(1)$_{zz}$ of HABI derivatives on DFT B3LYP/6-31G(d) and B3LYP/6-31+G(d,p)

12. NBO Charge

Table S6. Total NBO charges on the N-side TAIR unit (N-unit) and C-side TAIR unit (C-unit) for the product (TAIR), transition state (TS), and product (HABI). Each structure optimized DFT B3LYP-D3/6-31G(d), and NBO charge simulated on DFT B3LYP/6-311+G(2d,p)[S6]

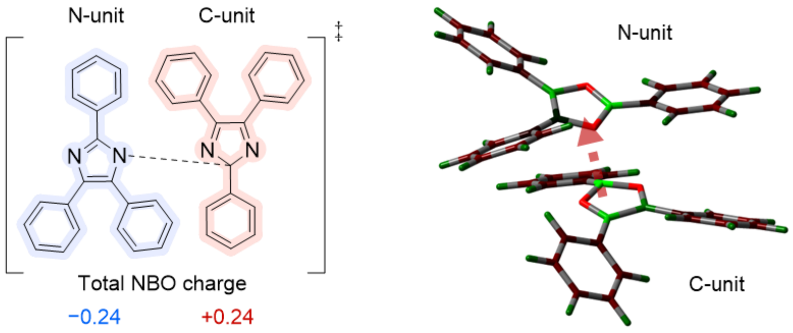

		N-unit			C-unit		
		**Reactant**	**TS**	**Product**	**Reactant**	**TS**	**Product**
**1**	**H-H**	0.00	-0.24	-0.22	0.00	0.24	0.22
**2**	**CN-OMe**	0.00	-0.37	-0.25	0.00	0.37	0.24
**3**	**OMe-CN**	0.00	-0.13	-0.22	0.00	0.13	0.21
**4**	**$(CN)_3$-$(OMe)_3$**	0.01	-0.57	-0.29	0.00	0.58	0.28
**5**	**$(OMe)_3$-$(CN)_3$**	0.00	0.00	-0.16	0.01	-0.01	0.16
**6**	**H-H (C2C2)**	0.00	0.00	0.00	0.00	0.00	0.00
**7**	**CN-OMe-C2C2**	0.00	-0.07	-0.03	0.00	+0.07	+0.03
**8**	**$(CN)_3$-$(OMe)_3$-C2C2**	0.00	-0.14	-0.07	0.00	+0.13	+0.05

Table S7. Orbital interactions were simulated using second-order perturbation theory in NBO[S3]. $\Delta E_{AB}(2)$ represents the interaction energy between donor and acceptor orbitals. The $\sigma \Rightarrow \pi^*$ interaction involves donation from the σ (N–C) bond to the π* orbital, where π_a is the N=C anti-bonding orbital and π_b is the C=C anti-bonding orbital in the imidazole ring. The interaction between σ and π* in the products and reactants could not be obtained from the calculations. Before transition state (TS), the distance between the two TAIR units is large, resulting in weak σ-bond character. After the TS, the N–C bond in HABI does not participate in the π-conjugation of the imidazole ring, leading to a small interaction energy.

Entry		Reactant		TS		Product	
		$\Delta E_{AB}(2)$ $\sigma \Rightarrow \pi_a^*$	$\Delta E_{AB}(2)$ $\sigma \Rightarrow \pi_b^*$	$\Delta E_{AB}(2)$ $\sigma \Rightarrow \pi_a^*$	$\Delta E_{AB}(2)$ $\sigma \Rightarrow \pi_b^*$	$\Delta E_{AB}(2)$ $\sigma \Rightarrow \pi_a^*$	$\Delta E_{AB}(2)$ $\sigma \Rightarrow \pi_b^*$
1	$(OMe)_3$-$(CN)_3$	n.d.	n.d.	9.09	7.54	n.d.	n.d.
2	OMe-CN	n.d.	n.d.	11.60	8.84	n.d.	n.d.
3	H-H	n.d.	n.d.	12.73	9.21	n.d.	n.d.
4	CN-OMe	n.d.	n.d.	13.09	9.19	n.d.	n.d.
5	$(CN)_3$-$(OMe)_3$	n.d.	n.d.	13.76	9.41	n.d.	n.d.

Table S8. The $LP \Rightarrow \pi^*$ interaction involves donation from the LP of N to the π* orbital, The interaction between LP and π* in the product could not be obtained from the calculations because the LP in TAIR does not participate

		Reactant		TS		Product	
		$\Delta E_{AB}(2)$ $LP \Rightarrow \pi_a^*$	$\Delta E_{AB}(2)$ $LP \Rightarrow \pi_b^*$	$\Delta E_{AB}(2)$ $LP \Rightarrow \pi_a^*$	$\Delta E_{AB}(2)$ $LP \Rightarrow \pi_b^*$	$\Delta E_{AB}(2)$ $LP \Rightarrow \pi_a^*$	$\Delta E_{AB}(2)$ $LP \Rightarrow \pi_b^*$
1	$(OMe)_3$-$(CN)_3$	n.d.	n.d.	2.50	2.30	39.81	23.98
2	OMe-CN	n.d.	n.d.	2.76	2.27	42.35	24.88
3	H-H	n.d.	n.d.	2.88	2.30	42.27	23.98
4	CN-OMe	n.d.	n.d.	3.05	2.15	42.68	25.55
5	$(CN)_3$-$(OMe)_3$	n.d.	n.d.	2.92	2.02	40.17	26.71

13. Hammett plot

Table S9. The activation free energy (Δ‡G°) for each HABI group (X-CN, X-H, X-OMe, Cn-X, H-X, OMe-X) with X substituents (Entry 1~10) determined as the energy gap between TAIR and the TS by theoretical calculation.

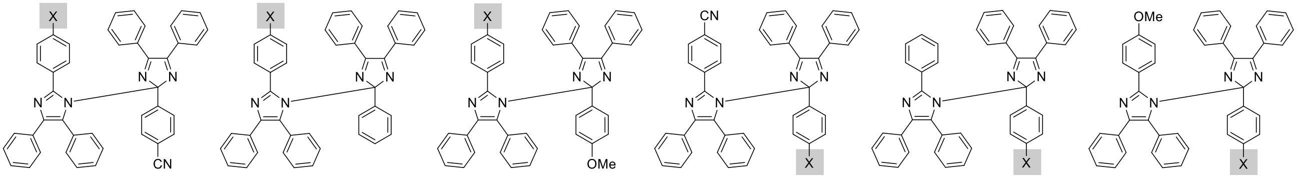

	Entry	σp+	σp	X-CN $\Delta^{\ddagger}G^{\circ}$	X-H $\Delta^{\ddagger}G^{\circ}$	X-OMe $\Delta^{\ddagger}G^{\circ}$	CN-X $\Delta^{\ddagger}G^{\circ}$	H-X $\Delta^{\ddagger}G^{\circ}$	OMe-X $\Delta^{\ddagger}G^{\circ}$
1	–CN	0.73	0.66	14.0	13.5	12.7	14.0	15.4	15.9
2	–CF3	0.66	0.54	13.6	13.6	12.6	13.1	15.1	15.7
3	–COOMe	0.49	0.45	13.9	13.8	12.9	13.6	15.8	16.3
4	–Cl	0.11	0.23	14.9	14.9	13.9	13.6	15.0	16.1
5	–H	0	0	15.4	15.6	15.4	13.5	15.6	16.5
6	–F	-0.07	0.06	15.3	15.5	14.6	13.5	15.0	16.3
7	–Me	-0.31	-0.07	14.8	15.0	14.4	12.5	14.9	15.7
8	–OMe	-0.78	-0.27	15.9	16.5	15.6	12.7	15.4	15.6
9	–NH2	-1.3	-0.66	16.9	17.5	16.8	12.2	15.0	16.2
10	–NMe2	-1.7	-0.83	16.4	17.5	17.2	12.3	15.1	16.3

$$k(T) = \frac{k_B T}{hc^{\circ}} e^{-\Delta^{\ddagger}G^{\circ}/RT} \quad \text{(Eq3)}$$

k_B; Boltzmann constant, 1.38064852×10^{-23} $(J \times K^{-1})$
h ; Planck constant, 6.62607004×10^{-34} $(J \times s)$
c°; concentration (set to 1 in this case)
R ; the gas constant, 8.3144598 $(J \times K^{-1} \times mol^{-1})$
T ; temperature (K)
$\Delta^{\ddagger}G^{\circ}$; free energy of activation $(kJ \times mol^{-1})$

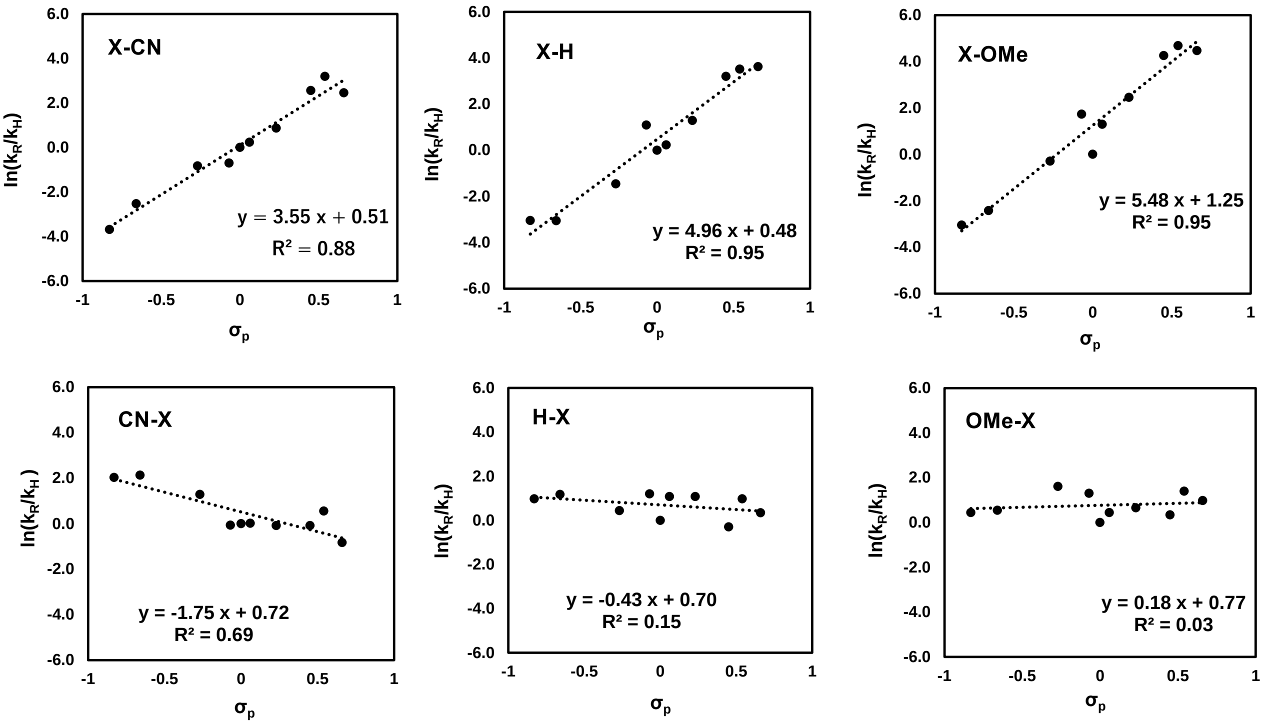

Figure S48. Rate constant (k) is defined in equation 3. ^{e} k_R / k_H is represented the rate constant for each HABI derivative with X ≠ H divided by the rate constant for X = H.

14. Theoretical calculations

Quantum chemical computations in gas phase have been performed with the Gaussian 16 (Revision C.02) suite of programs. The cartesian coordinates (in Å) is computed geometries at (R,U)B3LYP-D3/6-31G(d)[S3-S4] level of theory are listed in following. The energy minimum structures and transition state structures were confirmed by vibrational frequency analysis. The transition states were further confirmed by intrinsic reaction coordinate (IRC) calculation with the alternative local quadratic approximation (LQA) approach.

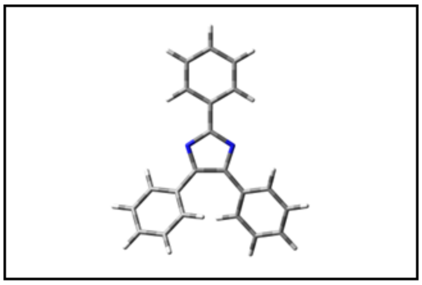

TPIR

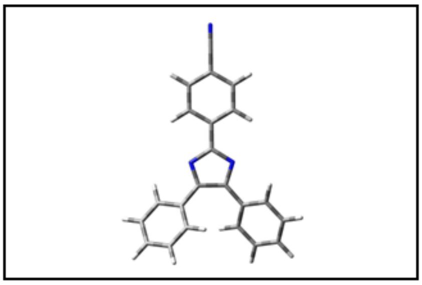

CN-TAIR

Tag	Symbol	X	Y	Z
1	C	-1.662249	0.000000	0.000000
2	C	0.339596	-0.742456	-0.010959
3	C	0.339596	0.742456	0.010959
4	N	-0.925698	1.154403	0.042314
5	N	-0.925697	-1.154403	-0.042314
6	C	1.445113	-1.695396	0.059721
7	C	1.287356	-2.974209	-0.512223
8	C	2.642833	-1.402395	0.740710
9	C	2.307996	-3.915197	-0.432079
10	H	0.355096	-3.204346	-1.017026
11	C	3.658140	-2.352209	0.826324
12	H	2.765207	-0.437525	1.220190
13	C	3.498892	-3.607581	0.234741
14	H	2.177061	-4.892722	-0.888284
15	H	4.572570	-2.113748	1.362568
16	C	1.445113	1.695397	-0.059721
17	C	1.287354	2.974211	0.512219
18	C	2.642834	1.402394	-0.740707
19	C	2.307994	3.915199	0.432076
20	H	0.355093	3.204349	1.017020
21	C	3.658141	2.352208	-0.826321
22	H	2.765210	0.437523	-1.220185
23	C	3.498891	3.607582	-0.234741
24	H	2.177057	4.892725	0.888278
25	H	4.572572	2.113747	-1.362563
26	C	-3.108660	0.000000	0.000000
27	C	-3.821831	-1.216807	-0.045815
28	C	-3.821831	1.216807	0.045815
29	C	-5.211586	-1.211768	-0.045620
30	C	-5.211586	1.211767	0.045620
31	H	-3.262788	2.145739	0.078004
32	C	-5.911194	-0.000001	0.000000
33	H	-5.755512	-2.151793	-0.080178
34	H	-5.755513	2.151791	0.080179
35	H	-3.262787	-2.145739	-0.078003
36	H	4.294510	4.345019	-0.298111
37	H	4.294511	-4.345018	0.298112
38	H	-6.997990	-0.000001	0.000000

Tag	Symbol	X	Y	Z
1	C	-1.081530	0.000002	0.000001
2	C	0.917802	-0.743915	-0.012720
3	C	0.917804	0.743916	0.012721
4	N	-0.347980	1.154613	0.046626
5	N	-0.347979	-1.154612	-0.046624
6	C	2.022364	-1.695458	0.058431
7	C	1.863853	-2.975508	-0.511641
8	C	3.220030	-1.400579	0.739864
9	C	2.884502	-3.915737	-0.430102
10	C	4.234543	-2.350503	0.827729
11	H	3.341701	-0.435591	1.219069
12	C	4.074876	-3.606505	0.237338
13	H	2.754494	-4.893764	-0.885126
14	H	5.148435	-2.112201	1.364677
15	C	2.022364	1.695458	-0.058432
16	C	1.863843	2.975517	0.511620
17	C	3.220040	1.400572	-0.739845
18	C	2.884492	3.915746	0.430080
19	C	4.234553	2.350497	-0.827709
20	H	3.341719	0.435578	-1.219036
21	C	4.074876	3.606506	-0.237339
22	H	2.754475	4.893780	0.885087
23	H	5.148451	2.112188	-1.364642
24	C	-2.528415	0.000001	0.000001
25	C	-3.240964	-1.217074	-0.049031
26	C	-3.240966	1.217074	0.049032
27	C	-4.626736	-1.218979	-0.049086
28	C	-4.626739	1.218977	0.049087
29	H	-2.683121	2.146180	0.083644
30	C	-5.330914	-0.000002	0.000000
31	H	-5.177086	-2.153524	-0.085899
32	H	-5.177090	2.153521	0.085900
33	H	-2.683117	-2.146178	-0.083643
34	H	0.932254	-3.206459	-1.017209
35	H	0.932237	3.206474	1.017171
36	H	4.870251	-4.343935	0.302247
37	C	-6.762542	-0.000003	0.000000
38	N	-7.926423	-0.000004	0.000000
39	H	4.870250	4.343937	-0.302248

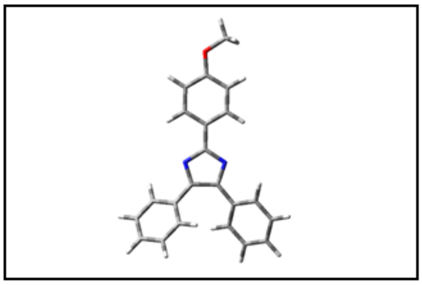

OMe-TAIR

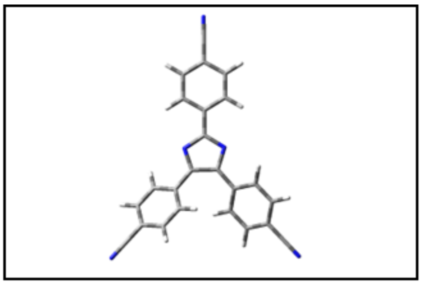

$(CN)_3$-TAIR

Tag	Symbol	X	Y	Z
1	C	-0.934355	-0.098741	-0.003474
2	C	1.034064	0.729679	0.007232
3	C	1.099245	-0.750288	-0.009674
4	N	-0.146876	-1.219395	-0.039531
5	N	-0.247988	1.087631	0.034133
6	C	2.098431	1.730660	-0.061721
7	C	1.888207	2.999010	0.515330
8	C	3.306380	1.491900	-0.744969
9	C	2.867564	3.983454	0.436911
10	C	4.280758	2.484132	-0.828039
11	H	3.469511	0.534141	-1.226724
12	C	4.069484	3.729704	-0.232307
13	H	2.695557	4.952886	0.897014
14	H	5.204060	2.286022	-1.365693
15	C	2.246700	-1.654129	0.062133
16	C	2.147795	-2.937707	-0.511369
17	C	3.429159	-1.309218	0.744794
18	C	3.209198	-3.832766	-0.430334
19	C	4.486212	-2.212551	0.830507
20	H	3.507990	-0.339860	1.224462
21	C	4.384356	-3.473385	0.238080
22	H	3.122269	-4.814821	-0.887555
23	H	5.388563	-1.933429	1.367779
24	C	-2.373224	-0.162318	-0.004921
25	C	-3.145451	1.016027	0.036897
26	C	-3.042882	-1.408508	-0.048403
27	C	-4.533540	0.965283	0.035765
28	C	-4.422460	-1.466592	-0.049623
29	H	-2.449184	-2.315660	-0.077978
30	C	-5.181858	-0.280909	-0.007627
31	H	-5.100766	1.888133	0.067331
32	H	-4.951074	-2.413929	-0.081532
33	H	-2.631517	1.970779	0.067443
34	H	0.947826	3.187426	1.022518
35	H	1.227386	-3.208869	-1.017670
36	H	4.833049	4.500512	-0.293846
37	H	5.212106	-4.174620	0.301781
38	O	-6.528842	-0.451033	-0.012935
39	C	-7.362859	0.700389	0.029071
40	H	-8.387660	0.325633	0.016905
41	H	-7.198390	1.281868	0.945590
42	H	-7.200487	1.345652	-0.844104

Tag	Symbol	X	Y	Z
1	C	-1.789757	0.000000	0.000000
2	C	0.210325	-0.742567	-0.036601
3	C	0.210325	0.742567	0.036601
4	N	-1.053268	1.152527	0.082643
5	N	-1.053268	-1.152527	-0.082643
6	C	1.313137	-1.698197	0.005163
7	C	1.150284	-2.959489	-0.604688
8	C	2.511914	-1.426640	0.694458
9	C	2.162798	-3.905244	-0.558079
10	C	3.526215	-2.373223	0.753621
11	H	2.637880	-0.479354	1.206050
12	C	3.362272	-3.616754	0.119742
13	H	2.038192	-4.869261	-1.040277
14	H	4.443067	-2.161743	1.293868
15	C	1.313137	1.698197	-0.005164
16	C	1.150283	2.959490	0.604686
17	C	2.511915	1.426639	-0.694456
18	C	2.162797	3.905245	0.558077
19	C	3.526216	2.373222	-0.753620
20	H	2.637882	0.479353	-1.206048
21	C	3.362272	3.616754	-0.119742
22	H	2.038191	4.869262	1.040274
23	H	4.443068	2.161742	-1.293865
24	C	-3.233872	0.000000	0.000000
25	C	-3.945838	-1.216463	-0.085423
26	C	-3.945838	1.216463	0.085423
27	C	-5.331110	-1.217957	-0.085428
28	C	-5.331111	1.217956	0.085428
29	H	-3.388994	2.144632	0.147652
30	C	-6.034145	0.000000	0.000000
31	H	-5.882044	-2.150449	-0.149954
32	H	-5.882045	2.150448	0.149954
33	H	-3.388994	-2.144632	-0.147652
34	H	0.218225	-3.174644	-1.115322
35	H	0.218223	3.174645	1.115318
36	C	4.411529	4.590640	-0.168783
37	N	5.264415	5.380919	-0.206714
38	C	4.411529	-4.590640	0.168784
39	N	5.264415	-5.380919	0.206714
40	C	-7.465747	0.000000	0.000000
41	N	-8.629415	0.000000	0.000000

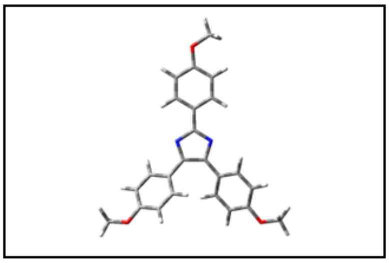

$(OMe)_3$-TAIR

Tag	Symbol	X	Y	Z
1	C	-1.752753	-0.143199	-0.004798
2	C	0.207741	0.704667	0.009000
3	C	0.288036	-0.773107	-0.014273
4	N	-0.956380	-1.254596	-0.048909
5	N	-1.080998	1.048757	0.040751
6	C	1.257500	1.714957	-0.058152
7	C	1.027864	2.988938	0.493054
8	C	2.483187	1.496807	-0.724424
9	C	1.983886	3.997082	0.422015
10	C	3.439035	2.495840	-0.809024
11	H	2.673078	0.540373	-1.198621
12	C	3.202485	3.752874	-0.229714
13	H	1.774166	4.960617	0.871923
14	H	4.377859	2.334910	-1.329224
15	C	1.440116	-1.664127	0.055799
16	C	1.348394	-2.957388	-0.491185
17	C	2.635445	-1.313597	0.720915
18	C	2.407093	-3.856717	-0.417464
19	C	3.692960	-2.203954	0.808155
20	H	2.721475	-0.341101	1.192581
21	C	3.592618	-3.480968	0.232805
22	H	2.301867	-4.838731	-0.863946
23	H	4.609076	-1.941702	1.327624
24	C	-3.194381	-0.221822	-0.006149
25	C	-3.978889	0.946312	0.045319

Tag	Symbol	X	Y	Z
26	C	-3.850873	-1.473250	-0.059217
27	C	-5.367468	0.881017	0.044421
28	C	-5.230728	-1.546501	-0.060337
29	H	-3.247719	-2.373957	-0.096137
30	C	-6.002618	-0.370518	-0.008587
31	H	-5.943916	1.797978	0.083576
32	H	-5.748560	-2.499624	-0.099544
33	H	-3.475425	1.906446	0.083096
34	H	0.079044	3.174680	0.985285
35	H	0.424801	-3.245596	-0.982028
36	O	-7.350031	-0.554994	-0.014660
37	C	-8.192637	0.588286	0.036989
38	H	-9.214884	0.206128	0.022456
39	H	-8.032888	1.164782	0.957819
40	H	-8.036656	1.242822	-0.830785
41	O	4.208953	4.660235	-0.360234
42	C	4.024917	5.958201	0.187344
43	H	3.879684	5.920185	1.275321
44	H	4.940720	6.508699	-0.035373
45	H	3.170671	6.472506	-0.272884
46	O	4.690599	-4.274544	0.365650
47	C	4.646733	-5.586887	-0.177098
48	H	4.498109	-5.568624	-1.265108
49	H	5.616343	-6.035100	0.047161
50	H	3.852649	-6.188069	0.285505

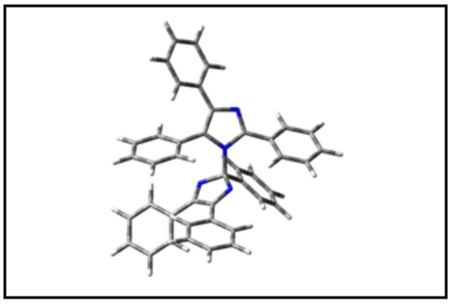

H-H

Tag	Symbol	X	Y	Z	Tag	Symbol	X	Y	Z
1	N	-0.900871	1.296888	-0.799093	39	H	-5.562270	-1.126664	-2.992074
2	N	1.193336	0.300347	-0.151051	40	C	0.791884	4.209135	3.042411
3	N	-0.814406	0.009255	1.201137	41	C	3.930487	4.386117	-0.222578
4	N	3.396332	0.087777	-0.449432	42	H	4.778442	4.875256	0.249535
5	C	-2.040293	0.733238	-0.593212	43	C	-4.331176	2.034308	-3.289190
6	C	-3.138818	0.803936	-1.573234	44	H	-4.479284	2.947925	-3.858129
7	C	2.594454	2.394101	-0.576876	45	C	0.524784	-3.166869	0.814235
8	C	0.247557	2.122374	1.265258	46	H	1.299161	-3.127791	1.573854
9	C	2.406587	0.950182	-0.357251	47	C	5.425939	-4.582756	-0.667542
10	C	1.473337	-1.074509	-0.166310	48	H	6.088954	-5.440738	-0.742999
11	C	-0.051021	0.931380	0.341374	49	C	4.102628	-4.674775	-1.103577
12	C	2.844023	-1.171070	-0.344564	50	H	3.732640	-5.603568	-1.530655
13	C	-3.107239	-0.743531	1.377504	51	C	1.997076	4.473709	-1.668438
14	C	-2.002672	-0.037261	0.707740	52	H	1.339633	5.029379	-2.332324
15	C	1.032281	1.891357	2.401150	53	C	-5.140059	0.919775	-3.529521
16	H	1.424931	0.895905	2.582590	54	H	-5.919096	0.966470	-4.285576
17	C	3.703494	-2.362423	-0.464621	55	C	-0.005950	4.433304	1.920744
18	C	0.460685	-2.143641	-0.143540	56	H	-0.404198	5.424721	1.723133
19	C	-3.952074	-0.313816	-1.821764	57	C	5.042672	-2.275325	-0.046634
20	H	-3.795140	-1.228495	-1.260193	58	H	5.403810	-1.331018	0.347997
21	C	-3.330159	1.975142	-2.323006	59	C	-0.532080	-2.205962	-1.134735
22	H	-2.685842	2.828719	-2.137387	60	H	-0.576404	-1.426372	-1.889404
23	C	-0.280802	3.391681	1.032978	61	C	-3.854194	-2.573020	2.780377
24	H	-0.873990	3.572409	0.144393	62	H	-3.632086	-3.477515	3.340019
25	C	3.692690	3.034425	0.014934	63	C	-0.383414	-4.225320	0.783114
26	H	4.348255	2.458959	0.660444	64	H	-0.317399	-5.012802	1.529232
27	C	1.310079	2.933262	3.281490	65	C	-5.444119	-0.924025	2.003434
28	H	1.928418	2.749411	4.155779	66	H	-6.457945	-0.535396	1.965442
29	C	3.081396	5.111397	-1.062566	67	C	-1.380217	-4.268297	-0.194375
30	C	3.249812	-3.575872	-1.008968	68	H	-2.094051	-5.087186	-0.211632
31	H	2.231865	-3.656114	-1.373383	69	C	5.892922	-3.374581	-0.144281
32	C	1.753407	3.121879	-1.431688	70	H	6.924204	-3.287520	0.188660
33	H	0.906729	2.626692	-1.894882	71	C	-5.163686	-2.086133	2.724795
34	C	-4.423147	-0.256979	1.327608	72	H	-5.962093	-2.609980	3.243603
35	H	-4.645535	0.645990	0.768631	73	C	-1.453440	-3.252190	-1.151131
36	C	-2.829120	-1.902419	2.119774	74	H	-2.219038	-3.281447	-1.922230
37	H	-1.810472	-2.270865	2.152098	75	H	3.267415	6.166335	-1.246829
38	C	-4.943329	-0.254692	-2.800187	76	H	1.012547	5.024033	3.726578

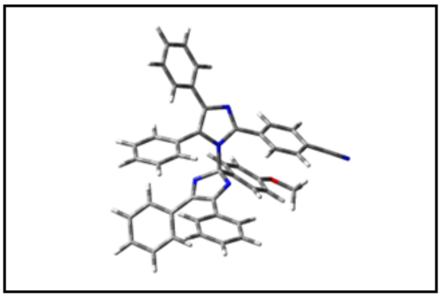

CN-OMe

Tag	Symbol	X	Y	Z
1	N	0.046119	-1.265409	-0.978678
2	N	-0.304455	1.001265	-0.252346
3	N	0.896341	-0.599383	1.143282
4	N	-1.296630	2.979969	-0.565551
5	O	-5.022751	-1.543542	2.991395
6	C	1.119787	-1.925534	-0.711523
7	C	1.744807	-2.835436	-1.687817
8	C	-2.777605	1.051359	-0.853442
9	C	-1.464437	-0.833359	0.958145
10	C	-1.484821	1.679250	-0.528272
11	C	0.694248	1.978566	-0.156237
12	C	-0.208317	-0.414300	0.190525
13	C	0.049353	3.191202	-0.350585
14	C	2.758121	-2.123322	1.405074
15	C	1.627483	-1.543924	0.661459
16	C	-1.794412	-0.143311	2.135101
17	H	-1.127167	0.629022	2.504027
18	C	0.594819	4.559892	-0.383522
19	C	2.133723	1.704135	-0.004337
20	C	3.141769	-2.895841	-1.820285
21	H	3.769962	-2.295257	-1.171193
22	C	0.937966	-3.596233	-2.548457
23	H	-0.141124	-3.532160	-2.450335
24	C	-2.305954	-1.847159	0.510284
25	H	-2.062226	-2.382891	-0.399175
26	C	-3.930966	1.457715	-0.168350
27	H	-3.847200	2.224366	0.593857
28	C	-2.977322	-0.416373	2.802501
29	H	-3.259932	0.128110	3.697692
30	C	-5.240803	-0.156236	-1.402123
31	C	1.904786	4.844524	-0.804877
32	H	2.552269	4.039902	-1.134769
33	C	-2.878149	0.078000	-1.859882
34	H	-1.983592	-0.235667	-2.386981
35	C	3.075698	-3.487432	1.308282
36	H	2.494867	-4.133864	0.658738
37	C	3.505047	-1.301700	2.264571
38	H	3.252787	-0.249950	2.332992
39	C	3.717993	-3.709200	-2.794802
40	H	4.799069	-3.743632	-2.896188
41	C	-3.855715	-1.396053	2.308183

Tag	Symbol	X	Y	Z
42	C	-5.153819	0.852834	-0.427796
43	H	-6.038207	1.136495	0.133482
44	C	1.518418	-4.418534	-3.510708
45	H	0.887085	-5.012230	-4.165836
46	C	2.858413	2.268636	1.055730
47	H	2.331896	2.864686	1.794456
48	C	1.547799	7.210045	-0.427968
49	H	1.917177	8.232111	-0.442258
50	C	2.375700	6.156645	-0.821161
51	H	3.391394	6.356173	-1.153315
52	C	-4.099736	-0.522767	-2.135911
53	H	-4.175065	-1.291313	-2.898642
54	C	2.909528	-4.476824	-3.635764
55	H	3.361109	-5.117031	-4.388640
56	C	-3.500362	-2.133272	1.173242
57	H	-4.155880	-2.897780	0.774123
58	N	-7.478815	-1.431739	-1.723587
59	C	-0.235651	5.629514	-0.007088
60	H	-1.256239	5.415753	0.293845
61	C	2.819621	0.947818	-0.967985
62	H	2.266785	0.522454	-1.800261
63	C	-6.479200	-0.848189	-1.598954
64	C	4.563993	-1.832458	2.995071
65	H	5.145703	-1.186018	3.646470
66	C	4.236951	2.077821	1.152089
67	H	4.785612	2.524972	1.976717
68	C	4.128180	-4.017498	2.053475
69	H	4.361194	-5.075917	1.978050
70	C	4.908388	1.308000	0.199516
71	H	5.980464	1.151660	0.280406
72	C	0.237337	6.939715	-0.026617
73	H	-0.419617	7.752667	0.272197
74	C	4.878412	-3.190952	2.892190
75	H	5.704038	-3.603846	3.465610
76	C	4.194093	0.740721	-0.859222
77	H	4.709207	0.148356	-1.610985
78	C	-6.007406	-2.423377	2.459319
79	H	-6.300008	-2.132307	1.441724
80	H	-6.870004	-2.340066	3.122558
81	H	-5.656825	-3.464037	2.452214

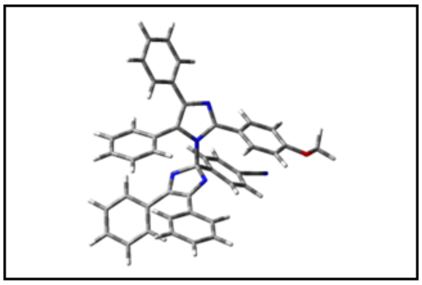

OMe-CN

Tag	Symbol	X	Y	Z
1	N	0.113368	-1.283209	-0.974054
2	N	-0.334846	0.965727	-0.251609
3	N	0.930688	-0.578744	1.149818
4	N	-1.411543	2.884232	-0.632115
5	C	1.215286	-1.894116	-0.705919
6	C	1.882221	-2.775538	-1.679985
7	C	-2.790812	0.859893	-0.888326
8	C	-1.412323	-0.911708	0.967808
9	C	-1.542601	1.578361	-0.577513
10	C	0.616044	1.992334	-0.146832
11	C	-0.175420	-0.436030	0.189838
12	C	-0.083109	3.165369	-0.379978
13	C	2.859610	-2.014136	1.413478
14	C	1.704076	-1.489157	0.668831
15	C	-1.748683	-0.241455	2.151098
16	H	-1.115006	0.565090	2.503574
17	C	0.392592	4.559730	-0.423121
18	C	2.064886	1.790344	0.029887
19	C	3.280955	-2.774705	-1.808089
20	H	3.880535	-2.146759	-1.157780
21	C	1.111391	-3.569287	-2.544025
22	H	0.030117	-3.550660	-2.450891
23	C	-2.197387	-1.975253	0.522712
24	H	-1.938769	-2.485268	-0.396073
25	C	-3.951820	1.132394	-0.159389
26	H	-3.919896	1.888850	0.618191
27	C	-2.904380	-0.579360	2.842302
28	H	-3.186058	-0.041036	3.741371
29	C	-5.156067	-0.575638	-1.369104
30	C	1.699103	4.906464	-0.807319
31	H	2.398633	4.132187	-1.101446
32	C	-2.845503	-0.102869	-1.910279
33	H	-1.950773	-0.322753	-2.483259
34	C	3.237925	-3.363019	1.320363
35	H	2.687879	-4.036474	0.671482
36	C	3.567789	-1.156526	2.270654
37	H	3.269489	-0.116585	2.334548
38	C	3.894838	-3.561755	-2.781161
39	H	4.976611	-3.548760	-2.879690
40	C	-3.729863	-1.608680	2.356473
41	C	-5.128489	0.419491	-0.382900
42	H	-6.000132	0.628676	0.225697
43	C	1.730062	-4.365163	-3.504522
44	H	1.127106	-4.984586	-4.162483
45	C	2.744230	2.396769	1.096920
46	H	2.176889	2.970767	1.822845
47	C	1.206511	7.256194	-0.487939
48	H	1.522118	8.296024	-0.510033
49	C	2.100915	6.241251	-0.833746
50	H	3.115246	6.488374	-1.136990
51	C	-4.015247	-0.809122	-2.152606
52	H	-4.066925	-1.567682	-2.927435
53	C	3.122871	-4.363332	-3.624714
54	H	3.604107	-4.982870	-4.376484
55	C	-3.358746	-2.319738	1.205783
56	H	-4.000437	-3.109156	0.828944
57	C	-0.504913	5.590755	-0.095455
58	H	-1.522766	5.328153	0.174324
59	C	2.804366	1.063981	-0.917571
60	H	2.287137	0.607686	-1.756500
61	C	4.648821	-1.637173	3.003398
62	H	5.201032	-0.963145	3.652405
63	C	4.129495	2.278466	1.214829
64	H	4.641816	2.759435	2.043858
65	C	4.311782	-3.843090	2.068743
66	H	4.592199	-4.890159	1.997071
67	C	4.854252	1.538341	0.278039
68	H	5.931673	1.438279	0.375526
69	C	-0.100729	6.923623	-0.124612
70	H	-0.809307	7.705718	0.136380
71	C	5.023159	-2.980786	2.905453
72	H	5.865782	-3.354517	3.480958
73	C	4.185678	0.927879	-0.786752
74	H	4.742010	0.358471	-1.526920
75	C	-4.982784	-1.897659	2.987991
76	N	-6.020377	-2.123010	3.464638
77	O	-6.231247	-1.370491	-1.633824
78	C	-7.364543	-1.271594	-0.779218
79	H	-8.067591	-2.030215	-1.127934
80	H	-7.098510	-1.473562	0.267325
81	H	-7.837894	-0.283045	-0.847876

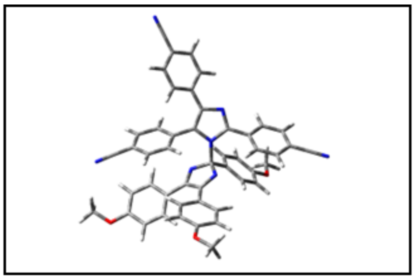

$(CN)_3$-$(OMe)_3$

Tag	Symbol	X	Y	Z
1	N	-0.067827	-1.623594	-0.653322
2	N	-1.316670	0.390350	-0.263459
3	N	0.487027	-0.282957	1.227208
4	N	-3.084320	1.630080	-0.845984
5	C	1.198924	-1.706570	-0.423285
6	C	2.101140	-2.430717	-1.330525
7	C	-3.533973	-0.771144	-0.751062
8	C	-1.567369	-1.490785	1.342598
9	C	-2.663898	0.407914	-0.596338
10	C	-0.873458	1.713632	-0.356150
11	C	-0.618654	-0.757838	0.392649
12	C	-1.991073	2.456182	-0.716632
13	C	2.856273	-0.718238	1.441975
14	C	1.546003	-0.895056	0.811269
15	C	-2.153023	-0.771436	2.386339
16	H	-1.917303	0.281763	2.504727
17	C	-2.126224	3.894572	-0.991564
18	C	0.516208	2.169350	-0.190758
19	C	3.363766	-1.913410	-1.680709
20	H	3.712698	-0.990582	-1.229977
21	C	1.668374	-3.611039	-1.949973
22	H	0.688722	-4.006372	-1.700375
23	C	-1.853338	-2.853480	1.212134
24	H	-1.412713	-3.418793	0.399570
25	C	-4.803978	-0.761646	-0.156556
26	H	-5.101640	0.097184	0.435032
27	C	-3.044559	-1.379848	3.267456
28	H	-3.491743	-0.792671	4.060762
29	C	-5.260919	-2.949878	-1.077123
30	C	-1.069967	4.679363	-1.488549
31	H	-0.110748	4.223177	-1.702668
32	C	-3.146034	-1.867399	-1.536387
33	H	-2.161796	-1.876843	-1.990116
34	C	3.880849	-1.683205	1.381900
35	H	3.697088	-2.642125	0.910409
36	C	3.118004	0.485478	2.114952
37	H	2.324644	1.218942	2.193736
38	C	4.163842	-2.562625	-2.607090
39	H	5.134019	-2.167459	-2.889710
40	C	-3.344755	-2.741309	3.114650
41	C	-5.660905	-1.844333	-0.307145
42	H	-6.635871	-1.846017	0.169196
43	C	2.470314	-4.282268	-2.869250
44	H	2.111178	-5.201393	-3.316648
45	C	0.820725	3.176205	0.739822
46	H	0.026705	3.580889	1.358484
47	C	-2.487771	6.642038	-1.495585
48	C	-1.242608	6.036019	-1.732825
49	H	-0.420957	6.630908	-2.118894
50	C	-4.001180	-2.950040	-1.698920
51	H	-3.701222	-3.800389	-2.302929
52	C	3.727878	-3.757136	-3.204067
53	C	-2.734648	-3.474416	2.086414
54	H	-2.985365	-4.524870	1.981394
55	C	-3.375448	4.507338	-0.780712
56	H	-4.200968	3.899060	-0.427436
57	C	1.544333	1.668122	-1.007442
58	H	1.313057	0.905437	-1.744468
59	C	4.375809	0.771168	2.629090
60	H	4.554443	1.731821	3.096183
61	C	2.122106	3.643326	0.883281
62	H	2.353479	4.405071	1.620865
63	C	5.134740	-1.419933	1.911400
64	H	5.934009	-2.151649	1.856710
65	C	3.152044	3.098679	0.097787
66	C	-3.557882	5.861712	-1.023985
67	H	-4.524033	6.325141	-0.851078
68	C	5.406371	-0.174647	2.503785
69	C	2.851479	2.114779	-0.860180
70	H	3.645809	1.698913	-1.471670
71	O	4.591925	-4.320383	-4.086291
72	O	6.680730	0.020617	2.917345
73	C	7.055878	1.325198	3.362175
74	H	6.541744	1.591419	4.294825
75	H	8.130018	1.274665	3.546514
76	H	6.846348	2.081416	2.595524
77	C	4.210666	-5.526051	-4.739051
78	H	3.304459	-5.386225	-5.342673
79	H	5.044567	-5.785940	-5.392810
80	H	4.046338	-6.337915	-4.018519
81	O	-4.208219	-3.438329	3.902118
82	C	-4.881500	-2.747438	4.944970
83	H	-5.500221	-1.929342	4.551998
84	H	-5.523987	-3.486673	5.426301
85	H	-4.175830	-2.343895	5.683510
86	C	-6.133311	-4.077040	-1.224456
87	N	-6.837496	-4.995861	-1.341807
88	C	-2.666486	8.041889	-1.740140
89	N	-2.810153	9.180087	-1.935680
90	C	4.511607	3.476815	0.340999
91	N	5.617059	3.750892	0.580723

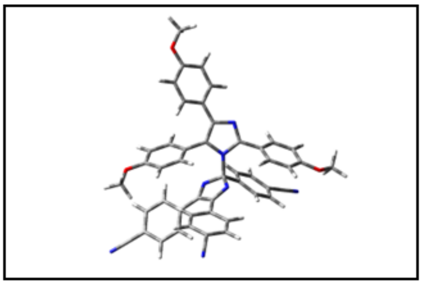

(OMe)$_3$-(CN)$_3$

Tag	Symbol	X	Y	Z
1	N	-0.173222	-1.555928	0.832005
2	N	1.270050	0.251691	0.189917
3	N	-0.595912	-0.437024	-1.227439
4	N	3.124170	1.393381	0.678915
5	C	-1.435810	-1.552643	0.586540
6	C	-2.419663	-2.056561	1.563804
7	C	3.374829	-1.055353	0.756670
8	C	1.317771	-1.840363	-1.151923
9	C	2.620234	0.190692	0.530498
10	C	0.923783	1.616488	0.178634
11	C	0.466137	-0.879588	-0.307465
12	C	2.094549	2.292687	0.476159
13	C	-2.987235	-0.741190	-1.446548
14	C	-1.698833	-0.895893	-0.749253
15	C	1.917626	-1.340059	-2.314688
16	H	1.740821	-0.309862	-2.604196
17	C	2.342837	3.737120	0.617017
18	C	-0.436800	2.140436	-0.023649
19	C	-3.624114	-1.369213	1.786627
20	H	-3.850601	-0.478657	1.210535
21	C	-2.118920	-3.190209	2.334760
22	H	-1.177724	-3.704677	2.172226
23	C	1.509404	-3.172485	-0.785295
24	H	1.054622	-3.552817	0.120007
25	C	4.511558	-1.329209	-0.009234
26	H	4.834473	-0.604906	-0.750206
27	C	2.765492	-2.144089	-3.064729
28	H	3.258048	-1.751626	-3.948276
29	C	4.767748	-3.474462	1.069708
30	C	1.343762	4.657477	0.988124
31	H	0.338920	4.310164	1.199402
32	C	2.978856	-1.985527	1.732380
33	H	2.102095	-1.781720	2.338062
34	C	-4.009987	-1.696659	-1.336590
35	H	-3.855294	-2.596224	-0.751083
36	C	-3.193524	0.400316	-2.238250
37	H	-2.396737	1.128064	-2.325911
38	C	-4.518147	-1.811571	2.754721
39	H	-5.445134	-1.276739	2.933079
40	C	3.008684	-3.467906	-2.658721
41	C	5.204983	-2.530138	0.131061
42	H	6.060296	-2.727167	-0.504147
43	C	-3.012716	-3.646457	3.295340
44	H	-2.785357	-4.528405	3.884921
45	C	-0.713008	3.028908	-1.078733
46	H	0.089403	3.309787	-1.753603
47	C	2.921541	6.490020	0.872400
48	C	1.625252	6.010718	1.109389
49	H	0.856726	6.720206	1.400081
50	C	3.668639	-3.179225	1.891453
51	H	3.364895	-3.913475	2.631096
52	C	-4.220089	-2.958632	3.508808
53	C	2.359782	-3.984603	-1.527651
54	H	2.555442	-5.003761	-1.211696
55	C	3.637286	4.230329	0.401992
56	H	4.424761	3.530218	0.142382
57	C	-1.476482	1.823605	0.858314
58	H	-1.274947	1.169671	1.702144
59	C	-4.414607	0.615940	-2.861039
60	H	-4.582285	1.517931	-3.439967
61	C	-1.987903	3.544840	-1.266447
62	H	-2.208791	4.220064	-2.087117
63	C	-5.230089	-1.499735	-1.974673
64	H	-6.019964	-2.238613	-1.889562
65	C	-3.035878	3.163455	-0.414245
66	C	3.933561	5.588097	0.522479
67	H	4.947897	5.926115	0.343530
68	C	-5.444712	-0.332566	-2.726072
69	C	-2.771114	2.311074	0.666329
70	H	-3.553487	2.032431	1.363122
71	C	3.965444	-4.278569	-3.351475
72	N	4.764895	-4.940410	-3.877811
73	O	5.332540	-4.700646	1.251893
74	C	6.371373	-5.100232	0.364797
75	H	6.631466	-6.120605	0.651669
76	H	6.034378	-5.089353	-0.680703
77	H	7.257619	-4.459955	0.466290
78	C	-5.147385	-3.425926	4.497394
79	N	-5.901600	-3.805048	5.297346
80	C	-6.715623	-0.105389	-3.349116
81	N	-7.749570	0.084439	-3.846911
82	O	-4.266155	3.651357	-0.736666
83	O	3.090392	7.838343	1.015463
84	C	4.386357	8.375989	0.806913
85	H	4.739271	8.197817	-0.218392
86	H	4.296158	9.451150	0.974341
87	H	5.116794	7.959334	1.514124
88	C	-5.388600	3.188899	0.002249
89	H	-5.331930	3.489266	1.056900
90	H	-6.261875	3.654364	-0.457527
91	H	-5.483350	2.096006	-0.061798

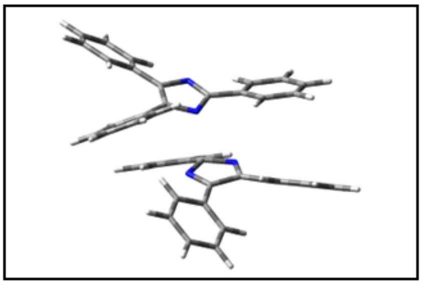

H-H_TS

Tag	Symbol	X	Y	Z
1	N	0.113368	-1.283209	-0.974054
2	N	-0.334846	0.965727	-0.251609
3	N	0.930688	-0.578744	1.149818
4	N	-1.411543	2.884232	-0.632115
5	C	1.215286	-1.894116	-0.705919
6	C	1.882221	-2.775538	-1.679985
7	C	-2.790812	0.859893	-0.888326
8	C	-1.412323	-0.911708	0.967808
9	C	-1.542601	1.578361	-0.577513
10	C	0.616044	1.992334	-0.146832
11	C	-0.175420	-0.436030	0.189838
12	C	-0.083109	3.165369	-0.379978
13	C	2.859610	-2.014136	1.413478
14	C	1.704076	-1.489157	0.668831
15	C	-1.748683	-0.241455	2.151098
16	H	-1.115006	0.565090	2.503574
17	C	0.392592	4.559730	-0.423121
18	C	2.064886	1.790344	0.029887
19	C	3.280955	-2.774705	-1.808089
20	H	3.880535	-2.146759	-1.157780
21	C	1.111391	-3.569287	-2.544025
22	H	0.030117	-3.550660	-2.450891
23	C	-2.197387	-1.975253	0.522712
24	H	-1.938769	-2.485268	-0.396073
25	C	-3.951820	1.132394	-0.159389
26	H	-3.919896	1.888850	0.618191
27	C	-2.904380	-0.579360	2.842302
28	H	-3.186058	-0.041036	3.741371
29	C	-5.156067	-0.575638	-1.369104
30	C	1.699103	4.906464	-0.807319
31	H	2.398633	4.132187	-1.101446
32	C	-2.845503	-0.102869	-1.910279
33	H	-1.950773	-0.322753	-2.483259
34	C	3.237925	-3.363019	1.320363
35	H	2.687879	-4.036474	0.671482
36	C	3.567789	-1.156526	2.270654
37	H	3.269489	-0.116585	2.334548
38	C	3.894838	-3.561755	-2.781161
39	H	4.976611	-3.548760	-2.879690
40	C	-3.729863	-1.608680	2.356473
41	C	-5.128489	0.419491	-0.382900
42	H	-6.000132	0.628676	0.225697
43	C	1.730062	-4.365163	-3.504522
44	H	1.127106	-4.984586	-4.162483
45	C	2.744230	2.396769	1.096920
46	H	2.176889	2.970767	1.822845
47	C	1.206511	7.256194	-0.487939
48	H	1.522118	8.296024	-0.510033
49	C	2.100915	6.241251	-0.833746
50	H	3.115246	6.488374	-1.136990
51	C	-4.015247	-0.809122	-2.152606
52	H	-4.066925	-1.567682	-2.927435
53	C	3.122871	-4.363332	-3.624714
54	H	3.604107	-4.982870	-4.376484
55	C	-3.358746	-2.319738	1.205783
56	H	-4.000437	-3.109156	0.828944
57	C	-0.504913	5.590755	-0.095455
58	H	-1.522766	5.328153	0.174324
59	C	2.804366	1.063981	-0.917571
60	H	2.287137	0.607686	-1.756500
61	C	4.648821	-1.637173	3.003398
62	H	5.201032	-0.963145	3.652405
63	C	4.129495	2.278466	1.214829
64	H	4.641816	2.759435	2.043858
65	C	4.311782	-3.843090	2.068743
66	H	4.592199	-4.890159	1.997071
67	C	4.854252	1.538341	0.278039
68	H	5.931673	1.438279	0.375526
69	C	-0.100729	6.923623	-0.124612
70	H	-0.809307	7.705718	0.136380
71	C	5.023159	-2.980786	2.905453
72	H	5.865782	-3.354517	3.480958
73	C	4.185678	0.927879	-0.786752
74	H	4.742010	0.358471	-1.526920
75	C	-4.982784	-1.897659	2.987991
76	N	-6.020377	-2.123010	3.464638
77	O	-6.231247	-1.370491	-1.633824
78	C	-7.364543	-1.271594	-0.779218
79	H	-8.067591	-2.030215	-1.127934
80	H	-7.098510	-1.473562	0.267325
81	H	-7.837894	-0.283045	-0.847876

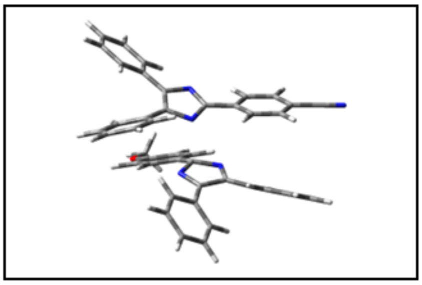

CN-OMe_TS

Tag	Symbol	X	Y	Z	Tag	Symbol	X	Y	Z
1	N	1.487985	-0.522181	0.857254	42	C	0.953791	-4.806721	-0.987124
2	N	-0.432132	-0.062194	-1.095057	43	H	0.711663	-5.829778	-0.716643
3	N	0.667214	1.663805	0.735858	44	C	5.494352	-1.969765	0.883265
4	N	-1.996992	-1.724023	-1.134684	45	H	5.771805	-2.898478	1.373582
5	O	-3.876903	-0.972948	4.353158	46	C	-2.806277	2.722396	-0.676274
6	C	2.491606	0.285104	0.658313	47	H	-3.508490	2.203640	-0.030992
7	C	3.857136	-0.222175	0.470517	48	C	-6.882360	-0.576319	-1.805770
8	C	0.315747	-2.471548	-1.199336	49	H	-7.958888	-0.610784	-1.951100
9	C	-0.756043	-0.064476	1.755938	50	C	-6.141995	0.508245	-2.281633
10	C	-0.711810	-1.436832	-1.094192	51	H	-6.639675	1.317589	-2.809708
11	C	-1.669607	0.526615	-1.185819	52	C	2.550858	-3.179534	-1.832787
12	C	0.351739	0.299378	0.894706	53	H	3.539210	-2.947261	-2.216003
13	C	-2.637261	-0.494210	-1.212377	54	C	6.402179	-1.325705	0.036608
14	C	2.691122	2.975469	0.549978	55	H	7.387229	-1.752599	-0.130353
15	C	1.963877	1.701677	0.619220	56	C	-1.908139	-1.722880	3.116858
16	C	-1.705649	0.902984	2.157242	57	H	-1.979713	-2.741026	3.480620
17	H	-1.605402	1.917085	1.789843	58	N	4.038719	-6.364540	-1.670346
18	C	-4.089056	-0.481219	-1.425426	59	C	-4.845142	-1.583141	-0.976859
19	C	-1.798853	1.988828	-1.327094	60	H	-4.325581	-2.403235	-0.493372
20	C	4.776212	0.415887	-0.382301	61	C	-0.898615	2.685547	-2.152473
21	H	4.495678	1.330005	-0.893967	62	H	-0.118910	2.126795	-2.660480
22	C	4.229866	-1.429378	1.091824	63	C	3.230170	-5.531135	-1.583586
23	H	3.511609	-1.929323	1.732658	64	C	2.697137	5.328531	-0.048479
24	C	-0.882236	-1.379126	2.248575	65	H	2.200103	6.182679	-0.500093
25	H	-0.156453	-2.124721	1.944143	66	C	-2.913233	4.101723	-0.849706
26	C	0.004897	-3.802278	-0.861502	67	H	-3.698723	4.648376	-0.334023
27	H	-0.993803	-4.027271	-0.503074	68	C	4.604203	4.372939	1.090284
28	C	-2.729219	0.567997	3.023837	69	H	5.588521	4.482190	1.536920
29	H	-3.460462	1.302074	3.346246	70	C	-2.016598	4.780258	-1.678121
30	C	2.240985	-4.502481	-1.467637	71	H	-2.105197	5.854410	-1.817993
31	C	-4.762268	0.557703	-2.095912	72	C	-6.224617	-1.623431	-1.154986
32	H	-4.200820	1.396215	-2.490960	73	H	-6.789031	-2.478958	-0.792476
33	C	1.596269	-2.181567	-1.702310	74	C	3.973674	5.471840	0.503716
34	H	1.826731	-1.163966	-1.995131	75	H	4.471649	6.437486	0.482281
35	C	3.971802	3.131252	1.111501	76	C	-1.009102	4.064342	-2.329642
36	H	4.462800	2.288086	1.584790	77	H	-0.311570	4.579736	-2.985708
37	C	2.054053	4.095354	-0.017951	78	C	-4.066311	-2.283639	4.875403
38	H	1.058288	3.978022	-0.431548	79	H	-4.241800	-3.010806	4.073337
39	C	6.037028	-0.137877	-0.599615	80	H	-4.950848	-2.223120	5.510908
40	H	6.732590	0.357951	-1.270866	81	H	-3.206031	-2.602226	5.476837
41	C	-2.841779	-0.747176	3.510444					

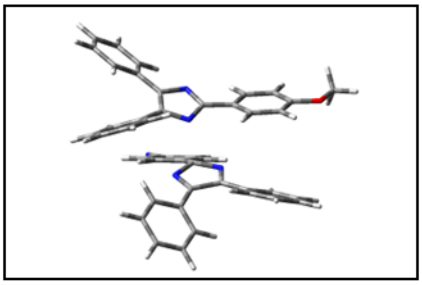

OMe-CN_TS

Tag	Symbol	X	Y	Z	Tag	Symbol	X	Y	Z
1	C	-3.926471	0.237980	0.204747	42	C	-1.885601	3.648708	-1.694407
2	C	0.176341	2.536270	-1.050954	43	H	-2.896442	3.623105	-2.088623
3	C	-2.034812	-1.629790	0.683689	44	C	6.935098	-0.586444	-1.519062
4	C	1.004484	1.344482	-0.954742	45	C	1.583515	2.000790	3.198589
5	C	1.598213	-0.765400	-1.062934	46	H	1.569291	3.005181	3.609120
6	C	-0.455972	-0.178280	0.901837	47	C	6.455453	0.552082	-0.865589
7	C	0.604057	0.274763	1.803780	48	H	7.151453	1.286517	-0.468917
8	C	-2.743560	-2.915486	0.720602	49	C	-5.590477	2.007522	0.290066
9	C	-2.576919	-0.229943	0.546084	50	H	-5.900827	2.986703	0.643960
10	C	0.587638	1.582532	2.332063	51	C	-4.789612	-0.510576	-0.616357
11	H	-0.216010	2.252498	2.050981	52	H	-4.475645	-1.478708	-0.990461
12	C	4.161933	-0.189992	-1.216106	53	C	-1.338828	4.891727	-1.330158
13	C	-4.053743	-3.025789	1.222193	54	C	-6.036213	-0.002430	-0.977748
14	H	-4.579384	-2.141081	1.564462	55	H	-6.687660	-0.586730	-1.621577
15	C	2.732524	0.076720	-1.034101	56	C	-6.443670	1.251958	-0.520334
16	C	1.641366	-0.604105	2.170043	57	C	-0.027582	4.957263	-0.835650
17	H	1.626165	-1.620204	1.796366	58	H	0.417167	5.905021	-0.554688
18	C	5.086445	0.756650	-0.727930	59	C	2.204067	-4.507256	-0.807069
19	H	4.705872	1.648253	-0.242230	60	H	2.848590	-5.195100	-0.265824
20	C	1.488584	-2.223156	-1.257795	61	C	0.447455	-4.099618	-2.410365
21	C	-2.064169	-4.084570	0.328711	62	H	-0.277220	-4.468245	-3.132070
22	H	-1.047727	-4.002489	-0.037953	63	C	1.275468	-4.995963	-1.729215
23	C	0.716345	3.788100	-0.707791	64	N	2.319786	1.395836	-0.943114
24	C	-4.339815	1.511274	0.642373	65	N	0.484078	0.023547	-0.999638
25	H	-3.664209	2.095012	1.258056	66	N	-0.744523	-1.556575	0.863509
26	C	-2.692655	-5.323130	0.408104	67	N	-1.595256	0.611098	0.730436
27	H	-2.160597	-6.215654	0.090487	68	H	8.004553	-0.742118	-1.633646
28	C	0.549252	-2.729480	-2.173552	69	H	1.735499	3.829246	-0.338310
29	H	-0.090753	-2.031098	-2.703697	70	H	-1.558203	1.537312	-1.849946
30	C	-3.998612	-5.421526	0.897512	71	H	6.392861	-2.392994	-2.566378
31	C	-4.672789	-4.271141	1.311554	72	H	1.199815	-6.063859	-1.916722
32	H	-5.680759	-4.342559	1.710728	73	H	-7.419148	1.642339	-0.797414
33	C	6.029921	-1.516170	-2.036528	74	C	3.654043	1.553683	4.451870
34	C	-1.139661	2.491830	-1.554921	75	N	4.488336	1.909327	5.181365
35	C	2.309935	-3.136600	-0.574576	76	H	-4.485429	-6.390957	0.962320
36	H	3.040228	-2.766318	0.138671	77	O	-2.156097	5.967282	-1.499512
37	C	2.645227	-0.184409	3.035981	78	C	-1.652738	7.256982	-1.182410
38	H	3.443548	-0.862110	3.320418	79	H	-0.777262	7.508651	-1.794589
39	C	2.624811	1.119701	3.554712	80	H	-2.461628	7.955507	-1.403604
40	C	4.658651	-1.324225	-1.887287	81	H	-1.385576	7.335402	-0.120549
41	H	3.969707	-2.044632	-2.311739					

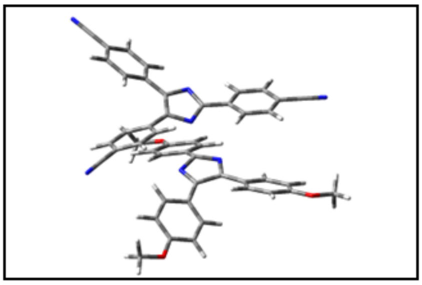

(CN)$_3$-(OMe)$_3$_TS

Tag	Symbol	X	Y	Z
1	N	1.458238	-1.036014	0.769468
2	N	-0.662283	-0.599015	-0.926313
3	N	0.565195	1.122401	0.863109
4	N	-2.403554	-2.063296	-0.960458
5	C	2.420815	-0.187822	0.544516
6	C	3.763070	-0.640097	0.186483
7	C	-0.183494	-3.069411	-1.018990
8	C	-0.701214	-0.699640	1.907251
9	C	-1.088356	-1.921752	-0.929001
10	C	-1.831842	0.129188	-0.992806
11	C	0.307611	-0.256729	0.961783
12	C	-2.904203	-0.776697	-1.011771
13	C	2.530087	2.510241	0.662229
14	C	1.851049	1.219251	0.662875
15	C	-1.676029	0.197060	2.384281
16	H	-1.666295	1.218966	2.024046
17	C	-4.351844	-0.595797	-1.171913
18	C	-1.791183	1.590495	-1.138032
19	C	4.614997	0.097044	-0.664806
20	H	4.299069	1.062066	-1.044843
21	C	4.190356	-1.913021	0.609651
22	H	3.534515	-2.496576	1.246470
23	C	-0.698447	-2.025400	2.401245
24	H	0.050247	-2.720095	2.038859
25	C	-0.632496	-4.347824	-0.638879
26	H	-1.643344	-4.452802	-0.260040
27	C	-2.619967	-0.202304	3.322741
28	H	-3.355819	0.510194	3.676214
29	C	1.505090	-5.305331	-1.248239
30	C	-4.928096	0.517639	-1.813132
31	H	-4.292334	1.286905	-2.235255
32	C	1.114277	-2.934871	-1.541932
33	H	1.452525	-1.958006	-1.865424
34	C	3.880129	2.669869	1.046726
35	H	4.469486	1.807060	1.334989
36	C	1.786939	3.669382	0.365404
37	H	0.742154	3.561614	0.101903
38	C	5.839710	-0.413453	-1.061083
39	H	6.490093	0.141037	-1.729622
40	C	-2.603794	-1.521919	3.803182
41	C	0.199673	-5.454101	-0.745387
42	H	-0.148893	-6.436070	-0.441298
43	C	5.424328	-2.427526	0.232501
44	H	5.724616	-3.405418	0.589931
45	C	-2.675210	2.446994	-0.454984
46	H	-3.418886	2.022504	0.211777
47	C	-7.153217	-0.361449	-1.431466
48	C	-6.306038	0.638118	-1.939926
49	H	-6.735096	1.499480	-2.442149
50	C	1.951719	-4.035011	-1.655497
51	H	2.953201	-3.922601	-2.058112
52	C	6.260720	-1.677249	-0.611530
53	C	-1.630657	-2.428368	3.335462
54	H	-1.639229	-3.440584	3.726250
55	C	-5.212138	-1.606186	-0.694825
56	H	-4.772506	-2.484262	-0.235310
57	C	-0.836180	2.168710	-1.996711
58	H	-0.155803	1.515721	-2.532688
59	C	2.363033	4.931808	0.396087
60	H	1.761093	5.796004	0.139679
61	C	-2.600266	3.826236	-0.606042
62	H	-3.276272	4.475562	-0.058551
63	C	4.460750	3.925537	1.098478
64	H	5.493563	4.054973	1.405067
65	C	-1.634708	4.390163	-1.459067
66	C	-6.589477	-1.491671	-0.812327
67	H	-7.239543	-2.273366	-0.431752
68	C	3.713245	5.068389	0.761649
69	C	-0.758876	3.544364	-2.164248
70	H	-0.019532	3.975828	-2.831919
71	O	7.476029	-2.081853	-1.048199
72	O	4.376499	6.245409	0.827594
73	C	3.676099	7.446870	0.511862
74	H	2.834654	7.608692	1.195924
75	H	4.403010	8.251036	0.631871
76	H	3.310037	7.435208	-0.521578
77	C	7.950711	-3.369626	-0.663644
78	H	7.280911	-4.163014	-1.016179
79	H	8.925732	-3.478228	-1.140104
80	H	8.066090	-3.443215	0.424610
81	O	-3.470957	-2.018509	4.714206
82	C	-4.497686	-1.171438	5.222206
83	H	-5.156349	-0.819586	4.419165
84	H	-5.069168	-1.786101	5.918712
85	H	-4.076175	-0.310686	5.755462
86	C	2.381343	-6.434143	-1.341934
87	N	3.106671	-7.342373	-1.410460
88	C	-8.574597	-0.233933	-1.548964
89	N	-9.730527	-0.128493	-1.638205
90	C	-1.511904	5.811489	-1.582886
91	N	-1.382500	6.965494	-1.669205

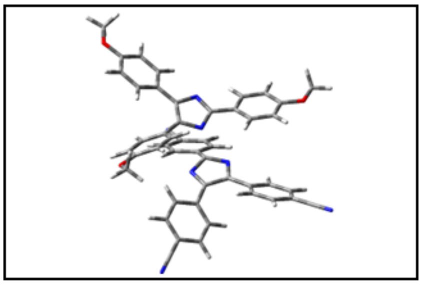

(OMe)$_3$-(CN)$_3$_TS

Tag	Symbol	X	Y	Z
1	N	1.514868	-1.262237	0.722605
2	N	-0.578228	-0.404843	-0.770161
3	N	0.839511	0.939777	1.072669
4	N	-2.563787	-1.548633	-0.751339
5	C	2.545006	-0.478140	0.552974
6	C	3.837547	-1.020898	0.106065
7	C	-0.584408	-2.937028	-0.970859
8	C	-0.578556	-0.852896	1.973825
9	C	-1.258759	-1.660261	-0.787695
10	C	-1.589542	0.518442	-0.762643
11	C	0.438354	-0.411665	1.006972
12	C	-2.817373	-0.179831	-0.760258
13	C	2.912937	2.168254	0.921798
14	C	2.120672	0.932528	0.825033
15	C	-1.517445	0.067719	2.475256
16	H	-1.455534	1.103965	2.168045
17	C	-4.199321	0.256565	-0.912382
18	C	-1.297400	1.957112	-0.907894
19	C	4.711239	-0.287371	-0.716708
20	H	4.453815	0.721994	-1.017632
21	C	4.175652	-2.345400	0.444131
22	H	3.490779	-2.918210	1.059147
23	C	-0.616671	-2.188952	2.420840
24	H	0.114031	-2.892905	2.041409
25	C	-1.254136	-4.129200	-0.645744
26	H	-2.248294	-4.065860	-0.216167
27	C	-2.484388	-0.339385	3.387749
28	H	-3.207299	0.371465	3.774559
29	C	0.613041	-5.441866	-1.433568
30	C	-4.581172	1.550431	-1.336656
31	H	-3.825454	2.280903	-1.595785
32	C	0.697404	-3.027059	-1.550519
33	H	1.212734	-2.117379	-1.833707
34	C	4.262858	2.167392	1.317265
35	H	4.764743	1.232694	1.540556
36	C	2.273292	3.401398	0.692775
37	H	1.226628	3.404569	0.414527
38	C	5.895646	-0.851814	-1.176575
39	H	6.560251	-0.283542	-1.818986
40	C	-2.523340	-1.673889	3.821220
41	C	-0.666994	-5.372045	-0.863141
42	H	-1.207658	-6.271515	-0.592158
43	C	5.360766	-2.912247	-0.002298
44	H	5.620151	-3.930018	0.270215
45	C	-1.891961	2.943759	-0.098375
46	H	-2.584926	2.647106	0.683564
47	C	-6.925053	0.972029	-1.149461
48	C	-5.914396	1.900507	-1.454739
49	H	-6.206476	2.890783	-1.789704
50	C	1.286555	-4.257393	-1.781496
51	H	2.267660	-4.335982	-2.238914
52	C	6.232919	-2.166627	-0.816106
53	C	-1.576002	-2.596024	3.334096
54	H	-1.603334	-3.624153	3.680209
55	C	-5.228554	-0.670540	-0.647211
56	H	-4.951227	-1.677127	-0.356542
57	C	-0.404925	2.384869	-1.901420
58	H	0.067304	1.640805	-2.535579
59	C	2.967848	4.595336	0.821128
60	H	2.467994	5.540689	0.635723
61	C	-1.615477	4.292063	-0.281685
62	H	-2.073706	5.050474	0.345508
63	C	4.963360	3.360474	1.455383
64	H	6.002413	3.352761	1.767904
65	C	-0.729466	4.701778	-1.290975
66	C	-6.570958	-0.323797	-0.745629
67	H	-7.328761	-1.064231	-0.516680
68	C	4.322900	4.583889	1.198932
69	C	-0.121179	3.736854	-2.103890
70	H	0.565404	4.023027	-2.892623
71	O	1.282041	-6.596592	-1.693195
72	O	-8.199367	1.420533	-1.287071
73	O	-0.524978	6.046989	-1.392131
74	C	0.646005	-7.832467	-1.396596
75	H	1.352458	-8.609306	-1.693425
76	H	0.431156	-7.926915	-0.324405
77	H	-0.285406	-7.950759	-1.964626
78	C	-9.269064	0.529314	-0.998114
79	H	-9.239109	0.192542	0.045754
80	H	-10.184125	1.098395	-1.169280
81	H	-9.253548	-0.343959	-1.662351
82	C	0.327772	6.523242	-2.423320
83	H	-0.046628	6.243680	-3.416595
84	H	0.330026	7.610561	-2.330530
85	H	1.353020	6.147788	-2.306009
86	C	-3.518001	-2.096793	4.761690
87	N	-4.326901	-2.443310	5.523368
88	C	5.045575	5.815171	1.325397
89	N	5.631487	6.815717	1.422156
90	C	7.458354	-2.747570	-1.280040
91	N	8.453391	-3.218984	-1.655788

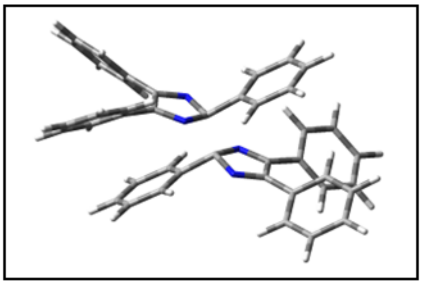

H-H-C2C2

Tag	Symbol	X	Y	Z
1	H	-2.685691	1.998618	3.609400
2	C	-2.224655	1.072413	3.276998
3	C	-1.041693	-1.297948	2.399946
4	C	-1.222610	1.112236	2.306638
5	C	-2.639287	-0.149777	3.810339
6	C	-2.041112	-1.333705	3.372320
7	H	-2.356762	-2.289038	3.783508
8	H	-0.579809	-2.211375	2.043533
9	C	5.832362	-3.375956	0.549509
10	C	3.553502	-2.845935	-0.061370
11	C	4.837016	-1.300846	1.293247
12	C	5.923710	-2.173611	1.253595
13	C	4.644546	-3.709728	-0.107647
14	H	2.625016	-3.089461	-0.566288
15	H	4.912035	-0.367846	1.841779
16	H	6.841934	-1.913157	1.772790
17	H	4.570028	-4.644363	-0.657018
18	H	6.683275	-4.051041	0.512817
19	C	3.561116	1.698087	0.563997
20	C	5.641451	3.546066	0.247835
21	C	4.597219	1.447233	-0.349230
22	C	3.568538	2.888698	1.305670
23	C	4.608462	3.803305	1.153580
24	C	5.628951	2.371381	-0.507603
25	H	4.578615	0.541705	-0.946538
26	H	2.751538	3.082281	1.993750
27	H	4.612028	4.719036	1.738581
28	H	6.422460	2.174022	-1.223192
29	H	6.451093	4.261184	0.128600
30	C	-0.384929	-0.036763	-0.728781
31	N	-1.259273	-1.201858	-0.731633
32	C	-2.464098	-0.744488	-0.683434
33	C	-2.430044	0.760481	-0.689676
34	N	-1.208570	1.165571	-0.765625
35	C	0.630097	-0.074161	-1.856922
36	C	2.639326	-0.149448	-3.810301
37	C	1.041704	-1.297739	-2.400037
38	C	1.222649	1.112434	-2.306490
39	C	2.224704	1.072693	-3.276843
40	C	2.041132	-1.333412	-3.372404
41	H	0.908015	2.054812	-1.873749
42	H	2.685755	1.998926	-3.609149
43	H	2.356775	-2.288709	-3.783683
44	C	-3.641728	-1.629596	-0.635273
45	C	-5.832378	-3.375865	-0.549689
46	C	-4.837033	-1.300690	-1.293248
47	C	-3.553483	-2.845945	0.061146
48	C	-4.644538	-3.709726	0.107380
49	C	-5.923739	-2.173442	-1.253640
50	H	-2.624978	-3.089538	0.565998
51	H	-6.841984	-1.912916	-1.772763
52	H	-6.683301	-4.050939	-0.513029
53	C	-3.561122	1.698118	-0.563923
54	C	-5.641509	3.546028	-0.247715
55	C	-4.597290	1.447145	0.349197
56	C	-3.568511	2.888805	-1.305473
57	C	-4.608460	3.803381	-1.153358
58	C	-5.629048	2.371260	0.507595
59	H	-4.578717	0.541549	0.946402
60	H	-2.751465	3.082474	-1.993474
61	H	-4.611998	4.719174	-1.738261
62	H	-6.422607	2.173809	1.223102
63	H	-6.451171	4.261121	-0.128461
64	C	-0.630078	-0.074322	1.856946
65	C	3.641737	-1.629658	0.635177
66	C	0.384941	-0.036826	0.728801
67	N	1.208582	1.165505	0.765745
68	C	2.430055	0.760427	0.689728
69	C	2.464112	-0.744545	0.683383
70	N	1.259287	-1.201918	0.731547
71	H	-4.570011	-4.644419	0.656649
72	H	-4.912063	-0.367626	-1.841669
73	H	-0.907967	2.054653	1.873988
74	H	0.579805	-2.211195	-2.043718
75	H	-3.422726	-0.179796	4.563302
76	H	3.422772	-0.179403	-4.563259
77	O	-6.231247	-1.370491	-1.633824
78	C	-7.364543	-1.271594	-0.779218
79	H	-8.067591	-2.030215	-1.127934
80	H	-7.098510	-1.473562	0.267325
81	H	-7.837894	-0.283045	-0.847876

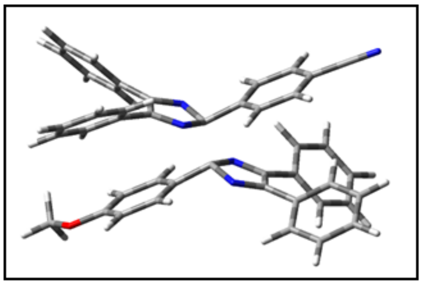

CN-OMe-C2C2

Tag	Symbol	X	Y	Z
1	H	-3.238419	0.274560	-3.649435
2	C	-2.737572	0.814258	-2.852279
3	C	-1.509178	2.223997	-0.790375
4	C	-1.554836	0.344693	-2.298292
5	C	-3.324921	1.993080	-2.368856
6	C	-2.698972	2.706509	-1.338961
7	H	-3.126610	3.623468	-0.950829
8	H	-1.028749	2.763385	0.017726
9	C	5.545505	3.723387	1.031572
10	C	3.431371	2.621311	1.432145
11	C	4.511436	2.424677	-0.727387
12	C	5.554763	3.231363	-0.275020
13	C	4.480448	3.417161	1.883733
14	H	2.595777	2.378691	2.079917
15	H	4.523338	2.044514	-1.743273
16	H	6.375968	3.473449	-0.943821
17	H	4.469803	3.798637	2.901099
18	H	6.364922	4.343175	1.385457
19	C	3.495363	-0.518064	-1.925246
20	C	5.651041	-1.887229	-3.074285
21	C	4.667026	-0.743132	-1.184825
22	C	3.408690	-0.996195	-3.241214
23	C	4.485632	-1.668689	-3.814659
24	C	5.736364	-1.430384	-1.757205
25	H	4.729910	-0.398782	-0.157923
26	H	2.490753	-0.835936	-3.798101
27	H	4.414849	-2.027596	-4.837825
28	H	6.633586	-1.611029	-1.171944
29	H	6.488395	-2.416037	-3.521612
30	C	-0.187728	-0.588209	0.639341
31	N	-1.038030	0.178028	1.535880
32	C	-2.236793	-0.262011	1.349315
33	C	-2.206281	-1.392594	0.354631
34	N	-0.988677	-1.616181	-0.007998
35	C	1.037160	-1.148436	1.331026
36	C	3.446016	-2.038364	2.448315
37	C	1.582781	-0.494634	2.443426
38	C	1.691626	-2.266753	0.797613
39	C	2.889237	-2.710619	1.347193
40	C	2.779300	-0.931836	3.001342
41	H	1.269396	-2.758897	-0.069967
42	H	3.406750	-3.562580	0.918662
43	H	3.209530	-0.415421	3.853373
44	C	-3.404183	0.307513	2.045039
45	C	-5.580188	1.442120	3.399813
46	C	-4.485579	-0.492446	2.446036
47	C	-3.421407	1.681308	2.337322
48	C	-4.505403	2.244403	3.005307
49	C	-5.564494	0.073410	3.124207
50	H	-2.579078	2.289496	2.026301
51	H	-6.392952	-0.556123	3.436759
52	H	-6.425750	1.882419	3.921542
53	C	-3.345679	-2.129442	-0.220805
54	C	-5.453419	-3.513601	-1.437070
55	C	-4.506155	-1.445328	-0.615857
56	C	-3.244325	-3.508918	-0.453336
57	C	-4.297807	-4.198128	-1.050184
58	C	-5.551480	-2.136386	-1.226290
59	H	-4.574792	-0.373030	-0.466536
60	H	-2.334171	-4.024692	-0.163404
61	H	-4.216337	-5.268520	-1.218301
62	H	-6.440727	-1.597220	-1.540719
63	H	-6.272828	-4.051851	-1.905947
64	C	-0.929200	1.039891	-1.251260
65	C	3.440485	2.110220	0.123740
66	C	0.285487	0.457860	-0.562426
67	N	1.128323	-0.315945	-1.464209
68	C	2.328184	0.127601	-1.297121
69	C	2.309170	1.282577	-0.330806
70	N	1.101660	1.478356	0.080248
71	H	-4.513096	3.309659	3.220048
72	H	-4.477881	-1.556402	2.234808
73	H	-1.117521	-0.581848	-2.651201
74	H	1.067893	0.368258	2.848333
75	C	4.699142	-2.470337	2.993660
76	N	5.721740	-2.816186	3.428122
77	O	-4.501311	2.353417	-2.960832
78	C	-5.162855	3.514549	-2.483147
79	H	-6.077604	3.602608	-3.072557
80	H	-5.423050	3.422061	-1.419546
81	H	-4.552583	4.416810	-2.625945

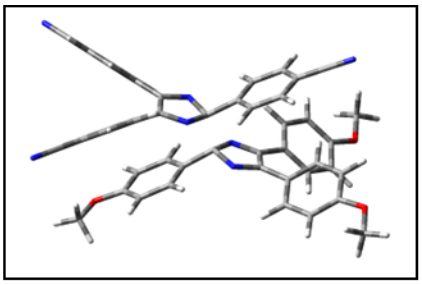

$(CN)_3$-$(OMe)_3$-C2C2

Tag	Symbol	X	Y	Z
1	H	-3.030073	-2.558710	-2.911854
2	C	-2.629227	-1.572658	-2.709746
3	C	-1.561867	0.945897	-2.144399
4	C	-1.477874	-1.452585	-1.932250
5	C	-3.265602	-0.419679	-3.188326
6	C	-2.712947	0.840960	-2.914840
7	H	-3.211374	1.721710	-3.307169
8	H	-1.137688	1.918654	-1.923610
9	C	5.484586	3.447066	-1.518143
10	C	3.290525	2.929130	-0.652143
11	C	4.547283	1.238758	-1.830430
12	C	5.581542	2.140839	-2.023806
13	C	4.327644	3.840718	-0.828549
14	H	2.389145	3.228662	-0.127682
15	H	4.637146	0.234700	-2.230134
16	H	6.480213	1.861010	-2.563625
17	H	4.228570	4.843268	-0.429338
18	C	3.464388	-1.677024	-0.632406
19	C	5.620444	-3.431182	-0.211929
20	C	4.584689	-1.271143	0.118521
21	C	3.440398	-2.977743	-1.150218
22	C	4.509188	-3.850865	-0.958414
23	C	5.645583	-2.136894	0.332360
24	H	4.609298	-0.279511	0.557198
25	H	2.568692	-3.303500	-1.709231
26	H	4.465788	-4.845982	-1.385186
27	H	6.504820	-1.838834	0.924124
28	C	-0.357704	0.044781	0.914419
29	N	-1.144284	1.263691	0.953757
30	C	-2.377442	0.887325	1.004254
31	C	-2.438318	-0.611608	1.063864
32	N	-1.240977	-1.092562	1.095185
33	C	0.812527	0.065663	1.870187
34	C	3.151131	0.084458	3.409091
35	C	1.430622	1.278843	2.198386
36	C	1.356485	-1.136940	2.341002
37	C	2.518201	-1.132605	3.103798
38	C	2.593937	1.293473	2.960991
39	H	0.883522	-2.071884	2.066815
40	H	2.953856	-2.065602	3.445458
41	H	3.082729	2.232658	3.198784
42	C	-3.491778	1.852115	1.018065
43	C	-5.561788	3.740947	1.038700
44	C	-4.671471	1.603904	1.737421
45	C	-3.355728	3.062393	0.317838
46	C	-4.380246	3.999156	0.321174
47	C	-5.699641	2.539805	1.753249
48	H	-2.437535	3.249336	-0.227376
49	H	-6.608109	2.348683	2.314810
50	C	-3.627294	-1.478924	0.979236
51	C	-5.807787	-3.209475	0.679691
52	C	-4.670506	-1.156495	0.095561
53	C	-3.679689	-2.680764	1.700967
54	C	-4.763149	-3.539391	1.561301
55	C	-5.753331	-2.014348	-0.057165
56	H	-4.617053	-0.248517	-0.494675
57	H	-2.858123	-2.933435	2.363055
58	H	-4.807956	-4.466206	2.123811
59	H	-6.555307	-1.767791	-0.745527
60	C	-0.937565	-0.199638	-1.627005
61	C	3.382275	1.615829	-1.135527
62	C	0.220854	-0.083591	-0.666103
63	N	1.083677	-1.253116	-0.663842
64	C	2.290506	-0.803310	-0.768148
65	C	2.254847	0.695653	-0.939655
66	N	1.028216	1.102211	-0.894054
67	H	-4.277972	4.929630	-0.227643
68	H	-4.782817	0.679952	2.294272
69	H	-1.006222	-2.339956	-1.526589
70	H	1.002470	2.203291	1.830569
71	C	4.374932	0.089814	4.155115
72	N	5.374959	0.093512	4.749857
73	C	-6.624591	4.702925	1.041184
74	N	-7.487005	5.483485	1.041787
75	C	-6.924133	-4.095104	0.524342
76	N	-7.829542	-4.813536	0.392911
77	O	6.715302	-4.196355	0.043515
78	O	6.558864	4.247004	-1.748673
79	O	-4.422741	-0.415773	-3.910004
80	C	6.749348	-5.523534	-0.463562
81	H	6.702794	-5.537139	-1.560739
82	H	7.702753	-5.942524	-0.137424
83	H	5.928334	-6.130493	-0.059178
84	C	-5.060274	-1.660409	-4.162696
85	H	-5.969614	-1.423556	-4.717903
86	H	-4.428577	-2.323784	-4.768275
87	H	-5.326058	-2.172748	-3.227641
88	C	6.528721	5.582354	-1.261180
89	H	6.437898	5.609071	-0.167241
90	H	7.480280	6.028068	-1.555561
91	H	5.705239	6.155275	-1.707677

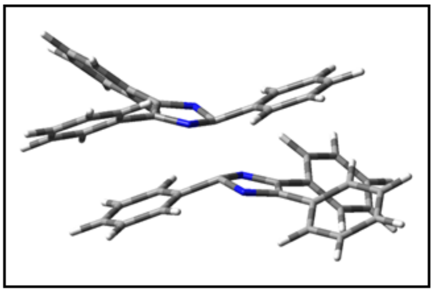

H-H-C2C2_TS

Tag	Symbol	X	Y	Z	Tag	Symbol	X	Y	Z
1	H	3.279799	2.909308	-2.468540	39	C	-2.988385	-2.515642	-1.498945
2	C	2.988385	2.515642	-1.498945	40	C	-2.989668	-2.674345	0.914854
3	C	2.207862	1.522046	0.990327	41	H	-1.869550	-0.864794	-2.327355
4	C	2.203161	1.369699	-1.428416	42	H	-3.279799	-2.909308	-2.468540
5	C	3.383912	3.172458	-0.328660	43	H	-3.286051	-3.183033	1.828073
6	C	2.989668	2.674345	0.914854	44	C	-0.817154	3.408123	1.760309
7	H	3.286051	3.183033	1.828073	45	C	-0.817154	5.407636	3.731772
8	H	1.902167	1.117635	1.947711	46	C	-1.374794	4.669473	1.496373
9	C	0.817154	-5.407636	3.731772	47	C	-0.277435	3.156103	3.033607
10	C	0.277435	-3.156103	3.033607	48	C	-0.271707	4.149946	4.007719
11	C	1.374794	-4.669473	1.496373	49	C	-1.374676	5.660680	2.477349
12	C	1.374676	-5.660680	2.477349	50	H	0.130138	2.172198	3.239488
13	C	0.271707	-4.149946	4.007719	51	H	-1.814049	6.630535	2.260499
14	H	-0.130138	-2.172198	3.239488	52	H	-0.811457	6.183547	4.492687
15	H	1.817578	-4.870685	0.527058	53	C	-0.572038	3.576810	-1.599454
16	H	1.814049	-6.630535	2.260499	54	C	-0.101356	5.736269	-3.326778
17	H	-0.157633	-3.944882	4.984792	55	C	0.281693	4.623001	-1.213480
18	H	0.811457	-6.183547	4.492687	56	C	-1.174007	3.617757	-2.868116
19	C	0.572038	-3.576810	-1.599454	57	C	-0.945337	4.693687	-3.721637
20	C	0.101356	-5.736269	-3.326778	58	C	0.515672	5.693576	-2.075016
21	C	-0.281693	-4.623001	-1.213480	59	H	0.782161	4.579406	-0.253165
22	C	1.174007	-3.617757	-2.868116	60	H	-1.815246	2.795170	-3.168148
23	C	0.945337	-4.693687	-3.721637	61	H	-1.423702	4.719845	-4.697052
24	C	-0.515672	-5.693576	-2.075016	62	H	1.186058	6.492177	-1.768839
25	H	-0.782161	-4.579406	-0.253165	63	H	0.076923	6.575254	-3.994267
26	H	1.815246	-2.795170	-3.168148	64	C	1.809679	0.857377	-0.180195
27	H	1.423702	-4.719845	-4.697052	65	C	0.817154	-3.408123	1.760309
28	H	-1.186058	-6.492177	-1.768839	66	C	0.961219	-0.346373	-0.102320
29	H	-0.076923	-6.575254	-3.994267	67	N	0.919806	-1.205640	-1.224380
30	C	-0.961219	0.346373	-0.102320	68	C	0.783430	-2.405832	-0.734515
31	N	-0.969352	1.078320	1.110852	69	C	0.837494	-2.326349	0.761918
32	C	-0.837494	2.326349	0.761918	70	N	0.969352	-1.078320	1.110852
33	C	-0.783430	2.405832	-0.734515	71	H	0.157633	3.944882	4.984792
34	N	-0.919806	1.205640	-1.224380	72	H	-1.817578	4.870685	0.527058
35	C	-1.809679	-0.857377	-0.180195	73	H	1.869550	0.864794	-2.327355
36	C	-3.383912	-3.172458	-0.328660	74	H	-1.902167	-1.117635	1.947711
37	C	-2.207862	-1.522046	0.990327	75	H	3.990435	4.072476	-0.388418
38	C	-2.203161	-1.369699	-1.428416	76	H	-3.990435	-4.072476	-0.388418

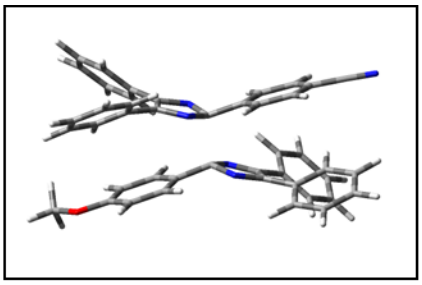

CN-OMe-C2C2_TS

Tag	Symbol	X	Y	Z	Tag	Symbol	X	Y	Z
1	H	3.266139	-2.308627	2.932617	42	H	-3.477660	-2.791968	-2.440036
2	C	2.842236	-1.365367	2.604710	43	H	-3.795178	1.485337	-2.847223
3	C	1.839629	1.083397	1.735840	44	C	3.171270	1.608296	-1.592621
4	C	1.590605	-1.302578	2.020273	45	C	5.143241	3.543488	-2.090863
5	C	3.617012	-0.200345	2.746226	46	C	4.337756	1.271550	-2.297690
6	C	3.106594	1.031356	2.318133	47	C	2.997427	2.933493	-1.157359
7	H	3.681405	1.944565	2.415489	48	C	3.978657	3.890610	-1.399077
8	H	1.436806	2.031293	1.400360	49	C	5.315444	2.234739	-2.545141
9	C	-5.113359	4.046586	1.315798	50	H	2.082638	3.193152	-0.635044
10	C	-3.026486	3.191953	0.443467	51	H	6.210792	1.961473	-3.096744
11	C	-4.351411	1.841523	1.954772	52	H	5.909033	4.291213	-2.279247
12	C	-5.294434	2.865304	2.037269	53	C	3.396851	-1.700386	-0.989894
13	C	-3.974346	4.208061	0.520929	54	C	5.597615	-3.385022	-0.558439
14	H	-2.131427	3.309577	-0.158471	55	C	4.543526	-1.220770	-0.336227
15	H	-4.496009	0.927459	2.520518	56	C	3.362287	-3.038442	-1.415677
16	H	-6.171109	2.738524	2.666292	57	C	4.459142	-3.870657	-1.209517
17	H	-3.826507	5.127823	-0.038542	58	C	5.633314	-2.061323	-0.116717
18	H	-5.854170	4.839428	1.373917	59	H	4.564695	-0.200165	0.027078
19	C	-3.548521	-1.363340	1.218515	60	H	2.464912	-3.409918	-1.900025
20	C	-5.797098	-3.038646	1.152236	61	H	4.425760	-4.901621	-1.551301
21	C	-4.706057	-0.976204	0.523064	62	H	6.504583	-1.683027	0.410547
22	C	-3.529887	-2.604423	1.876599	63	H	6.449913	-4.038456	-0.392213
23	C	-4.650719	-3.429748	1.851763	64	C	1.067057	-0.073101	1.572843
24	C	-5.819706	-1.813772	0.482757	65	C	-3.210838	1.990730	1.149743
25	H	-4.720840	-0.036414	-0.015681	66	C	-0.239500	-0.015564	0.891292
26	H	-2.625798	-2.908377	2.394421	67	N	-1.142739	-1.090235	1.118233
27	H	-4.629265	-4.383840	2.371492	68	C	-2.324367	-0.548346	1.167122
28	H	-6.694326	-1.513637	-0.086962	69	C	-2.178739	0.944193	1.066431
29	H	-6.666648	-3.689771	1.124730	70	N	-0.920978	1.236071	0.920355
30	C	0.134925	-0.223831	-1.038961	71	H	3.835685	4.909779	-1.049958
31	N	0.849365	0.972142	-1.316192	72	H	4.475054	0.258335	-2.659422
32	C	2.103446	0.625362	-1.342657	73	H	1.005711	-2.203055	1.875187
33	C	2.200601	-0.857165	-1.137620	74	H	-1.483651	1.727384	-1.950511
34	N	0.998115	-1.347517	-1.033079	75	C	-5.137115	-0.816375	-3.142188
35	C	-1.188983	-0.381381	-1.682336	76	N	-6.248351	-0.935814	-3.467018
36	C	-3.780883	-0.670160	-2.705416	77	O	4.852174	-0.380365	3.290903
37	C	-1.928534	0.746884	-2.067046	78	C	5.703778	0.749631	3.415522
38	C	-1.750319	-1.659585	-1.840739	79	H	5.271716	1.510651	4.078909
39	C	-3.032517	-1.806933	-2.348095	80	H	6.632914	0.377359	3.850858
40	C	-3.216558	0.608895	-2.573919	81	H	5.915499	1.203742	2.437756
41	H	-1.178160	-2.523338	-1.524744					

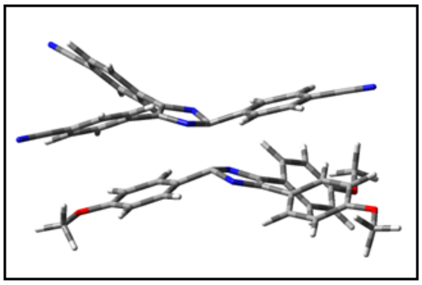

$(CN)_3$-$(OMe)_3$-C2C2_TS

Tag	Symbol	X	Y	Z	Tag	Symbol	X	Y	Z
1	H	-3.122328	-2.795209	-2.713710	47	C	-5.498757	2.739117	1.853490
2	C	-2.759810	-1.791565	-2.527253	48	H	-2.129330	3.295077	0.009862
3	C	-1.802410	0.780135	-1.995078	49	H	-6.434985	2.591408	2.381683
4	C	-1.556336	-1.617003	-1.852999	50	C	-3.639685	-1.435891	0.970408
5	C	-3.505550	-0.668727	-2.918797	51	C	-5.851375	-3.102113	0.521978
6	C	-3.008769	0.618890	-2.662994	52	C	-4.710074	-0.998866	0.172540
7	H	-3.593865	1.474356	-2.985082	53	C	-3.687538	-2.721639	1.535141
8	H	-1.416047	1.772283	-1.794177	54	C	-4.782318	-3.548010	1.320839
9	C	5.177227	3.692655	-1.574370	55	C	-5.807599	-1.821438	-0.052704
10	C	3.018350	3.024406	-0.722516	56	H	-4.666474	-0.028497	-0.306280
11	C	4.406900	1.418589	-1.870604	57	H	-2.847891	-3.063576	2.130773
12	C	5.374175	2.391863	-2.064838	58	H	-4.818378	-4.539525	1.760375
13	C	3.987189	4.007624	-0.900205	59	H	-6.624665	-1.481594	-0.680745
14	H	2.089867	3.264297	-0.214570	60	C	-1.061157	-0.336099	-1.568894
15	H	4.572473	0.420531	-2.261084	61	C	3.211743	1.714652	-1.187093
16	H	6.295144	2.173399	-2.595299	62	C	0.179369	-0.174442	-0.775751
17	H	3.809988	5.004947	-0.515142	63	N	1.075683	-1.286484	-0.767506
18	C	3.474529	-1.595287	-0.680275	64	C	2.268450	-0.775672	-0.836380
19	C	5.682198	-3.270244	-0.189905	65	C	2.150631	0.719790	-0.997511
20	C	4.604384	-1.116281	0.012531	66	N	0.894543	1.048841	-0.974341
21	C	3.472604	-2.930743	-1.107745	67	H	-3.835106	5.110174	0.047418
22	C	4.564901	-3.763779	-0.882072	68	H	-4.743100	0.789151	2.321032
23	C	5.689127	-1.941204	0.262988	69	H	-0.995584	-2.479440	-1.512415
24	H	4.614701	-0.100453	0.389127	70	H	1.272357	1.972581	1.801457
25	H	2.596283	-3.314228	-1.620639	71	C	4.730269	-0.418894	3.685457
26	H	4.536135	-4.786474	-1.239303	72	N	5.804895	-0.501390	4.124241
27	H	6.546885	-1.585995	0.824310	73	C	-6.975644	-3.956945	0.279136
28	C	-0.333242	-0.065179	1.065668	74	N	-7.886164	-4.650683	0.071164
29	N	-1.026291	1.179765	1.113060	75	C	-6.232591	5.002546	1.235071
30	C	-2.288787	0.873574	1.108343	76	N	-7.031554	5.847871	1.254424
31	C	-2.427586	-0.614322	1.121404	77	O	6.191762	4.566843	-1.804248
32	N	-1.243363	-1.150520	1.184679	78	O	-4.721915	-0.723587	-3.526089
33	C	0.926585	-0.145598	1.851459	79	O	6.796864	-3.993352	0.094220
34	C	3.419981	-0.319552	3.115051	80	C	-5.287553	-2.001866	-3.794987
35	C	1.670283	1.014133	2.111150	81	H	-6.250946	-1.806754	-4.268981
36	C	1.428674	-1.392092	2.257681	82	H	-4.657426	-2.584624	-4.479261
37	C	2.663323	-1.482652	2.884020	83	H	-5.444661	-2.572294	-2.870443
38	C	2.910221	0.932608	2.736815	84	C	6.057547	5.902710	-1.335697
39	H	0.856043	-2.284565	2.036759	85	H	5.953147	5.936978	-0.243191
40	H	3.065741	-2.448940	3.169014	86	H	6.976676	6.413724	-1.626869
41	H	3.494470	1.829522	2.915077	87	H	5.198791	6.406875	-1.798266
42	C	-3.329975	1.915331	1.137296	88	C	6.847336	-5.354366	-0.312458
43	C	-5.248041	3.961351	1.208784	89	H	6.780757	-5.451441	-1.404330
44	C	-4.545510	1.727723	1.814826	90	H	7.814633	-5.729912	0.025268
45	C	-3.079473	3.148882	0.511735	91	H	6.045964	-5.943482	0.152933
46	C	-4.027414	4.162334	0.539583					

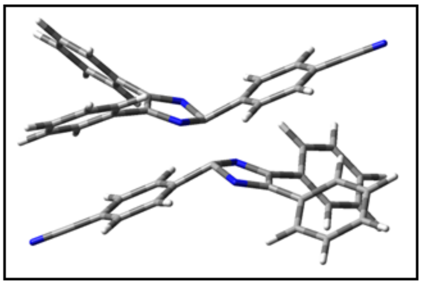

CN-C2C2

Tag	Symbol	X	Y	Z
1	H	3.208625	-2.187806	3.038639
2	C	2.729572	-1.246869	2.789090
3	C	1.523849	1.188027	2.126825
4	C	1.548348	-1.232273	2.056027
5	C	3.321435	-0.036146	3.186891
6	C	2.705291	1.183779	2.861077
7	H	3.162344	2.117612	3.171941
8	H	1.047753	2.121996	1.853800
9	C	-5.545387	3.488919	1.603269
10	C	-3.400060	2.932167	0.638356
11	C	-4.524379	1.351400	2.090857
12	C	-5.573778	2.254407	2.254854
13	C	-4.455366	3.826108	0.795426
14	H	-2.544960	3.181728	0.019215
15	H	-4.551141	0.393751	2.599515
16	H	-6.414448	1.991852	2.890771
17	H	-4.429972	4.786356	0.287747
18	H	-6.369391	4.186801	1.724191
19	C	-3.470625	-1.644894	1.083772
20	C	-5.619848	-3.434954	0.968572
21	C	-4.617032	-1.327387	0.337464
22	C	-3.405208	-2.871821	1.760765
23	C	-4.479824	-3.756858	1.710861
24	C	-5.682939	-2.223569	0.276268
25	H	-4.661999	-0.391814	-0.209956
26	H	-2.506470	-3.117457	2.317842
27	H	-4.426928	-4.700794	2.246272
28	H	-6.559616	-1.975590	-0.315260
29	H	-6.454914	-4.128941	0.926598
30	C	0.258330	-0.007030	-0.786881
31	N	1.078535	1.182205	-0.960369
32	C	2.290173	0.761629	-1.107013
33	C	2.304449	-0.745513	-1.072783
34	N	1.098570	-1.186027	-0.942416
35	C	-0.945360	-0.018862	-1.707398
36	C	-3.321273	-0.040440	-3.187554
37	C	-1.523893	1.185173	-2.128806
38	C	-1.548276	-1.235034	-2.054932
39	C	-2.729396	-1.250625	-2.788143
40	C	-2.705236	1.179930	-2.863213
41	H	-1.101246	-2.160720	-1.713815
42	H	-3.208364	-2.191905	-3.036561
43	H	-3.162293	2.113343	-3.175330
44	C	3.428243	1.680125	-1.280291
45	C	5.545473	3.486680	-1.607098
46	C	4.524550	1.348510	-2.091999
47	C	3.399884	2.931302	-0.641974
48	C	4.455249	3.825004	-0.800002
49	C	5.574011	2.251268	-2.256968
50	H	2.544631	3.181730	-0.023393
51	H	6.414846	1.987816	-2.892297
52	H	6.369523	4.184379	-1.728766
53	C	3.470524	-1.646341	-1.081379
54	C	5.619769	-3.436203	-0.963592
55	C	4.616837	-1.327832	-0.335356
56	C	3.405211	-2.874149	-1.756782
57	C	4.479839	-3.759099	-1.705596
58	C	5.682755	-2.223912	-0.272866
59	H	4.661722	-0.391538	0.210838
60	H	2.506545	-3.120528	-2.313646
61	H	4.427025	-4.703735	-2.239780
62	H	6.559360	-1.975141	0.318437
63	H	6.454845	-4.130117	-0.920616
64	C	0.945307	-0.016576	1.707053
65	C	-3.428282	1.681866	1.278395
66	C	-0.258474	-0.005975	0.786652
67	N	-1.098698	-1.184763	0.943848
68	C	-2.304555	-0.744071	1.073842
69	C	-2.290262	0.763121	1.106095
70	N	-1.078654	1.183495	0.958627
71	H	4.429743	4.785950	-0.293651
72	H	4.551429	0.390152	-2.599317
73	H	1.101325	-2.158414	1.716136
74	H	-1.047878	2.119514	-1.856914
75	C	4.562095	-0.046976	3.904824
76	N	5.575683	-0.058069	4.476049
77	C	-4.561842	-0.052250	-3.905629
78	N	-5.575364	-0.064128	-4.476957

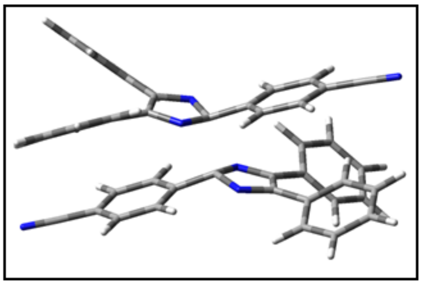

CN-C2C2_TS

Tag	Symbol	X	Y	Z
1	H	-2.748851	-3.253112	-2.599962
2	C	-2.539567	-2.839489	-1.619248
3	C	-1.955724	-1.821602	0.920479
4	C	-1.978209	-1.578345	-1.493041
5	C	-2.800428	-3.609371	-0.470209
6	C	-2.516738	-3.088959	0.802383
7	H	-2.715276	-3.685317	1.686915
8	H	-1.723754	-1.409390	1.894569
9	C	-1.572409	5.147754	3.888005
10	C	-0.722060	3.015273	3.129527
11	C	-2.012906	4.409264	1.627235
12	C	-2.155201	5.359716	2.637271
13	C	-0.858048	3.970654	4.132161
14	H	-0.183503	2.090841	3.310693
15	H	-2.472527	4.577179	0.659239
16	H	-2.722933	6.265897	2.445852
17	H	-0.407904	3.798789	5.106052
18	H	-1.676697	5.893938	4.670908
19	C	-0.986221	3.537910	-1.492251
20	C	-0.625685	5.754161	-3.174197
21	C	-0.302419	4.677292	-1.035194
22	C	-1.483289	3.522083	-2.807102
23	C	-1.313018	4.626657	-3.636264
24	C	-0.115733	5.774276	-1.874952
25	H	0.114750	4.689945	-0.035744
26	H	-1.993276	2.632695	-3.162870
27	H	-1.709483	4.607610	-4.647764
28	H	0.444497	6.632867	-1.516843
29	H	-0.483851	6.610771	-3.827451
30	C	0.986221	-0.240999	-0.098437
31	N	1.106747	-0.925357	1.137540
32	C	1.148624	-2.189826	0.829107
33	C	1.085651	-2.325851	-0.666632
34	N	1.050716	-1.133200	-1.193199
35	C	1.670868	1.059455	-0.223116
36	C	2.800428	3.609371	-0.470209
37	C	1.955724	1.821602	0.920479
38	C	1.978209	1.578345	-1.493041
39	C	2.539567	2.839489	-1.619248
40	C	2.516738	3.088959	0.802383
41	H	1.736293	0.990118	-2.369772
42	H	2.748851	3.253112	-2.599962
43	H	2.715276	3.685317	1.686915
44	C	1.286306	-3.230271	1.859891
45	C	1.572409	-5.147754	3.888005
46	C	2.012906	-4.409264	1.627235
47	C	0.722060	-3.015273	3.129527
48	C	0.858048	-3.970654	4.132161
49	C	2.155201	-5.359716	2.637271
50	H	0.183503	-2.090841	3.310693
51	H	2.722933	-6.265897	2.445852
52	H	1.676697	-5.893938	4.670908
53	C	0.986221	-3.537910	-1.492251
54	C	0.625685	-5.754161	-3.174197
55	C	0.302419	-4.677292	-1.035194
56	C	1.483289	-3.522083	-2.807102
57	C	1.313018	-4.626657	-3.636264
58	C	0.115733	-5.774276	-1.874952
59	H	-0.114750	-4.689945	-0.035744
60	H	1.993276	-2.632695	-3.162870
61	H	1.709483	-4.607610	-4.647764
62	H	-0.444497	-6.632867	-1.516843
63	H	0.483851	-6.610771	-3.827451
64	C	-1.670868	-1.059455	-0.223116
65	C	-1.286306	3.230271	1.859891
66	C	-0.986221	0.240999	-0.098437
67	N	-1.050716	1.133200	-1.193199
68	C	-1.085651	2.325851	-0.666632
69	C	-1.148624	2.189826	0.829107
70	N	-1.106747	0.925357	1.137540
71	H	0.407904	-3.798789	5.106052
72	H	2.472527	-4.577179	0.659239
73	H	-1.736293	-0.990118	-2.369772
74	H	1.723754	1.409390	1.894569
75	C	3.295047	4.947022	-0.604278
76	N	3.665967	6.044370	-0.715860
77	C	-3.295047	-4.947022	-0.604278
78	N	-3.665967	-6.044370	-0.715860

14. Reference

(S1) Iwahori, F.; Hatano, S.; Abe, J. Rational Design of a New Class of Diffusion-Inhibited HABI with Fast Back-Reaction. *J. Phys. Org. Chem.* **2007**, *20* (11), 857–863. https://doi.org/10.1002/poc.1183.

(S2) Plater, M. J. The Crucial Early Contributions of F. R. Japp to a General Synthesis of Imidazole Derivatives. *Bull. Hist. Chem.* **2008**, *33* (2), 76–81.

(S3) Becke, A. D. Density-functional Thermochemistry. III. The Role of Exact Exchange. *J. Chem. Phys.* **1993**, *98* (7), 5648–5652. https://doi.org/10.1063/1.464913.

(S4) Allouche, A. Software News and Updates Gabedit — A Graphical User Interface for Computational Chemistry Softwares. *J. Comput. Chem.* **2012**, *32*, 174–182. https://doi.org/10.1002/jcc.

(S5) Wolinski, K.; Hinton, J. F.; Pulay, P. Efficient Implementation of the Gauge-Independent Atomic Orbital Method for NMR Chemical Shift Calculations. *J. Am. Chem. Soc.* **1990**, *112* (23), 8251–8260. https://doi.org/10.1021/ja00179a005.

(S6) Reed, A. E.; Curtiss, L. A.; Weinhold, F. Intermolecular Interactions from a Natural Bond Orbital, Donor—Acceptor Viewpoint. *Chem. Rev.* **1988**, *88* (6), 899–926. https://doi.org/10.1021/cr00088a005.

(S7) Gaussian 16, Revision C.01, M. J. Frisch, G. W. Trucks, H. B. Schlegel, G. E. Scuseria, M. A. Robb, J. R. Cheeseman, G. Scalmani, V. Barone, G. A. Petersson, H. Nakatsuji, X. Li, M. Caricato, A. V. Marenich, J. Bloino, B. G. Janesko, R. Gomperts, B. Mennucci, H. P. Hratchian, J. V. Ortiz, A. F. Izmaylov, J. L. Sonnenberg, D. Williams-Young, F. Ding, F. Lipparini, F. Egidi, J. Goings, B. Peng, A. Petrone, T. Henderson, D. Ranasinghe, V. G. Zakrzewski, J. Gao, N. Rega, G. Zheng, W. Liang, M. Hada, M. Ehara, K. Toyota, R. Fukuda, J. Hasegawa, M. Ishida, T. Nakajima, Y. Honda, O. Kitao, H. Nakai, T. Vreven, K. Throssell, J. A. Montgomery, Jr., J. E. Peralta, F. Ogliaro, M. J. Bearpark, J. J. Heyd, E. N. Brothers, K. N. Kudin, V. N. Staroverov, T. A. Keith, R. Kobayashi, J. Normand, K. Raghavachari, A. P. Rendell, J. C. Burant, S. S. Iyengar, J. Tomasi, M. Cossi, J. M. Millam, M. Klene, C. Adamo, R. Cammi, J. W. Ochterski, R. L. Martin, K. Morokuma, O. Farkas, J. B. Foresman, and D. J. Fox, Gaussian, Inc., Wallingford CT, 2019.
